# Ruthenium(II) Lipid‐Mimics Drive Lipid Phase Separation to Arouse Autophagy‐Ferroptosis Cascade for Photoimmunotherapy

**DOI:** 10.1002/advs.202411629

**Published:** 2024-11-22

**Authors:** Yue Zheng, Wen‐Jin Wang, Jing‐Xin Chen, Kun Peng, Xiao‐Xiao Chen, Qing‐Hua Shen, Bing‐Bing Liang, Zong‐Wan Mao, Cai‐Ping Tan

**Affiliations:** ^1^ MOE Key Laboratory of Bioinorganic and Synthetic Chemistry, State Key Laboratory of Anti‐Infective Drug Development, IGCME, GBRCE for Functional Molecular Engineering, School of Chemistry Sun Yat‐Sen University Guangzhou 510006 P. R. China; ^2^ Guangdong Province Key Laboratory of Pharmaceutical Bioactive Substances, School of Bioscience and Biopharmaceutics Guangdong Pharmaceutical University Guangzhou 510006 P. R. China

**Keywords:** autophagy, ferroptosis, lipid phase separation, photoimmunotherapy, ruthenium complexes

## Abstract

Lipid‐mediated phase separation is crucial for the formation of lipophilic spontaneous domain to regulate lipid metabolism and homeostasis, furtherly contributing to multiple cell death pathways. Herein, a series of Ru(II) lipid‐mimics based on short chains or midchain lipids are developed. Among them, **Ru‐LipM** with two dodecyl chains significantly induces natural lipid phase separation via hydrocarbon chain‐melting phase transitions. Accompanied by the aggregation of **Ru‐LipM**‐labeled lipophilic membrane‐less compartments, most polyunsaturated lipids are increased and the autophagic flux is retarded with the adaptor protein sequestosome 1 (p62). Upon low‐dose irradiation, **Ru‐LipM** further drives ferritinophagy, providing an additional source of labile iron and rendering cells more sensitive to ferroptosis. Meanwhile, the peroxidation of polyunsaturated lipids occurs due to the deactivation of glutathione peroxidase 4 (GPX4) and the overexpression of acyl‐CoA synthetase long‐chain family member 4 (ACSL4), leading to the immunogenic ferroptosis. Ultimately, both innate and adaptive immunity are invigorated, indicating the tremendous antitumor capability of **Ru‐LipM** in vivo. This study presents an unprecedented discovery of small molecules capable of inducing and monitoring lipid phase separation, thereby eliciting robust immune responses in living cells. It provides a biosimulation strategy for constructing efficient metal‐based immune activators.

## Graphical abstract

An artificial lipid phase separation inducer **Ru‐LipM** is developed, which arouses immunogenic autophagy‐ferroptosis cascade upon irradiation. It is the first report of small molecules that can induce and monitor lipid phase separation to evoke immune responses in living cells, which opens a new avenue for metal‐based immune activators via biosimulation strategy.

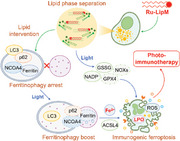

## 1 Introduction

In recent years, phase separations have emerged as a novel approach for bio‐chamber formation, holding great potential to provide specific “workshops” to the “workforce”, namely biomolecules, to participate in intracellular deployment.^[^ ^1^ ^]^ Growing evidences reveal that phase separation can facilitate the construction of intracellular membrane‐less compartments and mediate the crosstalk with membrane‐bounded organelles.^[^ ^2^ ^]^ Owing to the physical barriers, these compartments can activate or inhibit intracellular reactions by locally concentrating biomolecules at distinct sites, providing an innovative road for drug intervention.^[^ ^3^ ^]^ Among those biomolecules, lipids exhibit more lipophilic properties and serve as membrane frames, energy reservoirs and signaling messengers, contributing to inflammation, cancer and other metabolic diseases.^[^ ^4^ ^]^ Typical lipid separation normally occurs in membrane system, known as lipid rafts, which are microdomains enriched in cholesterol, saturated sphingolipids and several proteins.^[^ ^5^ ^]^ The structure of lipid rafts is heterogeneous and rigid, and can partly be broken by steric pressure.^[^ ^6^ ^]^ However, fluid lipid domain which is more amicable for lipid storage and exchange still remains rarely explored.

Lipid is a kind of typical amphiphilic molecule, and its phase separation is the key precursor in amphiphilic self‐assembly in biological systems, which could affect its interaction with other biomolecules.^[^ ^7^ ^]^ But now, precise control of phase separation in fluid co‐assemblies of lipids is still difficult owing to their high miscibility. Due to the versatile properties of metal complexes in morphological, optical and therapeutic aspects,^[^ ^8^ ^]^ metal‐based lipids hold great potential in lipid behavior regulation. Their hydrophilic domains function as phospholipid heads, consisting of metal moieties with catalytic, fluorescent and transportation properties, and also exhibit stronger electronic interaction with natural lipids than organic molecules.^[^ ^9^ ^]^ Hence, such metal‐based lipids can be used to precisely control multi‐lipid co‐assembly processes including miscibility and phase transition. Based on this, Hayami et al. developed an amphiphilic manganese(II) complex lipid to facilitate the formation of micro‐coordination polymers for precise manipulation of rigid artificial lipid domains on the plasma membrane of living cells.^[^ ^10^ ^]^ They further investigated the phenomenon of fluid‐fluid phase separation by co‐assembling single‐chain manganese(II)‐based lipids within natural lipid membranes, emphasizing the crucial role played by metal complex cores in inducing lipid phase separations.^[^ ^11^ ^]^ However, the development of luminescent metal‐based lipids capable of inducing and monitoring lipid phase separations remains unexplored. Motivated by this, we aim to design metal complexes of similar nature and subsequently manipulate the behavior of intracellular lipids.

Local lipid metabolism is first influenced by the unique compositions of lipid‐enriched compartments. In tumor cells, *de novo* lipogenesis, fatty acid (FA) uptake and FA oxidation (FAO) are increased to meet the overwhelming demand of rapid proliferation.^[^ ^12^ ^]^ Lipid metabolism alteration also builds the signaling networks between organelles and cell death pathways. Exogenous lipids will be re‐synthesized into triglycerides (TGs) in the endoplasmic reticulum (ER), modified in Golgi apparatus and stored in lipid droplets (LDs).^[^ ^13^ ^]^ TGs‐containing LDs are sequestered in autophagosomes and delivered to lysosomes, which mediate neutral lipids hydrolyzation for energy supplement during lipophagy.^[^ ^14^ ^]^ Besides, a high‐fat diet also upregulates lipogenesis, lipolysis and FA transport, furtherly inducing ER stress and activating both autophagy and apoptosis.^[^ ^15^ ^]^ Meanwhile, pharmacological perturbation of lipid repair systems involved in glutathione (GSH) and glutathione peroxidase (GPX4) can selectively drive peroxidation of polyunsaturated FA (PUFA)‐containing phospholipids in cancer cells due to the high level of reactive oxygen species (ROS), subsequently evoking ferroptosis.^[^ ^16^ ^]^ Moreover, lipid metabolism also participates in inflammation and immune modulation, which contributes to the immune‐suppressive tumor microenvironments via appropriate intervention.^[^ ^17^ ^]^ These biological processes are closely related to protein phase separation,^[^ ^18^ ^]^ while their relationships with lipid phase separation are still unknown.

**Scheme 1:**
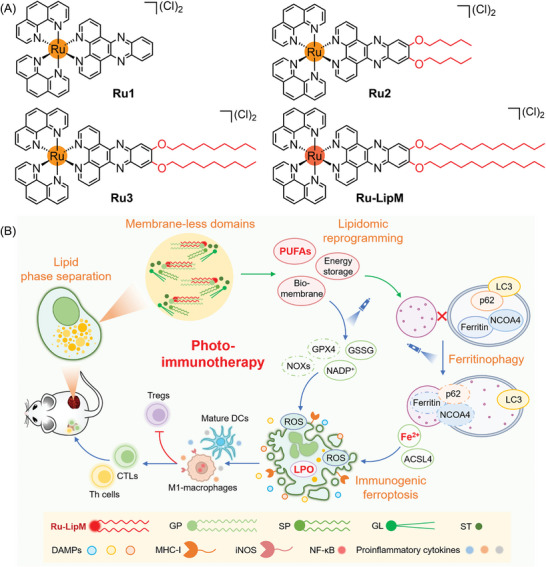
A) Chemical structures of Ru(II) lipid‐mimics. B) The mechanisms of immunotherapy induced by **Ru‐LipM** via lipid phase separation‐mediated autophagy‐ferroptosis cascade. p62: sequestosome 1, LC3: microtubule‐associated protein light chain 3, GSSG: GSH disulfide, NADP^+^: oxidized nicotinamide adenine dinucleotide phosphate (NADPH), NOXs: NADPH oxidases, DCs: dendritic cells, CTLs: cytotoxic T cells, Th cells: helper T cells, Tregs: regulatory T cells, GP: glycerophospholipids. SP: sphingolipids, GL: glycerolipids, ST: serol lipids, DAMPs: damage‐associated molecular patterns, MHC‐I: major histocompatibility complex I, iNOS: nitric oxide synthase, NF‐κB: nuclear factor κ‐light‐chain enhancer of activated B cells.

Herein, we designed three Ru(II) lipid‐mimics based on saturated alkyl chains to intervene tumor lipid metabolism (**Scheme** **1**). As an artificial midchain lipid, **Ru‐LipM** can induce natural lipid phase separation via hydrocarbon chain‐melting phase transitions to generate lipophilic membrane‐less domains, accompanied by polyunsaturated lipid enrichment and ferritinophagy arrest. Upon low‐dose irradiation, **Ru‐LipM** triggers autophagic degradation of ferritin within autolysosomes and activates the Fenton reaction to peroxide the polyunsaturated lipids. This process is accompanied by the inactivation of GPX4 and upregulation of acyl‐CoA synthetase long‐chain family member 4 (ACSL4), ultimately leading to the immunogenic ferroptosis. Mighty innate and adaptive immune responses evoked by **Ru‐LipM** indicate its tremendous antitumor capability in vivo. To the best of our knowledge, this is the first report of small molecules that can induce and monitor lipid phase separation in living cells, subsequently eliciting an immune response through light irradiation, which builds a new avenue for metal‐based immune activators via biosimulation strategy.

## 2 Results and Discussion

### 2.1 Synthesis and characterization

These Ru(II)‐based lipids were synthesized and purified according to the procedure of **Ru1** with modifications.^[^ ^19^ ^]^ The precursor *cis*‐[Ru(phen)_2_Cl_2_] (phen: 1,10‐phenanthroline) was reacted with corresponding dipyrido[3,2‐*a*:2′,3′‐*c*]phenazine (dppz) derivatives with different length of hydrocarbon chains (**Ru2**: 11,12‐bis(pentyloxy)dipyrido[3,2‐*a*:2′,3′‐*c*]phenazine (bpdppz); **Ru3**: 11,12‐bis(nonyloxy)dipyrido[3,2‐*a*:2′,3′‐*c*]phenazine (bndppz); **Ru‐LipM**: 11,12‐bis(dodecyloxy)dipyrido[3,2‐*a*:2′,3′‐*c*]phenazine (bdodppz)) in aqueous ethanol at 80 °C for 6 h under nitrogen protection (Scheme S1). The dppz derivatives and Ru(II) complexes were characterized by ESI‐MS, ^1^H‐NMR, ^13^C‐NMR and elemental analysis (Figures S1–S21, Supporting Information).

**Figure 1:**
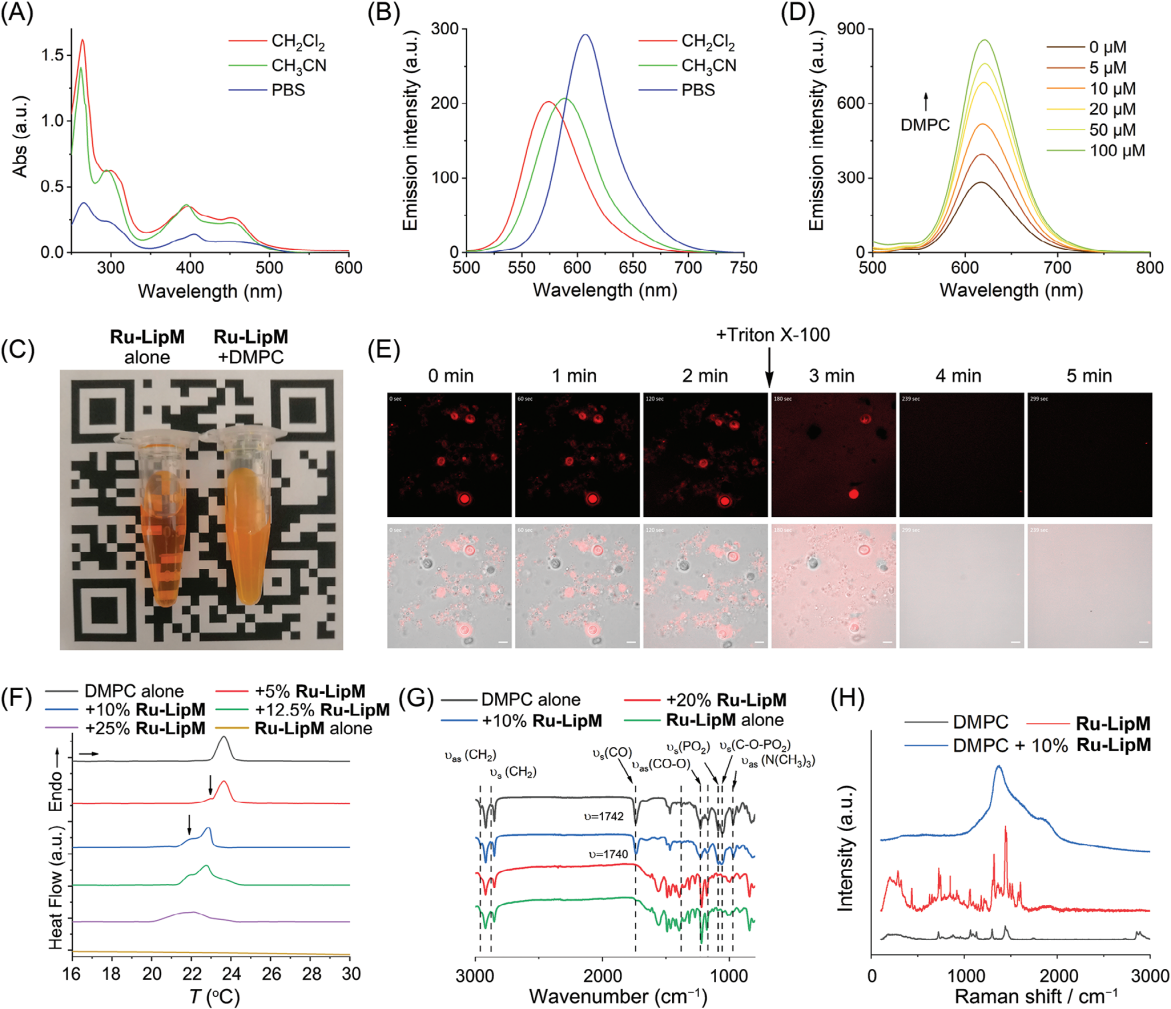
A) UV–vis absorption spectra of **Ru‐LipM** (10 µM) in degassed CH_2_Cl_2_, CH_3_CN and PBS at 298 K. B) Emission spectra of **Ru‐LipM** (10 µM) in degassed solvents at 298 K. The excitation wavelength is 450 nm. C) The turbidity assays of DMPC (100 µM) and **Ru‐LipM** (10 µM) in 2% Tween‐20 buffer recorded by digital camera. D) Emission spectra of **Ru‐LipM** (10 µM) in 2% Tween‐20 buffer with different concentrations of DMPC (0–100 µM). E) Phospholipid phase separation in DMPC/**Ru‐LipM** mixtures at indicated time intervals in vitro recorded by laser scanning confocal microscope. Scale bar: 10 µm. λ_ex_ = 488 nm; λ_em_ = 607 ± 20 nm. F) DSC, G) IR and H) Raman spectrum of DMPC and DMPC/**Ru‐LipM** mixtures with different ratios.

UV–vis spectra of Ru(II) complexes in degassed CH_2_Cl_2_, CH_3_CN and phosphate buffered saline (PBS) show characteristic absorption peaks for π‐π* ligand‐centered excited states (λ_max_ = 368–401 nm) and metal‐to‐ligand charge transfer (MLCT) transitions (λ_max_ = 448–464 nm) (**Figure** **1**A; Figure S22, Supporting Information).^[^ ^19^, ^20^ ^]^ UV–vis spectroscopy measurement also shows that the absorbance of Ru(II) complexes is proportional to concentration up to 200 µM, indicating the sufficient solubility at working concentrations (Figure S23, Supporting Information). **Ru1**–**Ru3** are less‐luminescent in aqueous solutions and show strong emission in non‐protonic solvents, which is similar to reported Ru(II) complexes (Figure S24 and Table S1, Supporting Information).^[^ ^21^ ^]^ However, **Ru‐LipM** exhibits a polarity‐dependent solvent relaxation in organic solvents due to the decreased emission energy (Figure 1B; Figure S25, Supporting Information),^[^ ^22^ ^]^ while displaying conspicuous phosphorescence in aqueous solution (Figure 1B), which can be attributed to its self‐assembly properties facilitated by the sufficiently long dodecyl chain (Figure S26, Supporting Information). All of these Ru(II) complexes feature abilities to produce singlet oxygen (^1^O_2_), as shown in Figure S27 and Table S1 (Supporting Information). Unexpectedly, the quantum yield of ^1^O_2_ (*Ф*_△_) for **Ru‐LipM** in aerated tween buffer experiences a significant reduction after mixing with the natural lipid 1,2‐dimyristoyl‐sn‐glycero‐3‐phosphocholine (DMPC, Figure S28, Supporting Information), as detected by hydrophilic 9,10‐anthracenedipropionic acid (ABDA). Conversely, the *Ф*_△_ of **Ru‐LipM**‐DMPC mixtures in DMSO shows slight alteration when measured using lipophilic 1,3‐diphenylisobenzofuran (DPBF, Figure S29, Supporting Information). Considering that ABDA can only detect ^1^O_2_ in the aqueous phase,^[^ ^23^ ^]^ while DPBF is suitable for ^1^O_2_ detection in the organic phase,^[^ ^24^ ^]^ this phenomenon can be attributed to the formation of a lipid‐rich domain induced by **Ru‐LipM** within the tween buffer. This lipid‐rich domain also dissolves a portion of ^1^O_2_,^[^ ^25^ ^]^ rendering it undetectable by water‐soluble ABDA. However, the ^1^O_2_ in lipidic environment can be captured by DPBF dissolved in DMSO.

### 2.2 **Ru‐LipM** Induces Phase Separation via Hydrocarbon Chain‐Melting Phase Transitions

A turbid suspension of lipids containing long fatty chains, namely DMPC and 1,2‐dihexadecanoyl‐rac‐glycero‐3‐phosphocholine (DPPC), is observed upon the addition of **Ru‐LipM** (Figure 1C; Figure S30, Supporting Information). However, a similar phenomenon is not observed in the mixture of **Ru‐LipM** and cholesterol (Figure S30, Supporting Information). Furthermore, the phenomenon is also not evident for **Ru1**−**Ru3** (Figure S31, Supporting Information), suggesting that the intriguing capability of **Ru‐LipM** to induce lipid co‐assembly may be attributed to its interaction with long fatty chains. The emission of **Ru‐LipM** is correspondingly enhanced with the increase in DMPC content during this process, which differs from previous metal‐based lipids,^[^ ^10^, ^11^ ^]^ thereby highlighting the potential of **Ru‐LipM** to monitor the lipid dynamics (Figure 1D). Subsequently, almost uniform fluorescent coacervate microdroplets with mobility are observed by confocal microscopy (Figure 1E and Movie S1, Supporting Information), and the fusions of those microdroplets are also found in the same condition (Movie S2, Supporting Information). The fluid phase separated in the microdroplets is validated by the dissolution phenomenon with a detergent, Triton X‐100.^[^ ^26^ ^]^ Moreover, the hybrids of DMPC/**Ru‐LipM** (5%) and DMPC/**Ru‐LipM** (10%) exhibit two distinct peaks in the differential scanning calorimeter (DSC) analysis, indicating that the addition of a small amount of **Ru‐LipM** induced phase separations within the DMPC system (Figure 1F). The melting points gradually decrease as the proportion of **Ru‐LipM** increases in the DMPC/**Ru‐LipM** mixture, indicating that **Ru‐LipM** can hybridize with DMPC and disturb the lipid packings.^[^ ^11^, ^27^ ^]^

Infrared (IR) spectroscopy demonstrates an intermolecular interaction formed between DMPC and **Ru‐LipM** (Figure 1G). Accompanied by the addition of **Ru‐LipM**, the pristine peaks of the hydrophilic phosphate head of DMPC (_s_ν_CO_, _s_ν_PO2_, _s_ν_C‐O‐PO2_ and _as_ν_(N(CH3)3)_) gradually diminish, indicating significant the intermolecular interactions between DMPC and **Ru‐LipM** at these specific sites. Moreover, the broadening and upward shifts in the _s_ν_CH2_ (2850 cm^−1^) and _as_ν_CH2_ (2930 cm^−1^) of DMPC are detected, which reflects the increased hydrocarbon chain conformational disorder and mobility due to the onset of gauche rotamer formation and the concomitant decline in the number of all‐trans rotamers present upon chain melting.^[^ ^11^, ^28^ ^]^ The presence of a similar result is observed in DPPC but not in cholesterol, which highlights the essential role of long fatty chains for phase separation induced by **Ru‐LipM** (Figure S32, Supporting Information). Meanwhile, no analogous findings are detected in the DMPC‐**Ru1**/**Ru2**/**Ru3** systems (Figure S33, Supporting Information). In Raman spectra, the characteristic peaks assigned to the stretching modes of –CH_3_ and –CH_2_– become broadened and enhanced, whereas corresponding bending modes are disappeared, which further proves the disorder and mobility in DMPC/**Ru‐LipM** system (Figure 1H). These results demonstrate that **Ru‐LipM** can induce the lipid phase separation in vitro by intermolecular interaction‐induced hydrocarbon chain‐melting phase transitions.

### 2.3 Ru(II) Complexes Induce Photo‐Antiproliferative Effects

The in vitro dark‐ and photo‐antiproliferative activities of **Ru‐LipM** and **Ru1**–**Ru3** were evaluated in cervical cancer (HeLa and U14), small cell lung cancer (A549 and LLC), breast cancer (MDA‐MB‐231 and 4T1) and two normal (lung fibroblast (HLF) and mammary epithelial (MCF‐10A)) cell lines (**Table** **1** and Table S2, Supporting Information).

**Table 1:** IC_50_ (µM) values of **Ru‐LipM** toward different cell lines^a)^.

Cell lines	IC_50_ (µM)				
	dark	light‐low^b)^	light‐high^c)^	PCI‐low	PCI‐high
HeLa	>50.0	2.28±0.12	0.08±0.01	>21.9	>625.0
U14	39.4±4.68	1.43±0.06	0.05±0.01	27.6	788.0
A549	37.6±2.89	2.48±0.12	0.22±0.03	>15.2	170.9
LLC	>50.0	1.99±0.08	0.15±0.01	>25.1	>333.3
MDA‐231	35.1±5.33	2.16±0.21	0.09±0.01	16.2	390.0
4T1	>50.0	2.37±0.33	0.12±0.02	>21.1	>416.7
MCF‐10A	>50.0	6.01±0.18	0.43±0.05	>8.3	>116.3
HLF	>50.0	5.17±0.29	0.25±0.02	>9.7	>200.0

^a)^ Data are presented as the means ± standard deviations (SD), and antiproliferative activity was assessed after 72 h of incubation (n = 3).

^b)^ Low‐light dose: 450 nm, 17 mW cm^−2^, 2 J cm^−2^.

^c)^ Normal‐light dose: 450 nm, 17 mW cm^−2^, 15 J cm^−2^.

The highest photo‐cytotoxicity index (PCI) value is obtained for **Ru‐LipM** in cervical cancer cells in both low‐ and normal‐light doses (> 625.0 for HeLa cells and 788.0 for U14 cells, respectively). The highest dark‐antiproliferative activities are observed for **Ru2**, and it also displays certain photo‐antiproliferative activities. **Ru1** exhibits low antiproliferative activities under both dark and light conditions. According to the results obtained from inductively coupled plasma mass spectrometry (ICP‐MS, Figure S34, Supporting Information), apart from **Ru1**, the antiproliferative ability of these complexes in neoplastic cells is not solely determined by their uptake efficiency. It is also associated with phase separation‐induced anti‐oxidative stress responses that safeguard cells against damage.^[^ ^29^ ^]^ Meanwhile, **Ru‐LipM** shows a relative selectivity for neoplastic cells over normal cells. The selectivity can be ascribed to the differences in its uptake levels between neoplastic and normal cells (Figure S34, Supporting Information), which may arise from the heightened electronegativity and accelerated metabolic rate of neoplastic cells.^[^ ^30^ ^]^

### 2.4 **Ru‐LipM** Induces and Monitors Membrane‐Less Lipid Domain Formation

**Figure 2:**
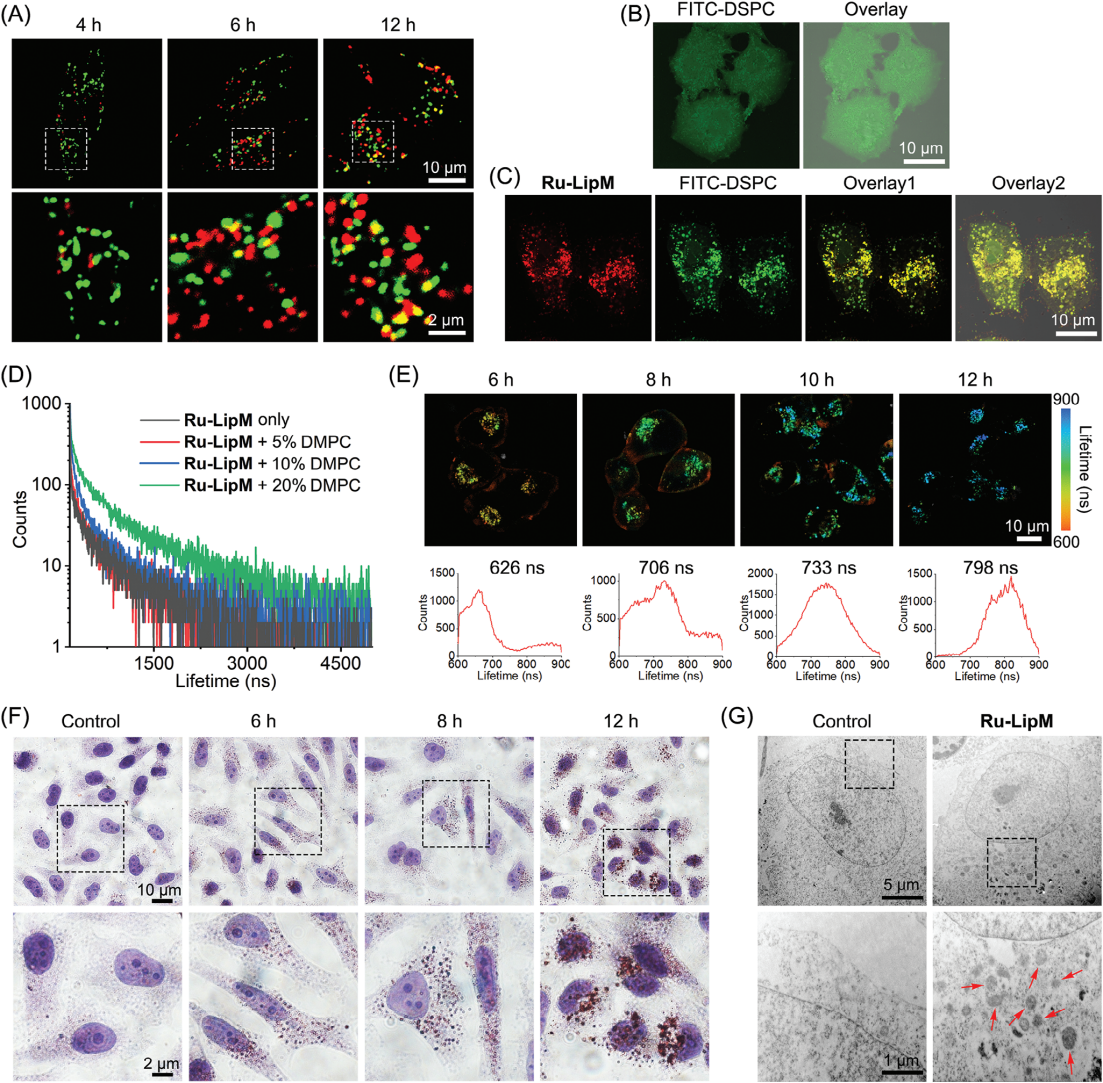
A) Colocalization of **Ru‐LipM** with LTDR. HeLa cells were treated with **Ru‐LipM** (10 µM) for different time intervals (4, 6, and 12 h) and stained with LTDR (200 nM, 15 min). Confocal images of FITC‐DSPE labeled HeLa cells B) before and C) after incubation with **Ru‐LipM** (10 µM) for 12 h. D) The lifetime decay curves of **Ru‐LipM** (10 µM) with DMPC in 2% Tween‐20 buffers. E) The TPPLIM images of HeLa cells treated with **Ru‐LipM** (10 µM) at different time intervals (6, 8, 10, and 12 h). F) Oil red O & hematoxylin double staining in HeLa cells exposed to **Ru‐LipM** (10 µM) at different time intervals (6, 8, and 12 h). G) TEM imaging of HeLa cells without negative staining treated with **Ru‐LipM** (10 µM) for 12 h. **Ru‐LipM**: λ_ex_ = 488 nm; λ_em_ = 607 ± 20 nm. LTDR: λ_ex_ = 633 nm; λ_em_ = 668 ± 20 nm. FITC‐DSPE: λ_ex_ = 488 nm; λ_em_ = 521 ± 20 nm.

Confocal microscopic observation shows that **Ru‐LipM** mainly localizes on cell membranes after 1 h incubation, while has a punctate distribution in the cytoplasm after prolonging the incubation time to 3 h (Figure S35, Supporting Information). However, **Ru‐LipM** exhibits minimal colocalization with commercial organelle‐specific dyes including lysosome tracker deep red (LTDR), LD tracker deep red (LD‐TDR), red fluorescence protein‐fused peroxisome plasmid (Peroxisome‐RFP), mitochondrion tracker deep red (MTDR) and ER tracker red (ERTR; Figure S36, Supporting Information). Upon prolonged incubation, fusion events were observed between **Ru‐LipM** and re‐synthesized (ER)/recycled (lysosome) lipid organelles (**Figure** **2**A; Figure S37, Supporting Information), which implies that **Ru‐LipM** may alter intracellular lipid metabolism via phase separation.^[^ ^31^ ^]^ ICP‐MS analysis also reveals that **Ru‐LipM** predominantly localizes in cytoplasm, with a fraction of it residing in the membrane after 6 h treatment. Subsequently, translocation of **Ru‐LipM** to ER is observed when the incubation time extends to 12 h due to the natural membrane fluxion (Figure S38, Supporting Information).

FITC‐labeled 1,2‐distearoyl‐sn‐glycero‐3‐phosphocholine (DSPC) shows disseminated green fluorescence throughout whole cells with no specific organelle staining (Figure 2B). After **Ru‐LipM** treatment, the fluorescence pattern transforms into puncta, which is similar to the phenomena detected for the lipid phase separation model in living cells (Figure 2C).^[^ ^32^ ^]^ Excellent co‐localization of **Ru‐LipM** with FITC‐DSPC (Pearson's colocalization coefficients (PCC): 0.78) suggests that the phenomenon is caused by **Ru‐LipM**. Moreover, steady–state emission shows that the lifetime of **Ru‐LipM** is positively correlated with the molar ratio of [DMPC]/[**Ru‐LipM**], and it gradually increases from 531.25 ns (**Ru‐LipM** only) to 775.36 ns when [DMPC]/[**Ru‐LipM**] reaches 20% (Figure 2D and Table S3, Supporting Information). Two‐photon phosphorescence lifetime imaging (TPPLIM) reveals that **Ru‐LipM** exhibits a punctate emission distribution in the cytoplasm with a lifetime of 626 ns after 6 h incubation (Figure 2E). As the incubation time extends to 8 and 10 h, the phosphorescence lifetime of **Ru‐LipM** gradually increases to 706 and 733 ns, respectively. After 12 h incubation, the lifetime of **Ru‐LipM** reaches 798 ns, which aligns with the lifetime of **Ru‐LipM** in phase‐separated lipids in vitro (Figure 2D and Table S3, Supporting Information). The results demonstrate the potential of **Ru‐LipM** to induce and monitor lipid phase separation simultaneously in living cells.

Furtherly, intracellular spherical domains are significantly increased upon **Ru‐LipM** intervention (Figure S39, Supporting Information). The cellular hyperspectral images reveal a predominant co‐localization of **Ru‐LipM** with these spherical structures, which convincingly indicate the presence of **Ru‐LipM** within these domains (Figure S40, Supporting Information). Oil red O & hematoxylin double staining assay reveals the lipophilic nature of these domains, indicating a time‐dependent increase in cellular lipid storage (Figure 2F). Meanwhile, cellular TG content, the major component in lipid energy reservoir, is also increased as measured by the enzyme‐linked immunosorbent assay (ELISA, Figure S41, Supporting Information). Considering the lipid droplets also serve as storage organelles for TG, a time‐dependent imaging analysis of lipid droplets was conducted. The results demonstrate that no significant increase in lipid droplet abundance is observed in **Ru‐LipM**‐treated cells (Figure S42, Supporting Information). Moreover, non‐negative staining transmission electron microscope (TEM) images show the formation of heavily colored membrane‐less domains in **Ru‐LipM**‐treated cells (red arrows), which is the typical characterization of cellular phase separation. It's reasonable due to the high Z number of ruthenium, similar to the commonly used fixing agent OsO_4_ and staining agent uranyl acetate/lead citrate (Figure 2G). Together, these findings manifest that **Ru‐LipM** can stimulate membrane‐less lipid reservoir domain formation and increase neutral lipid contents.

### 2.5 **Ru‐LipM** Reprograms Lipid Metabolism in Cancer Cells

Lipid metabolomics analysis shows that lipids from **Ru‐LipM**‐treated groups (with/without irradiation) and control group are clearly separated into three clusters with good quality (Figure S43, Supporting Information). Orthogonal partial least squares discriminant analysis (OPLS‐DA) score plots show a distinct lipid profile for samples subjected to different treatments (Figure S44, Supporting Information).

**Figure 3:**
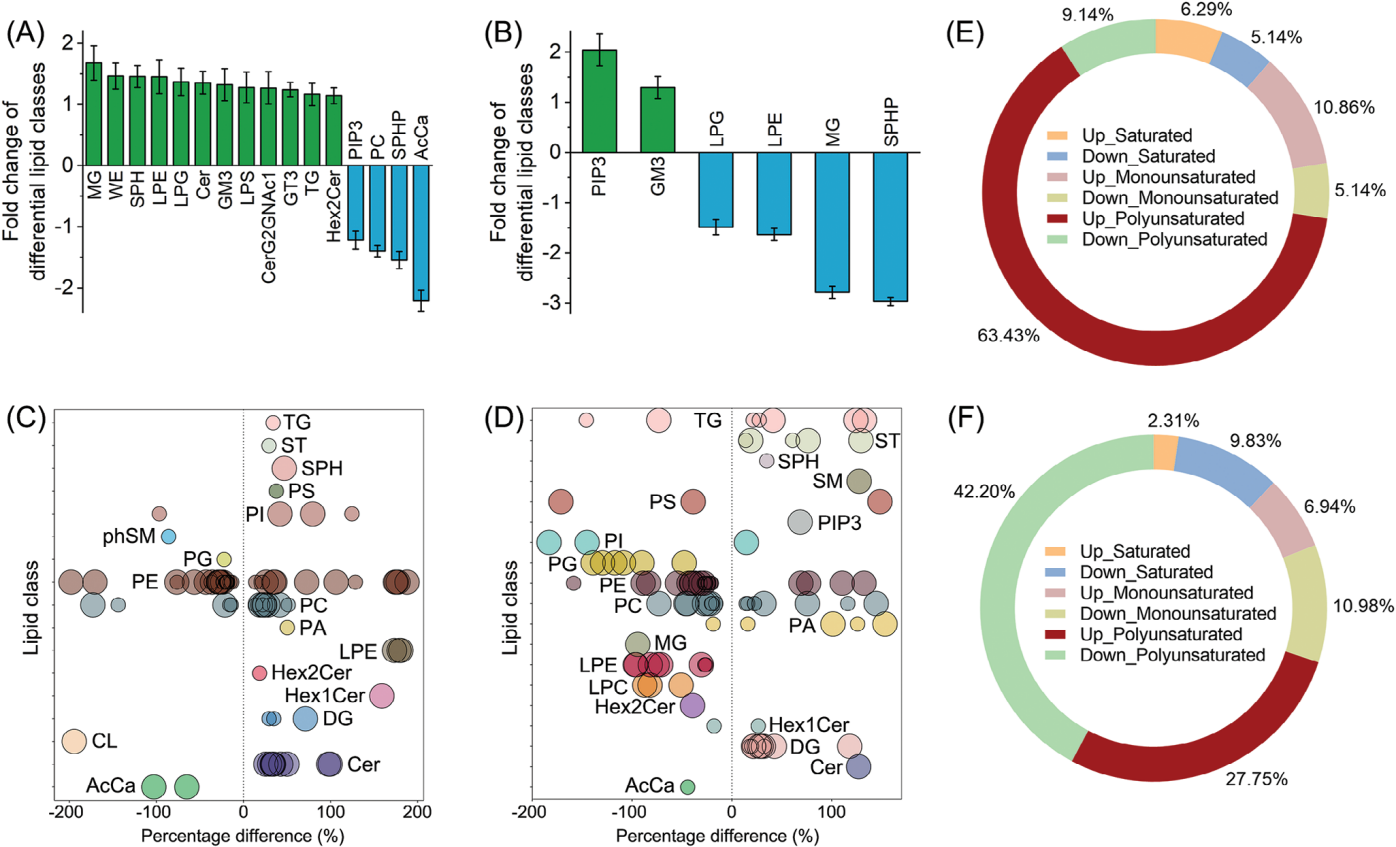
A,B) The impact of **Ru‐LipM** (10 µM, 12 h) on the content of different classes of lipids in HeLa cells. C,D) Bubble Plots of the lipid species that are significantly enriched in **Ru‐LipM**‐treated cells. E,F) The structural characteristic of the impact of **Ru‐LipM** treatment on lipid species. A, C and E) **Ru‐LipM**_dark group versus control group. B, D and F) **Ru‐LipM**_light group versus **Ru‐LipM**_dark group. CerG2NAc1: simple glucosylceramide series, Hex1Cer, Hex2Cer: hexose ceramides, PIP3: phosphatidylinositol bisphosphates, PS: phosphatidylserine, PI: phosphatidylinositol, phSM: phytosphingosine, AcCa: acyl carnitine, PG: phosphatidylglycerol, PA: phosphatidic acid, CL: cardiolipins, SM: sphingomyelin, LPC: lysophosphatidylcholine. Irradiation condition: 450 nm, 17 mW cm^−2^, 2 J cm^−2^. Standard: The variable importance (VIP) > 1, FC > 1.5 or < 0.67, *P* value < 0.05.

Different from the other polar metabolites, lipid is diverse and distinct, which has been divided into eight categories containing distinct classes and subclasses of molecules.^[^ ^33^ ^]^ A total of 33 lipid classes and 1455 lipid species are identified from lipid extracts (Figure S45, Supporting Information). After **Ru‐LipM** treatment in dark, lipids involved in ER‐mediated bio‐membrane construction, phase separation and energy storage are increased, especially in categories of glycerophospholipids (GP),^[^ ^34^ ^]^ sphingolipids (SP),^[^ ^35^ ^]^ glycerolipids (GL)^[^ ^36^ ^]^ and sterol lipids (ST)^[^ ^37^ ^]^ (Table S4, Supporting Information). Corresponding lipid classes are also enriched, including energy‐related monoglyceride (MG), TG and wax esters (WE), membrane‐related lysophosphatidylethanolamine (LPE), lysophosphatidylglycerol (LPG) and sphingosine (SPH), lipid raft‐related gangliosides (GM3 and GT3), and immune‐related ceramide (Cer) and lysophosphatidylserine (LPS) (**Figure** **3**A and Table S5, Supporting Information). Furtherly, the reduced LPE, LPG, sphingomyelin (SPHP) and MG indicate that **Ru‐LipM** contributes to the damage of bio‐membrane and rearrangement of energy storage after irradiation (450 nm, 17 mW cm^−2^, 2 J cm^−2^) (Figure 3B and Table S5, Supporting Information).

Based on a standard of fold change (FC) > 1.5 or < 0.67 and *P* value < 0.05, **Ru‐LipM** treatments cause 228 differential lipid species (DLSs) (Up‐regulated: 176; Down‐regulated: 52) and 153 DLSs (Up‐regulated: 44; Down‐regulated: 109) in the absence and presence of light, respectively (Figure S46, Supporting Information). In dark, **Ru‐LipM** specifically upregulates those lipid species involved in TG, phosphatidylethanolamine (PE), phosphatidylcholine (PC) and LPE, especially for TG(20:5/14:3/14:3), PE(18:1/22:6), PC(16:1/22:6), PC(12:1e/22:6) and LPE(22:4), which are closely associated with autophagosome formation,^[^ ^38^ ^]^ lipid storage and biosynthesis (Figure 3C and Data S1, Supporting Information). Upon light irradiation, **Ru‐LipM** significantly reduces the levels of PI(24:1_10:1), PS(38:2), PG(40:9), PE(37:3e), TG(18:1_18:1_18:1). These findings suggest the lipolysis of GP, which is the primary lipid category in cellular membranes (Figure 3D and Data S2, Supporting Information). Further analysis shows that most of the altered lipid species are characterized by long (C atoms > 12, Figure S47, Supporting Information) polyunsaturated chains (Figure 3E), including kinds of gangliosides, PC and PE, which are the main constituents of lipid phase transition and reactants of lipid peroxidation.^[^ ^39^ ^]^

According to the lipid saturation analysis, treatment with **Ru‐LipM** in darkness leads to an up‐regulation of ≈80.58% of lipid species, including 63.43% of PUFAs. Conversely, when combined with light exposure, **Ru‐LipM** significantly decreases the ratio of PUFAs (Figure 3E,F), suggesting potential lipid degradation during the irradiation process. These results show that **Ru‐LipM** induces a lipidomic reprogramming by depositing lipid storage and intervening membrane synthesis in dark, and promotes PUFAs degradation upon irradiation.

### 2.6 **Ru‐LipM** Initiates Multiple Immunogenic Autophagy‐Ferroptosis Cascade

**Figure 4:**
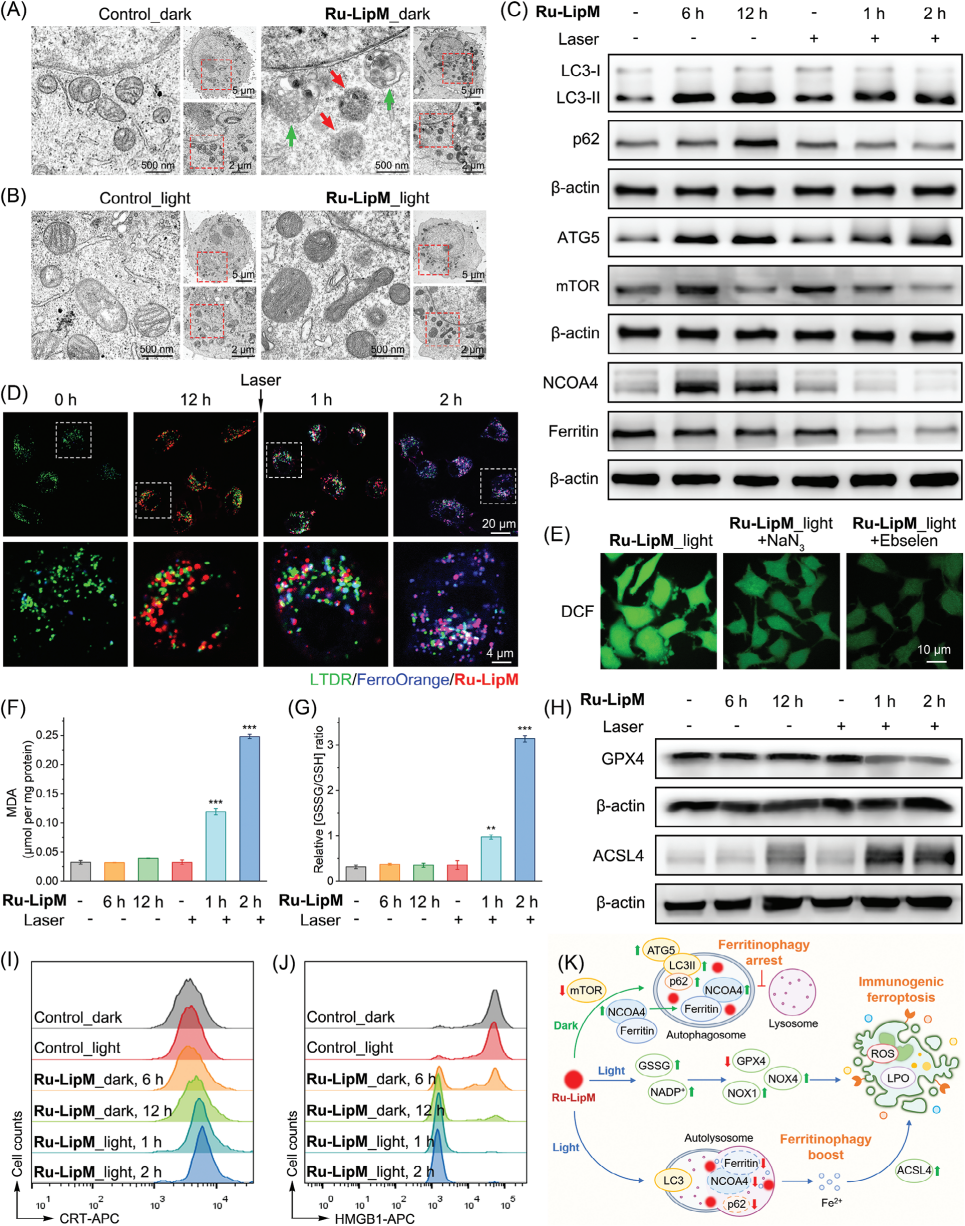
TEM images of HeLa cells treated with 10 µM **Ru‐LipM** A) for 12 h in the dark and B) then incubated for another 2 h after light irradiation. C) Western analysis of the impact of **Ru‐LipM** (10 µM) on the expression of proteins in HeLa cells. D) Confocal images of HeLa cells labeled with LTDR (200 nm) and FerroOrange (0.5 µM) after **Ru‐LipM** (10 µM) treatment. **Ru‐LipM**: λ_ex_ = 488 nm, λ_em_ = 607 ± 20 nm. LTDR: λ_ex_ = 633 nm, λ_em_ = 668 ± 20 nm. FerroOrange: λ_ex_ = 561 nm, λ_em_ = 595 ± 20 nm. E) Impact of **Ru‐LipM** (10 µM) on ROS levels by H_2_DCFDA staining and confocal microscopy. DCF: λ_ex_ = 488 nm; λ_em_ = 525 ± 20 nm. Impact of **Ru‐LipM** (10 µM) on F) the malondialdehyde (MDA, oxidative lipid product) level and G) the ratios of cellular GSSG/GSH. H) The impact of **Ru‐LipM** (10 µM) on the expression of GPX4 and ACSL4 in HeLa cells. Flow cytometric analysis of the expression of I) etco‐CRT and J) intracellular HMGB1 after **Ru‐LipM** treatments (10 µM) in HeLa cells. K) Proposed mechanisms by which **Ru‐LipM** induces autophagy‐ferroptosis cascade. Irradiation condition: 450 nm, 17 mW cm^−2^, 2 J cm^−2^. Error bars: S.D., n = 3. *p < 0.05, **p < 0.01, ***p < 0.001 by the unpaired Student's two‐tailed *t* test.

Since **Ru‐LipM** alters lipid metabolism in cancer cells, the downstream death processes were investigated.^[^ ^40^ ^]^ TEM analysis reveals that in the absence of light, **Ru‐LipM** induces the formation of both membrane‐less domains (**Figure** **4**A, indicated by red arrow) and autophagosomes (indicated by green arrow) in HeLa cells. Upon irradiation, autophagosomes containing membrane‐less domains fuse with lysosomes to form autolysosomes for degradation (Figure S48, Supporting Information). By prolonging the incubation time, **Ru‐LipM**‐treated cells display typical ultrastructural characteristics of ferroptosis including shrunken mitochondria, increased density of bilayer membrane and intact nucleus (Figure 4B). Based on this, the autophagy‐ferroptosis cascade induced by **Ru‐LipM** was studied in detail.

The subtype transformation of microtubule‐associated protein light chain 3 (LC3), the upregulation of autophagy‐related gene 5 (ATG5), and the inhibition of mechanistic target of rapamycin (mTOR) are considered reliable indicators for the initiation, formation and elongation processes of autophagosome biogenesis,^[^ ^41^ ^]^ while the accumulation of sequestosome 1 (p62) is regarded as an indicative sign of autophagic flux inhibition,^[^ ^42^ ^]^ which also exhibits close association with phase separation and protective antioxidation.^[^ ^29^ ^]^ Meanwhile, NCOA4, a cargo protein responsible for delivery of ferritin to autophagosome for subsequent iron release, mediates ferritinophagy and causes ferroptosis amplification.^[^ ^43^ ^]^ Upon **Ru‐LipM** treatment, an increase in the levels of LC3‐II, p62, ATG5 and mTOR is detected. Additionally, NCOA4 is also found to be upregulated while ferritin remained unchanged (Figure 4C; Figure S49, Supporting Information). These findings demonstrate the impairment of ferritinophagic flux, which is accompanied by the translocation of ferritin. In combination with light, **Ru‐LipM** boosts the flux to promote the autolysosome formation, as proved by the degradations of NCOA4 and ferritin (Figure 4C; Figure S49, Supporting Information). Co‐staining of the ferrous ions specific dye, FerroOrange, with LTDR and **Ru‐LipM** also reveals a gradual release of ferrous ions in lysosomes following light irradiation (Figure 4D). This observation indicates the degradation of ferritin during autophagosome‐lysosome fusion. Subsequently, the released ferrous ions are transported into the cytoplasm over an extended incubation period, leading to an overload of labile iron pool in **Ru‐LipM**‐treated cells.

2′,7′‐dichlorodihydrofluorescein diacetate (H_2_DCFDA) staining shows that **Ru‐LipM** treatment markedly promotes ROS production in combination with light. Pre‐incubation of ebselen (lipid hydroperoxides (LPO) scavenger) and NaN_3_ (^1^O_2_ scavenger) can eliminate the acquired ROS, indicating the contributions of those two ROS species in **Ru‐LipM**‐induced cell death (Figure 4E; Figure S50, Supporting Information). A time‐dependent increase in LPO levels is correspondingly detected in **Ru‐LipM**‐treated cells in the presence of light (Figure 4F), accompanied by an increase in the ratios of GSH disulfide (GSSG)/GSH (Figure 4G) and oxidized nicotinamide adenine dinucleotide phosphate (NADP^+^)/NADPH (Figure S51, Supporting Information). Further investigation reveals that NADPH oxidases, including NOX1 and NOX4, are upregulated in **Ru‐LipM**‐treated cells under light exposure, leading to the generation of ROS through one‐electron trans‐membrane transfer to molecular oxygen (Figure S52, Supporting Information).^[^ ^44^ ^]^ Additionally, the depletion of the anti‐peroxidative guardian GPX4 and the upregulation of the fatty acid‐associated ferroptosis promoter ACSL4^[^ ^16a^ ^]^ are observed in **Ru‐LipM**‐treated cells, providing further evidence for the occurrence of ferroptosis (Figure 4H; Figure S53, Supporting Information). To validate more cell death modes of **Ru‐LipM**, different inhibitors were applied (Figure S54, Supporting Information). Pretreatment with necroptotic inhibitor necrosulfonamide, pyroptotic inhibitor disulfiram or necrotic inhibitor necrostatin‐1 results in minimal changes in cell viability. However, pre‐incubation with autophagic inhibitor 3‐methyladenine and ferroptotic inhibitor ferrostatin‐1 significantly improves cell viability. In addition, the apoptotic inhibitor z‐VAD‐fmk also increases the cell survival rate, as evidenced by the downregulation of anti‐apoptotic protein B‐cell lymphoma‐2 (Bcl‐2) and upregulation of proapoptotic protein Bcl‐2‐associated X (BAX, Figure S55, Supporting Information), indicating that **Ru‐LipM** can induce multiple pathways leading to cell death.

Ferroptosis typically exhibits robust immunogenicity accompanied by the release of damage‐associated molecular patterns (DAMPs) during the early stage, but not in the late stage.^[^ ^45^ ^]^ The lack of immunogenicity observed in late‐stage ferroptotic cells may be attributed to their abundant presence of oxidized lipids, which contribute to reduced phagocytosis and impaired antigen cross‐presentation by dendritic cells (DCs), thereby favoring tumor evasion.^[^ ^46^ ^]^ Additionally, apoptotic cells can also elicit an immunogenic response through the secretion of cytokines and chemokines.^[^ ^47^ ^]^ Thus, tumor immunogenicity is characterized by the release of nuclear high‐mobility group box 1 protein (HMGB1), cell‐surface exposure of calreticulin (etco‐CRT) and extracellular secretion of adenosine triphosphate (ATP).^[^ ^48^ ^]^ Flow cytometric measurements show that the intracellular fluorescence from etco‐CRT and HMGB1 in **Ru‐LipM**‐treated cells is increased and decreased in a time‐dependent manner, respectively (Figure 4I,J; Figures S56 and S57, Supporting Information). Simultaneously, the extracellular ATP is also increased (Figure S58, Supporting Information). These results indicate **Ru‐LipM** intervenes ferritinophagic flux and iron homeostasis, which further initiates the autophagy‐ferroptosis cascade by light irradiation, coherently leading to immunogenic responses (Figure 4K).

### 2.7 **Ru‐LipM** Modulates Tumor Metabolism and Immunity

RNA‐sequence (seq) analysis was further performed to clarify the impact of **Ru‐LipM** on transcriptome with or without light irradiation. The correlation coefficients between every two individual samples from the same group are above 0.97 (Figure S59A,B, Supporting Information), implying the good reproducibility. The overall Q30 percentage is above 92.4%. More than 95.9% of readings are mapped to reference genes in all samples (Table S6, Supporting Information) and 86.5% of readings are located in exons (Figure S59C,D, Supporting Information). As compared with control samples, the total DGEs (differential expression genes; FC > 1.5 or < 0.67; False Discovery Rate (FDR) < 0.01; Figure S60A and Data S3, Supporting Information) for **Ru‐LipM**‐treated cells in dark and under irradiation are 5032 (Up‐regulated: 2657; Down‐regulated: 2375) and 8470 (Up‐regulated: 4161; Down‐regulated: 4309) genes, respectively (Figure S60B and Data S4, Supporting Information).

**Figure 5:**
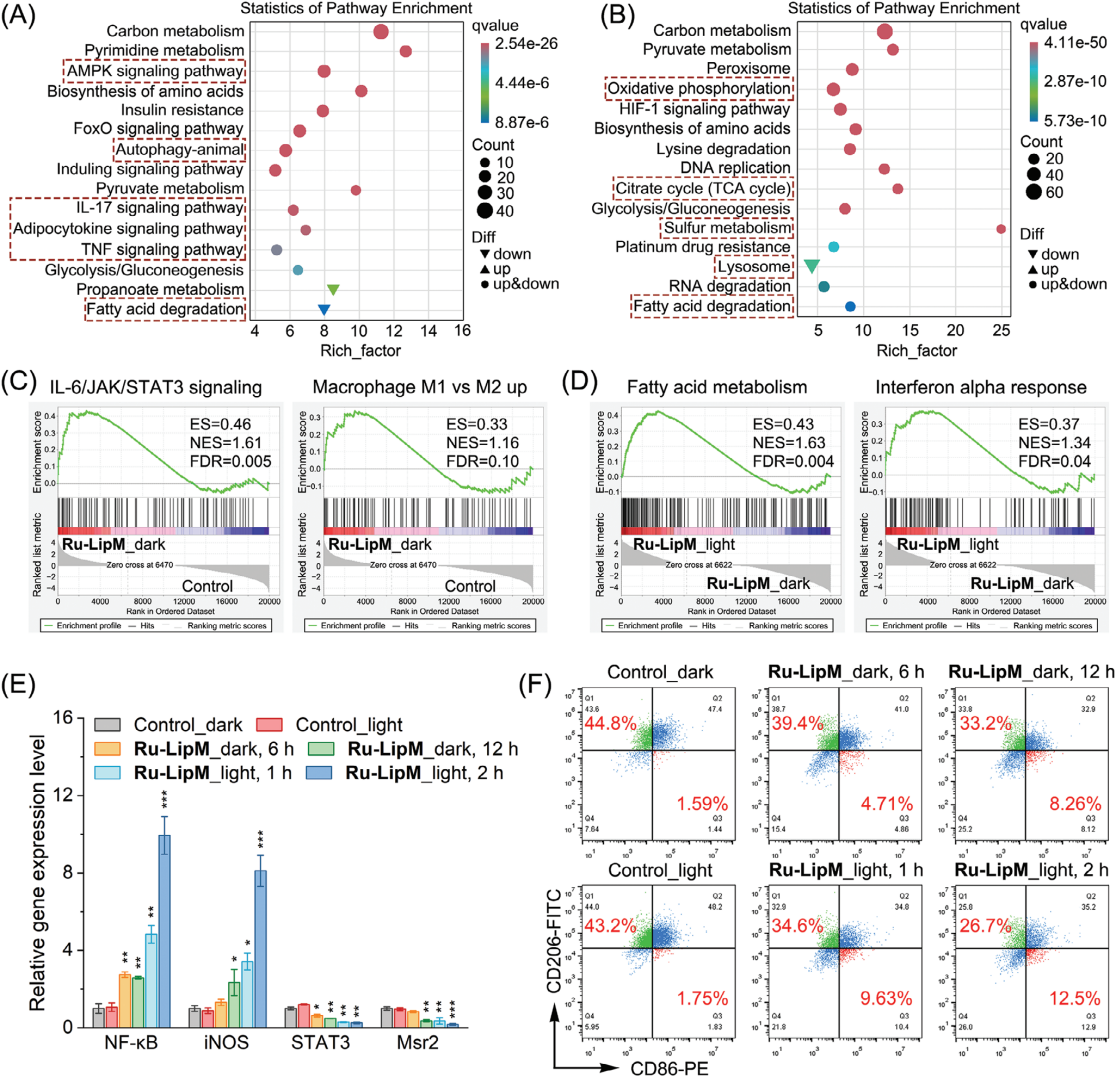
A,B) KEGG enrichment analysis of pathways influenced by **Ru‐LipM** treatment (10 µM, 12 h). C,D) GSEA reveals the enrichment of **Ru‐LipM**‐altered genes in various bio‐processes. A,C) **Ru‐LipM**_dark group versus control group. B,D) **Ru‐LipM**_light group versus **Ru‐LipM**_dark group. ES: enrichment score. NES: normalized ES. FDR: false positive rate. E) Gene expressions of NF‐κB, iNOS, STAT3 and Msr2 in mouse myeloid‐derived M2 macrophages. M2 macrophages were incubated with mouse cervical cancer (U14) cell supernatant treated with **Ru‐LipM** for 12 h. F) Flow cytometric analysis of the levels of M1‐macrophage marker (CD11b^+^F4/80^+^CD86^+^) and M2‐macrophage marker (CD11b^+^F4/80^+^CD206^+^) in mouse myeloid‐derived M2 macrophages. Irradiation condition: 450 nm, 17 mW cm^−2^, 2 J cm^−2^. Error bars: S.D., n = 3. *p < 0.05, **p < 0.01, ***p < 0.001 by the unpaired Student's two‐tailed *t* test.

Gene Ontology analysis shows that **Ru‐LipM** in dark causes significant overall changes in gene categories including metabolic process, signaling, membrane and antioxidant activity, consisting with the results of lipidomic reprogramming (Figure S61, Supporting Information). When combined with irradiation, violent decreases of gene expression are found in biological regulation, response to stimulus, membrane part and binding are induced by **Ru‐LipM**, which synergistically cause the cellular peroxidative environments (Figure S62, Supporting Information). Kyoto Encyclopedia of Genes and Genomes (KEGG) pathway annotation indicates that **Ru‐LipM** influences various pathways including adenosine 5′‐monophosphate‐activated protein kinase (AMPK), autophagy, interleukin (IL)‐17, tumor necrosis factor (TNF)‐α, adipocytokine and down‐regulated fatty acid degradation in dark (**Figure** **5**A). **Ru‐LipM** treatment in combination with light obstructs lysosome pathway, degrades fatty acids and influences energy‐related oxidative phosphorylation, citrate cycle and ferroptosis‐related sulfur metabolism (Figure 5B).

Gene Set Enrichment Analysis (GSEA) furtherly shows that **Ru‐LipM** preferentially invigorates genes favorable for the IL‐6/JAK/STAT3 inflammatory cascade signaling and the macrophage phenotype transformation from anti‐inflammatory M2 state to tumor‐suppressed M1 phenotype (Figure 5C). In addition, **Ru‐LipM** combined with light not only heightens fatty acid metabolism, but also promotes immune response via enhancing interferon α response, which arouses major histocompatibility complex I (MHC‐I) on the tumor surface (Figure 5D). Quantitative reverse transcription polymerase chain reaction (RT‐qPCR) confirms that nuclear factor κ‐light‐chain enhancer of activated B cells (NF‐κB) and nitric oxide synthase (iNOS), two genes favorable for converting macrophages to M1 phenotype,^[^ ^49^ ^]^ are up‐regulated after **Ru‐LipM** treatments in M2‐macrophages (Figure 5E). Macrophage scavenger receptor 2 (Msr2) and signal transducer and activator of transcription 3 (STAT3), two genes that induce macrophage polarization to M2 phenotype, are suppressed as expected. Besides, **Ru‐LipM** heightens the level of two MHC‐I genes in HeLa cells, human leukocyte antigen (HLA)‐A and transporter associated with antigen processing 1 (TAP1) in combination with light, which also contributes to the enhancement of tumor immunogenicity (Figure S63, Supporting Information).^[^ ^50^ ^]^ Correspondingly, the ratio of CD206^+^ macrophage (M2) decreases from ≈44.8% to 26.7% after **Ru‐LipM** treatment, accompanied by an increase of CD86^+^ macrophage (M1) from ≈1.59% to 12.5% (Figure 5F; Figure S64, Supporting Information). These results suggest that **Ru‐LipM** can transcriptionally modulate tumor metabolism and immunity, participating in the signal transmission in tumor microenvironment.

### 2.8 **Ru‐LipM** Exhibits Potent Antitumor Photo Immunotherapeutic Effects In Vivo

**Figure 6:**
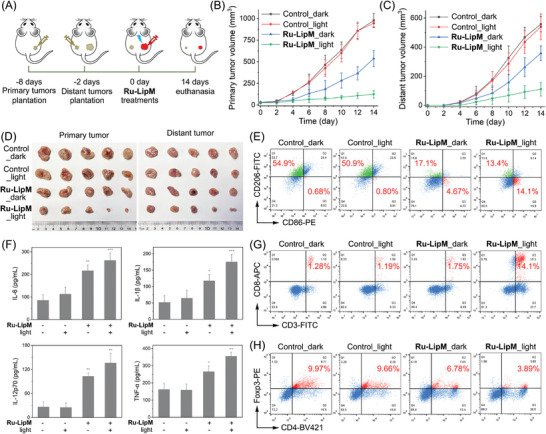
A) Schematic illustration of in vivo therapeutic protocol. The volume curves of B) primary tumors and C) distant tumors. D) Represented images of tumorous sections in primary tumors and distant tumors. E) Flow cytometric measurement of the proportions of M1‐macrophages (CD11b^+^F4/80^+^CD86^+^) and M2‐macrophages (CD11b^+^F4/80^+^CD206^+^) in distant tumors. F) ELISA measurement of the levels of IL‐6, IL‐1β, IL‐12p70 and TNF‐α in mouse serum. G and H) Flow cytometric detection of populations of CD8^+^ G) and Foxp3^+^ H) T cells in distant tumors. Irradiation condition: 450 nm, 17 mW cm^−2^, 2 J cm^−2^. Error bars: S.D., n = 5. *p < 0.05, **p < 0.01, ***p < 0.001 by the unpaired Student's two‐tailed *t* test.

Encouraged by the mighty immunogenicity achieved by **Ru‐LipM** in vitro, the antitumor immunity was assessed in the immunocompetent mouse model as scheduled in **Figure** **6**A. U14 cells were inoculated to the BALB/c mice as primary tumors. 6 days later, U14 cells were planted into the contralateral site as distant tumors. The growth of both primary and distant tumors is successfully inhibited upon **Ru‐LipM** treatment (Figure 6B−D; Figure S65, Supporting Information) with no significant weight change (Figure S66, Supporting Information) or organ damage (Figure S67, Supporting Information) detected during treatment.

In order to investigate the antitumor immune mechanism, several kinds of immune cells were analyzed. The proportion of M1‐macrophages (CD11b^+^F4/80^+^CD86^+^) increases from ≈0.68% to 4.67%, accompanied by decreased content of M2‐macrophages (CD11b^+^F4/80^+^CD206^+^) from ≈54.9% to 17.1% (Figure 6E; Figure S68, Supporting Information). In combination with light, **Ru‐LipM** ulteriorly aggravates the M1‐polarization of macrophages with a ratio of 13.4% M2‐macrophages and 14.1% M1‐macrophages. Remarkable increases in pro‐inflammatory cytokines (IL‐6, IL‐1β, IL‐12p70 and TNF‐α), secreted from M1‐macrophages as well as mature DCs, are also detected in serum under both dark and light conditions (Figure 6F).

The percentage of CD80^+^CD86^+^ cells, which are hallmarks for DC maturation and benefit tumor antigen presentation, significantly increases from ≈9.33% to 32.5% when combined with light (Figures S69 and S71, Supporting Information), contributing to the initiation of adaptive antitumor immunity. The sufficient infiltration of cytotoxic T cells (CTLs, CD8^+^ T cells) in tumor area is one of the prerequisites for adaptive immunity activation.^[^ ^51^ ^]^ Under light condition, the percentage of CD8^+^ T cells increases from ≈1.19% to 14.1% after **Ru‐LipM** treatment (Figure 6G). Besides, the proportion of helper T cells (Th cells, CD4^+^ T cells) under the same condition (≈20.1%) is 5.7‐fold relative to that in control group (≈3.51%; Figures S70 and S71, Supporting Information), which benefits to CD8^+^ T cells activation. One of the special subtype of CD4^+^ T cells, regulatory T cells (Tregs, CD4^+^Foxp3^+^ T cells), contributing in immune escape via inducing apoptosis of CD8^+^ T cells and M2‐polarization of macrophages,^[^ ^52^ ^]^ is obviously decreased from 9.66% to 3.89% after **Ru‐LipM** treatment combined with light (Figure 6H; Figure S71, Supporting Information). These results verify that **Ru‐LipM**‐mediated therapies can effectively evoke potent immune responses in vivo.

## 3 Conclusion

In summary, we combine Ru(II) metal framework with lipid‐mimic ligands that can induce lipid phase separation to intervene lipid metabolism, leading to multiple enhancements of tumor immunogenicity and robust antitumor immunity. We develop three Ru(II) complexes based on saturated alkyl chains, only the one with long dodecyl chains, **Ru‐LipM**, can induce lipid phase separation on account of strong hydrocarbon chain‐melting phase transitions. Subsequently, **Ru‐LipM** induces plenty of lipophilic membrane‐less domain formation with excess polyunsaturated lipid storage, resulting in cellular autophagic flux arrest. In combination with low‐dose light, **Ru‐LipM** furtherly boosts the autophagic process, providing excess labile iron by NCOA4‐mediated ferritin degradation and making polyunsaturated lipids degradation. Owning to the deactivation of antioxidative guardian GPX4 and the upregulation of fatty acid‐associated ferroptosis promoter ACSL4, intracellular LPO level is rapidly heightened and immunogenic ferroptosis is activated after **Ru‐LipM** treatment in combination with light, resulting in both innate and adaptive immunity enhancement in vivo. This work provides a new way to build metal‐based immune activators via biosimulation strategy, potentially offering new perspectives for immunotherapeutics via lipid phase separation.

## 4 Experimental Section

### Synthesis and Characterization

**Ligand bpdppz**: ^1^H NMR (500 MHz, Chloroform‐*d*_1_) *δ* (ppm) 9.48 (dd, *J* = 8.1, 1.8 Hz, 1H), 9.21 (dd, *J* = 4.4, 1.8 Hz, 1H), 7.72 (dd, *J* = 8.1, 4.4 Hz, 1H), 7.43 (s, 1H), 4.26 (t, *J* = 6.6 Hz, 2H), 2.00 (dt, *J* = 14.6, 6.8 Hz, 2H), 1.52 (ddt, *J* = 43.5, 14.9, 7.0 Hz, 4H), 0.99 (t, *J* = 7.2 Hz, 3H).^13^C NMR (126 MHz, Chloroform‐*d*_1_) *δ* (ppm) 154.16, 151.69, 147.54, 140.54, 138.50, 133.14, 127.84, 123.93, 106.59, 77.41, 77.16, 76.91, 69.47, 28.69, 28.41, 22.62, 14.22. ESI‐MS m/z (CH_3_OH/CH_3_CN): calculated for [M+H]^+^ (C_28_H_30_N_4_O_2_) 455.24, found 455.62; calculated for [2M+Na]^+^ 931.46, found 931.41. Elemental analysis calcd (%) for C_28_H_30_N_4_O_2_: C, 73.98; H, 6.65; N, 12.33; found: C, 73.71; H, 6.87; N, 12.14.

**Ligand bndppz**: ^1^H NMR (600 MHz, Chloroform‐*d*_1_) *δ* (ppm) 9.54 (dd, *J* = 8.1, 1.8 Hz, 2H), 9.23 (dd, *J* = 4.3, 1.8 Hz, 2H), 7.75 (dd, *J* = 8.1, 4.4 Hz, 2H), 7.47 (s, 2H), 4.27 (t, *J* = 6.6 Hz, 4H), 1.99 (p, *J* = 6.8 Hz, 4H), 1.58 (p, *J* = 7.3 Hz, 4H), 1.44 (p, *J* = 7.3, 6.8 Hz, 4H), 1.37–1.26 (m, 16H), 0.89 (t, *J* = 6.8 Hz, 6H). ^13^C NMR (151 MHz, Chloroform‐*d*_1_) *δ* (ppm) 154.35, 151.73, 147.63, 140.70, 138.65, 133.25, 127.98, 123.97, 106.79, 77.37, 77.16, 76.95, 69.56, 32.06, 29.74, 29.55, 29.43, 29.05, 26.24, 22.83, 14.23. ESI‐MS m/z (CH_3_OH/CH_3_CN): calculated for [M+H]^+^ (C_36_H_46_N_4_O_2_) 567.37, found 567.71; calculated for [2M+Na]^+^ 1155.71, found 1155.47. Elemental analysis calcd (%) for C_36_H_46_N_4_O_2_: C, 76.29; H, 8.18; N, 9.89; found: C, 76.14; H, 8.03; N, 9.72.

**Ligand bdodppz**: ^1^H NMR (600 MHz, Chloroform‐*d*_1_) *δ* (ppm) 9.62 (d, *J* = 20.8 Hz, 2H), 9.54–9.40 (m, 2H), 8.04 (dd, *J* = 8.0, 4.4 Hz, 2H), 7.37 (d, *J* = 15.4 Hz, 2H), 4.31–4.21 (m, 4H), 1.99 (p, *J* = 7.0 Hz, 4H), 1.58 (t, *J* = 7.6 Hz, 4H), 1.44 (p, *J* = 7.2 Hz, 4H), 1.30 (dp, *J* = 21.2, 9.1, 7.9 Hz, 28H), 0.88 (t, *J* = 6.8 Hz, 6H). ^13^C NMR (151 MHz, Chloroform‐*d*_1_) *δ* (ppm) 155.32, 149.68, 141.28, 136.78, 136.37, 128.97, 125.66, 106.45, 77.37, 77.16, 76.95, 69.77, 32.08, 29.87, 29.83, 29.81, 29.58, 29.52, 29.02, 26.25, 22.83, 14.22. ESI‐MS m/z (CH_3_OH/CH_3_CN): calculated for [M+H]^+^ (C_42_H_58_N_4_O_2_) 651.46, found 651.97; calculated for [2M+Na]^+^ 1323.90, found 1323.82. Elemental analysis calcd (%) for C_42_H_58_N_4_O_2_: C, 77.50; H, 8.98; N, 8.61; found: C, 77.35; H, 8.81; N, 8.53.

The precursor *cis*‐[Ru(phen)_2_Cl_2_]•2H_2_O and **Ru1** were synthesized as literature methods.^[^ ^19^ ^]^

**[Ru(phen)_2_(dppz)](Cl)_2_ (Ru1)**: A suspension of *cis*‐[Ru(phen)_2_Cl_2_]•2H_2_O (0.5 mmol) and dppz (0.6 mmol) in 20 mL EtOH/H_2_O (v/v = 1/1) was added to a round‐bottom flask (50 mL). And then the reaction was refluxed under N_2_ atmosphere for 6 h. After the reaction finished, the solvent was evaporated and the crude products were purified through flash chromatograph on silica (MeCN/H_2_O/20% KCl, v/v/v = 90/10/1). The target fractions were collected and concentrated, followed by addition of a small amount of saturated NaCl solution. Then products were filtered and washed with H_2_O and ether, dried in vacuo to obtain **Ru1** as a red solid. Yield 250.0 mg (61.4%). ^1^H NMR (400 MHz, DMSO‐*d*_6_) *δ* (ppm) 9.62 (dd, *J* = 12.2, 7.9 Hz, 2H), 8.81 (q, *J* = 7.8, 7.1 Hz, 4H), 8.52 (dd, *J* = 6.4, 3.3 Hz, 2H), 8.40 (q, *J* = 6.9, 5.2 Hz, 4H), 8.28 (d, *J* = 5.0 Hz, 2H), 8.19 (d, *J* = 5.5 Hz, 4H), 8.06 (d, *J* = 5.1 Hz, 2H), 7.92 (dt, *J* = 8.2, 4.8 Hz, 2H), 7.86–7.73 (m, 4H). ^13^C NMR (101 MHz, DMSO‐*d*_6_) *δ* (ppm) 153.98, 153.31, 152.70, 150.77, 147.15, 141.95, 140.23, 136.99, 133.23, 132.62, 130.50, 130.48, 130.11, 129.46, 128.10, 127.57, 126.38, 126.28. ESI‐MS m/z (CH_3_OH): calculated for [M−2Cl]^2+^ (C_42_H_26_N_8_Ru) 372.06, found 372.65; calculated for [M−Cl]^+^ 744.13, found 743.64. Elemental analysis calcd (%) for C_42_H_26_Cl_2_N_8_Ru•H_2_O: C, 60.58; H, 3.39; N, 13.46; found: C, 60.32; H, 3.01; N, 13.59.

**[Ru(phen)_2_(bpdppz)](Cl)_2_ (Ru2)**: was synthesized by a method similar to what described for **Ru1** by replacing the dppz with bpdppz. Yield 229.3 mg (46.5%). ^1^H NMR (400 MHz, DMSO‐*d*_6_) *δ* (ppm) 9.52 (d, *J* = 8.1 Hz, 2H), 8.86–8.76 (m, 4H), 8.41 (s, 4H), 8.27 (d, *J* = 5.1 Hz, 2H), 8.16 (d, *J* = 5.1 Hz, 2H), 8.07 (d, *J* = 5.1 Hz, 2H), 7.89 (dd, *J* = 8.0, 5.5 Hz, 2H), 7.79 (dq, *J* = 10.1, 5.4 Hz, 4H), 7.69 (s, 2H), 4.32 (t, *J* = 6.0 Hz, 4H), 1.86 (p, *J* = 6.3 Hz, 4H), 1.54–1.49 (m, 4H), 1.41 (dt, *J* = 14.2, 7.1 Hz, 4H), 0.93 (t, *J* = 7.2 Hz, 6H). ^13^C NMR (101 MHz, DMSO‐*d*_6_) *δ* (ppm) 154.72, 153.18, 152.48, 149.39, 147.98, 147.92, 140.49, 137.14, 135.41, 135.35, 132.68, 130.09, 129.89, 129.81, 129.61, 129.10, 128.15, 128.05, 126.50, 125.99, 106.34, 69.14, 27.95, 27.76, 21.81, 13.89. ESI‐MS m/z (CH_3_OH): calculated for [M−2Cl]^2+^ (C_52_H_46_N_8_O_2_Ru) 458.14, found 458.40; calculated for [M−Cl]^+^ 951.25, found 951.26. Elemental analysis calcd (%) for C_52_H_46_Cl_2_N_8_O_2_Ru: C, 63.28; H, 4.70; N, 11.35; found: C, 62.99; H, 4.58; N, 11.24.

**[Ru(phen)_2_(bndppz)](Cl)_2_ (Ru3)** was synthesized by a method similar to what described for **Ru1** by replacing the dppz with bndppz. Yield 211.9 mg (38.6%). ^1^H NMR (400 MHz, DMSO‐*d*_6_) *δ* (ppm) 9.52 (d, *J* = 8.2 Hz, 2H), 8.84–8.77 (m, 4H), 8.42 (s, 4H), 8.28 (d, *J* = 5.0 Hz, 2H), 8.16 (d, *J* = 5.0 Hz, 2H), 8.07 (d, *J* = 5.0 Hz, 2H), 7.92–7.87 (m, 2H), 7.83–7.76 (m, 4H), 7.69 (s, 2H), 4.32 (t, *J* = 6.3 Hz, 4H), 1.85 (q, *J* = 6.7, 6.2 Hz, 4H), 1.56–1.51 (m, 4H), 1.38 (d, *J* = 6.7 Hz, 4H), 1.32–1.24 (m, 16H), 0.86 (t, *J* = 6.3 Hz, 6H). ^13^C NMR (101 MHz, DMSO‐*d*_6_) *δ* (ppm) 154.73, 153.19, 152.48, 149.39, 147.93, 140.50, 137.14, 135.40, 135.35, 132.68, 130.09, 129.89, 129.82, 129.61, 129.10, 128.16, 128.06, 127.44, 126.51, 125.98, 106.32, 69.12, 31.27, 29.01, 28.72, 28.60, 28.31, 25.56, 22.05, 13.86. ESI‐MS m/z (CH_3_OH): calculated for [M−2Cl]^2+^ (C_60_H_62_N_8_O_2_Ru) 514.20, found 514.47; calculated for [M−Cl]^+^ 1063.37, found 1063.39. Elemental analysis calcd (%) for C_60_H_62_Cl_2_N_8_O_2_Ru: C, 65.56; H, 5.69; N, 10.19; found: C, 65.69; H, 5.87; N, 10.10.

**[Ru(phen)_2_(bdodppz)](Cl)_2_ (Ru‐LipM)** was synthesized by a method similar to what described for **Ru1** by replacing the dppz with bdodppz. Yield 273.9 mg (46.3%). ^1^H NMR (500 MHz, DMSO‐*d*_6_) *δ* (ppm) 9.53 (dd, *J* = 8.3, 1.3 Hz, 2H), 8.78 (ddd, *J* = 8.1, 6.5, 1.3 Hz, 4H), 8.40 (s, 4H), 8.25 (dd, *J* = 5.3, 1.3 Hz, 2H), 8.15 (dd, *J* = 5.3, 1.3 Hz, 2H), 8.06 (dd, *J* = 5.2, 1.3 Hz, 2H), 7.88 (dd, *J* = 8.3, 5.4 Hz, 2H), 7.78 (ddd, *J* = 13.0, 8.3, 5.3 Hz, 4H), 7.71 (s, 2H), 4.33 (t, *J* = 6.1 Hz, 4H), 1.87 (h, *J* = 7.0, 6.5 Hz, 4H), 1.53 (p, *J* = 7.3 Hz, 4H), 1.39 (p, *J* = 7.0 Hz, 4H), 1.33–1.20 (m, 28H), 0.85–0.80 (m, 6H). ^13^C NMR (101 MHz, Chloroform‐*d*_1_) *δ* (ppm) 177.59, 155.45, 154.58, 153.87, 153.33, 152.98, 152.06, 149.21, 147.71, 147.46, 141.46, 137.27, 137.05, 134.14, 133.16, 130.87, 130.50, 128.42, 128.23, 127.59, 126.76, 106.36, 69.74, 32.04, 29.85, 29.80, 29.78, 29.56, 29.50, 28.97, 26.25, 24.75, 22.80, 14.23. ESI‐MS m/z (CH_3_OH): calculated for [M−2Cl]^2+^ (C_66_H_74_N_8_O_2_Ru) 566.25, found 566.23; calculated for [M−Cl]^+^ 1147.47, found 1147.46. Elemental analysis calcd (%) for C_66_H_74_Cl_2_N_8_O_2_Ru: C, 66.99; H, 6.30; N, 9.47; found: C, 67.21; H, 6.47; N, 9.66.

### UV–vis and Fluorescence Data Measurement

The UV–vis absorption spectra of all Ru(II) complexes (10 µM) were measured in CH_2_Cl_2_, CH_3_CN and PBS using Agilent Varian Cary 300 spectrophotometer (USA) at 298 K. Fluorescence data were recorded on Edinburgh FLS980 spectrophotometer (UK) combined fluorescence lifetime and steady–state spectrometer (Japan) with 450 nm excitation. All solvents were degassed with N_2_ for 20 min before using. All complexes were dissolved in DMSO just before the experiments, and the concentration of DMSO in spectral experiments was 1% (v/v).

### Solubility Measurement

Solubility studies of all Ru(II) complexes were monitored by UV–vis spectroscopy. The complex was first dissolved in DMSO and then diluted with PBS to 3.125−200 µM. The absorption spectra were recorded at 298 K and the absorbance of maximum absorptive wavelength at MLCT bands was fitted.

### ^1^O_2_ Production

The quantum yields of ^1^O_2_ production (*Ф*_△_) of all Ru(II) complexes under irradiation (450 nm, 17 mW cm^−2^) in PBS buffer were evaluated using ABDA (100 µM) as ^1^O_2_ indicator.^[^ ^23^ ^]^ [Ru(bpy)_3_]Cl_2_ (*Ф*_△_ = 0.18 in H_2_O) was served as standard. The absorption maxima of ABDA (380 nm) were recorded every 4 s. To further investigate the lipid influence on ^1^O_2_ production, **Ru‐LipM** was pre‐mixed with DMPC at indicated concentrations (5, 10, 20, 50, and 100 µM) in 2% Tween‐20 buffer or DMSO before irradiation. The absorption maxima of ABDA (380 nm) or DPBF (418 nm) were recorded every 3 s. In DMSO system, methylene blue (*Ф*_△_ = 0.52) was used as a reference for ^1^O_2_ sensitization.^[^ ^53^ ^]^

### Turbidity Analysis

Saturated phospholipid DMPC, DPPC and cholesterol (100 µM) were dissolved in a centrifuge tube containing 10 µL chloroform and statically put it aside to dispel the solution. According to the references,^[^ ^54^ ^]^ then 2% Tween‐20 buffer with Ru(II) complexes (10 µM) was added to resuspend and stabilize DMPC, DPPC and cholesterol rather than form liposomes, and the turbidity alteration was recorded by digital camera.

### Lipid Phase Separation Monitoring

Saturated phospholipid DMPC (100 µM) mixed **Ru‐LipM** (10 µM) were dissolved in 35 mm confocal dishes containing 10 µL chloroform and statically put it aside to dispel the solution. Then 2% Tween‐20 buffer was added to resuspend DMPC/**Ru‐LipM** mixture and the dynamic liquid phase separation drop formation with fluorescence was recorded by Carl Zeiss LSM 880 laser scanning confocal microscope (Germany). **Ru‐LipM**: λ_ex_ = 488 nm, λ_em_ = 607 ± 20 nm.

### Interaction between Saturated Lipids and **Ru‐LipM** by IR Spectrum

Saturated lipids (DMPC, DPPC and cholesterol) and **Ru‐LipM** were mixed into different ratio in 10 µL chloroform and tableted. The IR spectra were recorded with ATR mode on PerkinElmer Frontier IR spectrometer (USA).

### Interaction between DMPC and **Ru‐LipM** by Raman Spectrum

Saturated phospholipid DMPC and **Ru‐LipM** were mixed into different ratio in 10 µL chloroform and statically put it aside to dispel the solution. The Raman spectra were recorded on Renishaw inVia confocal Raman Spectroscopy (UK).

### Cell Cultures

Cervical cancer (HeLa and U14), small cell lung cancer (A549 and LLC), breast cancer (MDA‐231 and 4T1) and normal (lung fibroblast (HLF) and mammary epithelial (MCF‐10A)) cell lines were maintained in DMEM or RPMI 1640 medium supplemented with 10% FBS and 1% penicillin/streptomycin at 37 °C in an atmosphere with 5% CO_2_.

### Cytotoxicity In Vitro

Cytotoxicities of Ru(II) complexes against above cell lines were evaluated using MTT assays performed as reference.^[^ ^8b^ ^]^ Briefly, cells were seeded in 96 well plates and treated with various concentrations of Ru(II) complexes for 72 h, and then incubated with MTT for 4 h. For phototoxicity, cells were incubated with Ru(II) complexes for 12 h and irradiated with a 450 nm LED light array in a low irradiation dose (17 mW cm^−2^, 2 J cm^−2^) or a normal irradiation dose (17 mW cm^−2^, 15 J cm^−2^). After another 60 h of incubation, cell viabilities of different groups were determined by MTT assay. Necrosulfonamide (10 µM), ferrostatin‐1 (10 µM), disulfiram (4 µM), z‐VAD‐fmk (40 µM), 3‐methyladenine (500 µM) and necrostatin‐1 (50 µM) added 1 h before irradiation were used for necroptotic inhibition, ferroptotic inhibition, pyroptotic inhibition, apoptotic inhibition, autophagic inhibition and necrotic inhibition, respectively.

### Cellular Uptake and PLIM

To quantify the uptake of ruthenium contents, HeLa and HLF cells were incubated with **Ru‐LipM** and **Ru1**−**Ru3** (10 µM) for 6 or 12 h, and then analyzed by XSERIES 2 ICP‐MS. For imaging investigation, HeLa cells were incubated with **Ru‐LipM** (10 µM) at 37 °C for different time intervals and visualized by confocal microscopy with a two‐photon laser. **Ru‐LipM**: λ_ex_ = 488 nm/810 nm, λ_em_ = 607 ± 20 nm.

### Colocalization Assay

HeLa cells were incubated with **Ru‐LipM** (10 µM) and then stained with LTDR (200 nM, 30 min), LD‐TDR (1×, 30 min), Peroxiasome‐RFP (2 µg, transfection for 12 h), MTDR (150 nM, 30 min) and ERTR (1×, 30 min) for different time intervals at 37 °C. After washed three times with PBS, cells were visualized by confocal microscopy immediately. **Ru‐LipM**: λ_ex_ = 488 nm, λ_em_ = 607 ± 20 nm. LTDR: λ_ex_ = 633 nm, λ_em_ = 668 ± 20 nm. LD‐TDR: λ_ex_ = 633 nm, λ_em_ = 660 ± 20 nm. Peroxiasome‐RFP: λ_ex_ = 561 nm, λ_em_ = 599 ± 20 nm. MTDR: λ_ex_ = 633 nm, λ_em_ = 665 ± 20 nm. ERTR: λ_ex_ = 561 nm; λ_em_ = 597 ± 20 nm.

### Cellular Localization Measured by ICP‐MS

HeLa cells were incubated with **Ru‐LipM** (10 µM) for 6 or 12 h. The organelle (cytoplasm, nucleus, mitochondrion, membrane, lysosome and ER) proteins were extracted according to the organelle extraction kits as listed in *Materials and methods*. Subsequently, proteins were quantified by BCA protein quantification kit (Biotime, China). The collected proteins were digested in 200 µL HNO_3_ at 60 °C for 1 h and diluted to 5 mL with 10 ppb In as the interior label. The concentration of Ru was measured using the XSERIES 2 ICP‐MS. The error bars represent the standard deviation (n = 3).

### Cellular Phospholipid Phase Separation

HeLa cells were incubated with DSPE‐PEG‐FITC (10 µM) for 24 h at 37 °C and then treated with **Ru‐LipM** (10 µM) for another 12 h. After washed three times with PBS, cells were visualized by confocal microscopy immediately. **Ru‐LipM**: λ_ex_ = 488 nm, λ_em_ = 607 ± 20 nm. DSPE‐PEG‐FITC: λ_ex_ = 488 nm, λ_em_ = 525 ± 20 nm.

### Elemental Mapping by Hyperspectral Imaging System

Hela cells were seeded in slides and incubated with **Ru‐LipM** (10 µM) for 6 or 12 h. After that, these cells were washed three times with pre‐cooled PBS and fixed with 4% paraformaldehyde solution. Cellular hyperspectral imaging was measured by a CytoViva Nano Scale Hyperspectral Microscope Imaging System (HIS, CytoViva, USA), capturing the visible and near‐infrared spectrum from 400 to 1000 nm. Hyperspectral images were acquired using a collection time of 0.25 s and a white light source with a 20 × or 60 × oil immersion lens. The scattering spectra were analyzed by the method of Spectral Angle Mapper using the CytoViva Analysis Tool of ENVI software (version 4.8).

### Neutral Lipid Sphere Formation

Hela cells were seeded in 35 mm confocal dishes and incubated with **Ru‐LipM** (10 µM) for 6, 8 and 12 h to induce co‐assembly of complex with cellular lipid. After that, these cells were washed three times with pre‐cooled PBS and fixed with 4% paraformaldehyde solution. Then cells were infiltrated with 60% isopropanol for 2 min and stained with oil red O working solution for 5 min. After removing excess dye, samples were counterstained by hematoxylin for 30 s and differentiated hydrochloric acid alcohol. Finally, cells were washed three times with cold deionized water and imaged using Carl Zeiss Primovert fluorescent inverted microscope (Germany).

### Intracellular TG Detection

Hela cells were seeded in 6 cm dishes and incubated with **Ru‐LipM** (10 µM) for 6, 8 and 12 h. Then these cells were washed three times with pre‐cooled PBS and sonicated. After centrifuging, supernatants were operated according to the instruction of General TGC/Triglyceride ELISA Kit (EIAab, China).

### TEM

HeLa cells were seeded in 10 cm culture dishes and treated with **Ru‐LipM** (10 µM) for 12 h. For laser‐irradiated samples, **Ru‐LipM**‐treated cells were further irradiated for 2 min (450 nm, 17 mW cm^−2^, 2 J cm^−2^) and continuously incubated for 1 or 2 h. These cells were trypsinized, washed and fixed with 2.5% glutaric dialdehyde at 4 °C overnight. After that, samples were dehydrated, cured, embedded, sliced and stained with Osmium tetroxide. For non‐negative staining samples, osmium tetroxide fixing and uranyl acetate/lead citrate staining procedures were skipped over. All samples were observed using TEM (Hitachi, Japan).

### Lipidomic Profiling

HeLa cells were seeded in 15 cm culture dishes and treated with **Ru‐LipM** (10 µM) for 12 h. For laser‐irradiated samples, **Ru‐LipM**‐treated cells were further irradiated for 2 min (450 nm, 17 mW cm^−2^, 2 J cm^−2^) and continuously incubated for 2 h. The sample preparation and data acquirement were performed as reference.^[^ ^55^ ^]^ Briefly, These cells were harvested by scrapers, and a mixture of 1.2 mL water/methanol/methyltertbutylether (v/v/v = 1/1/4) was added to each sample. After sonicated in an ice bath for 20 min and placed at room temperature for 30 min, samples were centrifuged at 10 °C at 14 000 g for 15 min. Supernatant was collected and dried by nitrogen, and then 200 µL isopropyl alcohol/acetonitrile solution (v/v = 9/1) was added to the samples before MS analysis. The qualified data were identified aligned, de‐noised and extracted metabolite characteristic peaks from the original data using LipidSearch 4.1 software. The detected lipid metabolites were qualitatively identified through comparison with LipidSearch database. Statistical analysis (T test/principal component analysis (PCA)/OPLS‐DA) was conducted according to the specific requirements for sample grouping and comparison.

### Western Blotting

HeLa cells were seeded in 15 cm culture dishes and treated with **Ru‐LipM** (10 µM) for 6 or 12 h. For laser‐irradiated samples, cells were treated with **Ru‐LipM** (10 µM) for 12 h in dark, furtherly irradiated for 2 min (450 nm, 17 mW cm^−2^, 2 J cm^−2^) and continuously incubated for 1 or 2 h. Cells were harvested and lysed by IP lysis buffer (ThermoFisher, USA). The protein concentration was quantified by BCA protein quantification kit (Biotime, China). Equal amounts of cellular total proteins were separated on SDS‐PAGE and then transferred onto polyvinylidene difluoride membranes (Roche, USA). Membranes were blocked in QuickBlock blocking buffer (Biotime, China) and incubated overnight at 4 °C with primary antibodies. After a subsequent washing step, the membrane was incubated with appropriate HRP‐conjugated secondary antibody. Images were captured using a Tanon‐4160SF imaging station (China) and analyzed with ImageJ software (Version 1.38).

### Intracellular Distribution of Fe^2+^ Ions

HeLa cells were seeded in 35 mm confocal dishes and treated with **Ru‐LipM** (10 µM) for 12 h in dark, subsequently irradiated for 2 min (450 nm, 17 mW cm^−2^, 2 J cm^−2^) and continuously incubated for 1 or 2 h. They were then co‐incubated with FerroOrange (1 µM) and LTDR (200 nM) for 30 min. These cells were observed using confocal microscopy. **Ru‐LipM**: λ_ex_ = 488 nm, λ_em_ = 607 ± 20 nm. LTDR: λ_ex_ = 633 nm, λ_em_ = 668 ± 20 nm. FerroOrange: λ_ex_ = 561 nm, λ_em_ = 595 ± 20 nm.

### Cellular ROS Detection

HeLa cells were seeded in 35 mm confocal dishes and treated with **Ru‐LipM** (10 µM) for 12 h and subsequently irradiated for 2 min (450 nm, 17 mW cm^−2^, 2 J cm^−2^). Cells were washed twice and incubated with 10 µM DCFH‐DA for 15 min at 37 °C in the dark. For scavenging experiment, cells were preincubated with ROS scavengers (NaN_3_:5 mM, Ebselen: 50 µM) for 2 h. These cells were observed using confocal microscopy. DCF: λ_ex_ = 488 nm, λ_em_ = 525 ± 20 nm.

### Measurement of MDA Levels, NADP^+^/NADPH and GSSH/GSH Ratios

HeLa cells were seeded in 6‐well plates and treated with **Ru‐LipM** (10 µM) for 6 or 12 h. For laser‐irradiated samples, cells were treated with **Ru‐LipM** (10 µM) for 12 h in dark, furtherly irradiated for 2 min (450 nm, 17 mW cm^−2^, 2 J cm^−2^) and continuously incubated for 1 or 2 h. The MDA levels, NADP^+^/NADPH and GSSH/GSH ratios were measured using the Lipid Peroxidation MDA Assay Kit (Beyotime, China), NADP^+^/NADPH Assay Kit with WST‐8 (Beyotime, China) and GSSG/GSH Assay Kit (Beyotime, China) following these manual protocols, respectively.

### Determination of Extracellular ATP Levels

HeLa cells were seeded in 6‐well plates and treated with **Ru‐LipM** (10 µM) for 6 or 12 h. For laser‐irradiated samples, cells were treated with **Ru‐LipM** (10 µM) for 12 h in dark, furtherly irradiated for 2 min (450 nm, 17 mW cm^−2^, 2 J cm^−2^) and continuously incubated for 1 or 2 h. The extracellular ATP concentration was measured by luciferase luminescence assay using an ATP quantification Assay Kit (Beyotime, China).

### Intracellular HMGB‐1/etco‐CRT Levels

HeLa cells were seeded in 6‐well plates and treated with **Ru‐LipM** (10 µM) for 6 or 12 h. For laser‐irradiated samples, cells were treated with **Ru‐LipM** (10 µM) for 12 h in dark, furtherly irradiated for 2 min (450 nm, 17 mW cm^−2^, 2 J cm^−2^) and continuously incubated for 1 or 2 h. The samples were collected according to the reference.^[^ ^56^ ^]^

### RNA‐Seq and Bioinformatics

The experiment was carried out as described before by the Biomarker Technologies (China).^[^ ^57^ ^]^ HeLa cells were seeded in 15 cm culture dishes and treated with **Ru‐LipM** (10 µM) for 12 h. For laser‐irradiated samples, **Ru‐LipM**‐treated cells were irradiated for 2 min (450 nm, 17 mW cm^−2^, 2 J cm^−2^) and continuously incubated for 2 h. Total RNA was extracted by Trizol reagent and purified using RNeasy mini kit (Qiagen, Germany). The integrity and concentration of RNA was assessed using the RNA Nano 6000 Assay Kit (Agilent Technologies, CA, USA). Sequencing libraries were generated using NEBNext Ultra RNA Library Prep Kit for Illumina (#E7530L, NEB, USA). RNA concentration of library was measured using Qubit RNA Assay Kit in Qubit 3.0 and then diluted to 1 ng µL^−1^. Insert size was assessed using the Agilent Bioanalyzer 2100 system (Agilent Technologies, USA), and qualified insert size was accurately measured by the StepOnePlus Real‐Time PCR System. The libraries were sequenced on an Illumina platform to generate 150 bp paired‐end reads, which were mapped to reference human genome sequence hg38 (ftp://ftp.ensembl.org/pub/release‐95/fasta/homo_sapiens/dna/) assembled by HISAT (Version 2.0) software. FDR of < 0.05 and > 200 bp were considered as differentially expressed. GSEA was performed following standard procedure (http://www.broadinstitute.org/gsea/doc/GSEAUserGuideFrame.html).

### M2‐Macrophage Culture

Mouse monocytes were isolated from the myeloid of mice using a literature method.^[^ ^58^ ^]^ Briefly, the monocytes were separated from mouse femurs and tibias, and cultured with M‐CSF (50 ng mL^−1^, R&D, USA) for 7 days to form M0‐macrophages. Subsequently, IL‐4 (20 ng mL^−1^, Pepreotech, USA) or IL‐10 (20 ng mL^−1^, Pepreotech, USA) were added to transform M0‐ into M2‐macrophages.

### RT‐qPCR Measurement

The expression of genes was measured by RT‐qPCR as previously described using a Roche LightCycler 480 Detection System (Roche, USA) and SYBR Green I Master (Roche, USA).^[^ ^8b^ ^]^ Briefly, HeLa or U14 cells were seeded in 6‐well plates and treated with **Ru‐LipM** (10 µM) for 6 or 12 h. For laser‐irradiated samples, cells were treated with **Ru‐LipM** (10 µM) for 12 h in dark, furtherly irradiated for 2 min (450 nm, 17 mW cm^−2^, 2 J cm^−2^) and continuously incubated for 1 or 2 h. Total RNA was isolated using the Trizol reagent (Sigma–Aldrich, USA). For M2‐macrophage samples, the **Ru‐LipM**‐treated U14 supernatants were added and incubated for another 12 h. The synthesis of cDNA was done using the Transcriptor First Strand cDNA Synthesis Kit (Roche, USA). GAPDH and β‐actin were used as housekeeping genes. The primer sequences were listed in Table S7 (Supporting Information).

### In Vivo Antitumor Evaluation

Pathogen‐free female BALB/c mice, 4–5 weeks of age, were purchased from GemPharmatech CoLtd, China. The primary tumors were established by subcutaneous injection of U14 cells (1×10^7^) into the right axilla. 6 days later, the distant tumors were established by subcutaneous injection of U14 cells (1×10^7^) into the left axilla. After 2 days, the mice were randomly divided into four groups (5 mice per group), and **Ru‐LipM** (5.0 mg kg^−1^ in solubilizer, DMSO/PEG400/Tween80/saline, 5%, 20%, 5%, 70%, v/v/v/v) or Control (solubilizer) was intratumorally injected at the sites of primary tumors. For laser‐irradiated groups, mice were irradiated with a 450 nm laser for 2 min (17 mW cm^−2^, 2 J cm^−2^). Volumes of the both primary and distant tumors were monitored by measuring the perpendicular diameter of the tumors using calipers every two days and calculated according to the formula:

(1) $$\mathrm{Tumor}\;\;\mathrm{volume}\;\;\left(V\right)\;=\;\frac{\left(\mathrm{Tumor}\;\;\mathrm{length}\right)\times {\left(\mathrm{tumor}\;\;\mathrm{width}\right)}^{2}}{2}$$

For hematoxylin‐eosin (H&E) staining, tumor samples and organs were collected and fixed with 4% paraformaldehyde at 4 °C. The samples then were transferred to 10% formalin neutral buffer and embedded in paraffin, stained by H&E and observed under an inverted fluorescence microscope (Zeiss, Germany).

### In Vivo Antitumor Immunity

Tumors (n = 5 mice in each group) were made into a single cell suspension using collagenase IV (1 mg mL^−1^, Worthington, USA) and DNase I (1 µg mL^−1^, ThermoFisher, USA) at 37 °C with agitation in DMEM with 10% FBS. After being filtered through a 70 µm nylon cell strainer, red blood cells were removed by lysis buffer. Cells were stained with CD11b‐BV421, CD206‐FITC, F4/80‐APC, CD11c‐FITC, CD80‐APC, CD86‐PE, CD8a‐APC, CD4‐BV421, CD3‐FITC or Foxp3‐PE and analyzed by flow cytometry. For intracellular analysis, cells were pretreated with Intracellular Fixation and Permeabilization kit (eBioscience, USA). Serums were isolated from mice and stored in liquid nitrogen for analysis. Secretions of IFN‐γ, TNF‐α, IL‐1β, IL‐6 and IL‐12p70 were measured by ELISA kit according to the manufacturer's instructions purchased from Elabscience (China).

### Ethics Statements for Animal Experiments

All of the animal experiments were approved by the Institutional Animal Care and Use Committee of Sun Yat‐Sen University. The assigned Animal Ethics Approval number was 2022001489. All animal operations were in accord with the guidelines of above institution and the approved experiment number was F2022‐0342XS.

### Statistical Analysis

Statistical analysis was performed with Prism 8.0 (Graph‐Pad Software). All results were shown as mean ± SD. All the experiments were conducted at least thrice with triplicates in each experiment. Statistical significance was analyzed using an unpaired, Student's two‐tailed *t*‐test (*p < 0.05, **p <0.01 and ***p <0.001).

## Conflict of Interest

The authors declare no conflict of interest.

## Supporting information



Supporting Information

Supplemental Movie 1

Supplemental Movie 2

Supporting Information

## Data Availability

The data that support the findings of this study are available in the supplementary material of this article.
