# Supplementary material for: Ruthenium(II) Lipid‐Mimics Drive Lipid Phase Separation to Arouse Autophagy‐Ferroptosis Cascade for Photoimmunotherapy
Source: Adv Sci (Weinh). 2024 Nov 22;12(3):2411629. doi: 10.1002/advs.202411629 (PMC11744722; doi:10.1002/advs.202411629)
Supplement: Supplementary file 1 — Supporting Information [file ADVS-12-2411629-s003.docx]

**Supporting Information**

**For**

**Ruthenium(II) Lipid-Mimics Drive Lipid Phase Separation to Arouse Autophagy-Ferroptosis Cascade for** **Photoimmunotherapy**

Yue Zheng,^+[a][b]^ Wen-Jin Wang,^+[a]^ Jing-Xin Chen,^[a]^ Kun Peng,^[a]^ Xiao-Xiao Chen,^[a]^ Qing-Hua Shen,^[a]^ Bing-Bing Liang,^[a]^ Prof. Zong-Wan Mao*^[a]^ and Prof. Cai-Ping Tan*^[a]^

^[a]^ MOE Key Laboratory of Bioinorganic and Synthetic Chemistry, State Key Laboratory of Anti-Infective Drug Development, IGCME, GBRCE for Functional Molecular Engineering, School of Chemistry, Sun Yat-Sen University, Guangzhou 510006, P. R. China

^[b]^ Guangdong Province Key Laboratory of Pharmaceutical Bioactive Substances, School of Bioscience and Biopharmaceutics, Guangdong Pharmaceutical University, Guangzhou 510006, P. R. China

E-mail: cesmzw@mail.sysu.edu.cn (M.Z.W.), tancaip@mail.sysu.edu.cn (T.C.P.)

**^+^** These authors contributed equally to this work.

**Table of Content**

[Materials and methods 3](#_Toc179983982)

[Supplementary figures 6](#_Toc179983983)

[Supplementary tables 38](#_Toc179983984)

[References 45](#_Toc179983985)

Materials and methods

All reagents were commercially available and used without further purification unless specifically noted. RuCl_3_•3H_2_O, 1,10-phenanthroline (phen) and dipyrido[3,2-a:2',3'-c]phenazine (dppz) were purchased from Adamas (China). 11,12-bis(nonyloxy)dipyrido[3,2-a:2',3'-c]phenazine (bpdppz), 11,12-bis(nonyloxy)dipyrido[3,2-a:2',3'-c]phenazine (bndppz) and 11,12-bis(dodecyloxy)dipyrido[3,2-a:2',3'-c]phenazine (bdodppz) were synthesized and purified by Alfa Chemical (China). 1,2-dimyristoyl-sn-glycero-3-phosphocholine (DMPC), 1,2-dihexadecanoyl-rac-glycero-3- phosphocholine (DPPC) and cholesterol were purchased from Topscience Co. Ltd. (China). 1,2-distearoyl-sn-glycero-3-phosphorylethanolamine-polyethylene glycol-FITC (DSPE-PEG-FITC) was purchased from RuixiBio (China). Peroxiasome-RFP plasmid was synthesized by IgeBio Co. Ltd. (China). Methylene blue, 3-(4,5-dimethylthiazol-2-yl)-2,5-diphenyltetrazolium bromide (MTT), LysoTracker Deep Red (LTDR), MitoTracker Deep Red (MTDR), Endoplasmic Reticulum Tracker Red (ERTR), Prestained protein marker, Immunoprecipitation (IP) lysis buffer, DNase I and 2′,7′-dichlorodihydrofluorescein diacetate (DCFH-DA) were obtained from ThermoFisher Scientific (USA). 9,10-anthracenedipropionic acid (ABDA), 1,3-diphenylisobenzofuran (DPBF), Triton X-100, Oil Red O, hematoxylin, 4% paraformaldehyde solution, phosphate buffered saline (PBS), Tween 20, dimethyl sulfoxide (DMSO) and TRIzol Reagent were purchased from Sigma Aldrich (USA). Fetal bovine serum (FBS), trypsin, dulbecco's modified eagle medium (DMEM), roswell park memorial institute medium (RPMI 1640 Medium), penicillin-streptomycin (PS) were purchased from Gibco (USA). SYBR Green I Master kit and Transcriptor First Strand cDNA Synthesis Kit were purchased from Roche (USA). FerroOrange and Lipi-Deep Red (LD-TDR) were purchased from Dojindo Laboratories (Japan). Nucleoprotein Extraction kit, Mitochondria Isolation kit, Membrane Protein Extraction kit, sodium dodecyl sulfate-polyacrylamide gel electrophoresis (SDS-PAGE), sample loading buffer (5×), bicinchoninic acid assay (BCA) protein quantification kit, QuickBlock™ blocking buffer, oxidized nicotinamide adenine dinucleotide phosphate (NADP^+^)/NADPH assay kit with WST-8, glutathione (GSH) disulfide (GSSG)/GSH assay kit, adenosine triphosphate (ATP) quantification assay kit and lipid peroxidation malondialdehyde (MDA) assay kit were purchased from Biotime Biotechnology (China). Endoplasmic reticulum extraction kit and Lysosome extraction kit were purchased from Solarbio Life Science (China). 3-methyladenine, ferrostatin-1, necrosulfonamide, disulfiram, necrostatin-1 and z-VAD-fmk were purchased from MedChemExpress (USA). Interferon (IFN)-γ, tumor necrosis factor (TNF)-α, interleukin (IL)-1β, IL-6 and IL-12p70 enzyme-linked immunosorbent assay (ELISA) kits were purchased from Elabscience (China). General TGC/Triglyceride ELISA kit (EIAab, China), Macrophage colony-stimulating factor (M-CSF, R&D, USA), IL-4 (Pepreotech, USA), IL-10 (Pepreotech, USA), collagenase IV (Worthington, USA), Intracellular Fixation and Permeabilization kit (eBioscience, USA) and Amersham ECL Prime Western Blotting Detection Reagent (GE Healthcare, USA) were purchased from corresponding commercial sources. All the tested compounds were dissolved in DMSO as the stock solution, and diluted into the expected experiment concentration containing 1% (v/v) DMSO as needed. In each experiment, vehicle control (1% DMSO) was used as the reference group unless otherwise specified.

Microtubule-associated protein light chain 3 (LC3) rabbit monoclonal antibody (ab192890), SQSTM1/ sequestosome 1 (p62) rabbit monoclonal antibody (ab109012), autophagy-related gene 5 (ATG5) rabbit monoclonal antibody (ab108327), mechanistic target of rapamycin (mTOR) rabbit monoclonal antibody (ab134903), ferritin rabbit monoclonal antibody (ab75973), nuclear receptor coactivator-4 (NCOA4) rabbit monoclonal antibody (ab314553), glutathione peroxidase 4 (GPX4) rabbit monoclonal antibody (ab125066), acyl-CoA synthetase long-chain family member 4 (ACSL4) rabbit polyclonal antibody (22401-1-AP), NADPH oxidase 1 (NOX1) rabbit polyclonal antibody (17772-1-AP), NOX4 rabbit polyclonal antibody (14347-1-AP), B-cell lymphoma-2 (Bcl-2) mouse monoclonal antibody (YM3041), Bcl-2-associated X (BAX) rabbit polyclonal antibody (50599-2-Ig), high-mobility group box 1 protein (HMGB1) rabbit monoclonal antibody (ab79823), calreticulin (CRT) rabbit monoclonal antibody (ab92516), horse radish peroxidase (HRP)-conjugated anti-rabbit antibody (ab288151), donkey anti-rabbit IgG H&L (Alexa Fluor® 647) antibody (ab150075), HRP anti-GAPDH antibody (ab9482) and HRP anti-β-actin antibody (ab207674) were purchased from Abcam (UK). BV421 anti-mouse CD11b antibody (101235), FITC anti-mouse CD206 antibody (141704), APC anti-mouse F4/80 antibody (123116), FITC anti-mouse CD11c antibody (N418, Biolegend, 117305), PE anti-mouse CD86 antibody (105007), APC anti-mouse CD80 antibody (104713), FITC anti-mouse CD3 antibody (100203), BV421 anti-mouse CD4 (116023), APC anti-mouse CD8a antibody (100712) and PE anti-mouse Foxp3 antibody (126403) were obtained from Biolegend (USA).

^1^H NMR and ^13^C NMR were measured by a Bruker Avance 400 MHz, 500 MHz and 600 MHz spectrometer (Germany), and tetramethyl silane was used as the NMR reference internal standard. ESI-MS was tested on ThermoFinnigan LCQ Deca XP (USA), and the M/Z values cited represent the main peaks in the isotopic distribution. HR-ESI-MS was obtained using a Thermoscientific Q Exactive mass spectrometer (USA). Element analysis was determined by a Perkin-Elmer 240C analyzer (USA). UV-Vis absorption data were recorded on an Agilent Varian Cary 300 spectrophotometer (USA). Fluorescence data were recorded on Edinburgh FLS980 spectrophotometer (UK). MTT was determined by TECAN infinite M200 PRO multimode microplate reader (Switzerland). Flow cytometry was performed on BD FACSCalibur Flow Cytometer (UK). Confocal imaging and phosphorescence lifetime imaging (PLIM) were carried on Carl Zeiss LSM 880 laser scanning confocal microscope (Germany). Cell imaging was carried on a Primovert fluorescent Carl Zeiss fluorescent inverted microscope (Germany). Infrared (IR) spectra were recorded on a PerkinElmer Frontier IR spectrometer (USA). Raman spectra were recorded on Renishaw inVia^TM^ confocal Raman Spectroscopy (UK). The contents of ruthenium in cells were detected by Thermo Scientific XSERIES 2 inductively coupled plasma mass spectrometry (ICP-MS, USA). Lipidomics analysis was carried on Thermo Scientific™ Q Exactive™ Hybrid Quadrupole-Orbitrap™ Mass Spectrometer (USA). TEM images were recorded in Hitachi H7650 transmission electron microscope (80 kV, Japan). Hyperspectral imaging was carried out using a CytoViva dark-field hyperspectral imaging system (CytoViva, USA) mounted on an Olympus optical microscope. Western blot experiments were conducted on BIORAD Power Pac^TM^ HC Mini-Protean Tetra System (Singapore) and visualized by Tanon-4160SF (China). Polymerase chain reaction (PCR) was performed using a Roche LightCycler 480 Detection System (Roche, USA).

Supplementary figures

**Scheme S1** The synthetic routine of Ru(II) lipid-mimics.

**Figure S1** ESI-MS spectrum of bpdppz.

**Figure S2** ESI-MS spectrum of bndppz.

**Figure S3** ESI-MS spectrum of bdodppz.

**Figure S4** ESI-MS spectrum of **Ru1**.

**Figure S5** ESI-MS spectrum of **Ru2**.

**Figure S6** ESI-MS spectrum of **Ru3**.

**Figure S7** ESI-MS spectrum of **Ru-LipM**.


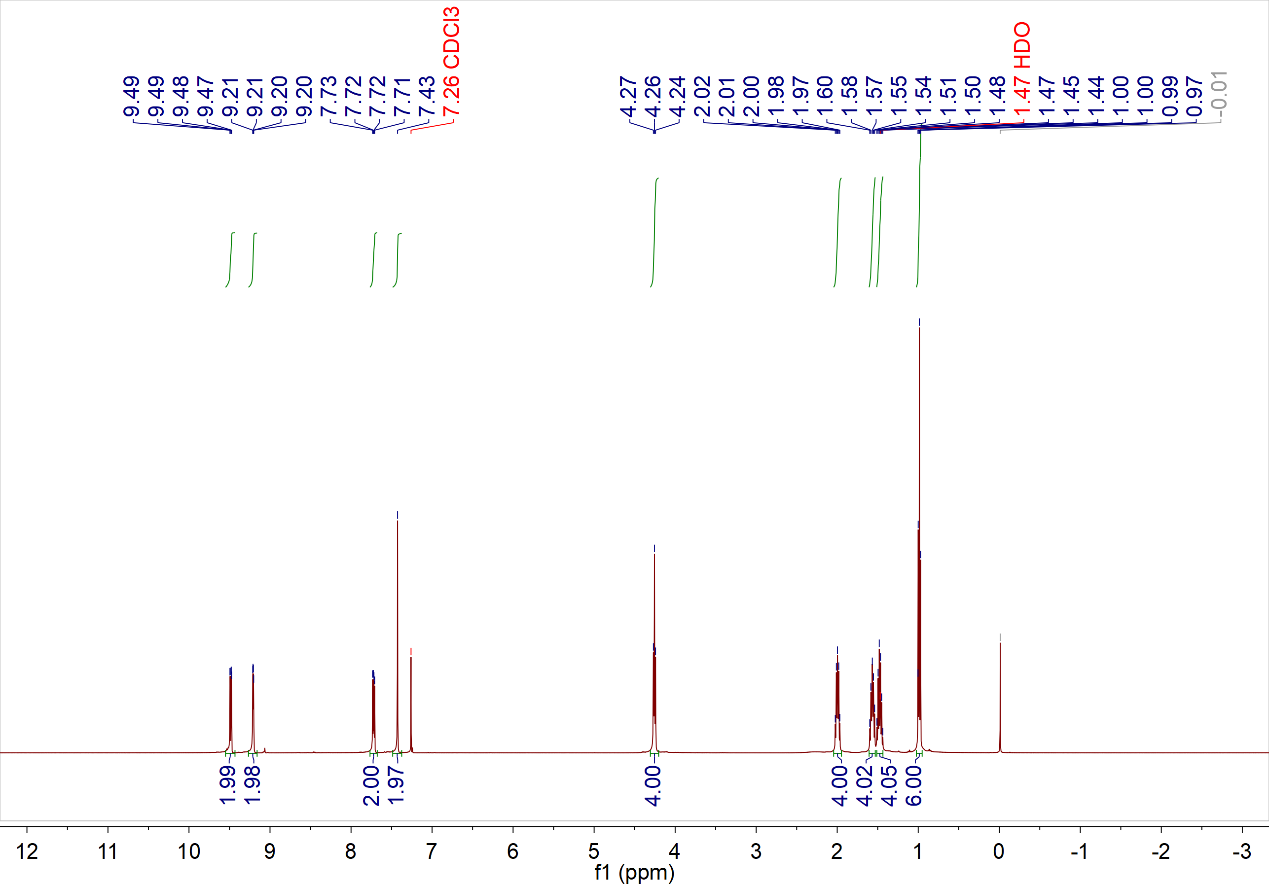


**Figure S8** ^1^H NMR spectrum of bpdppz.


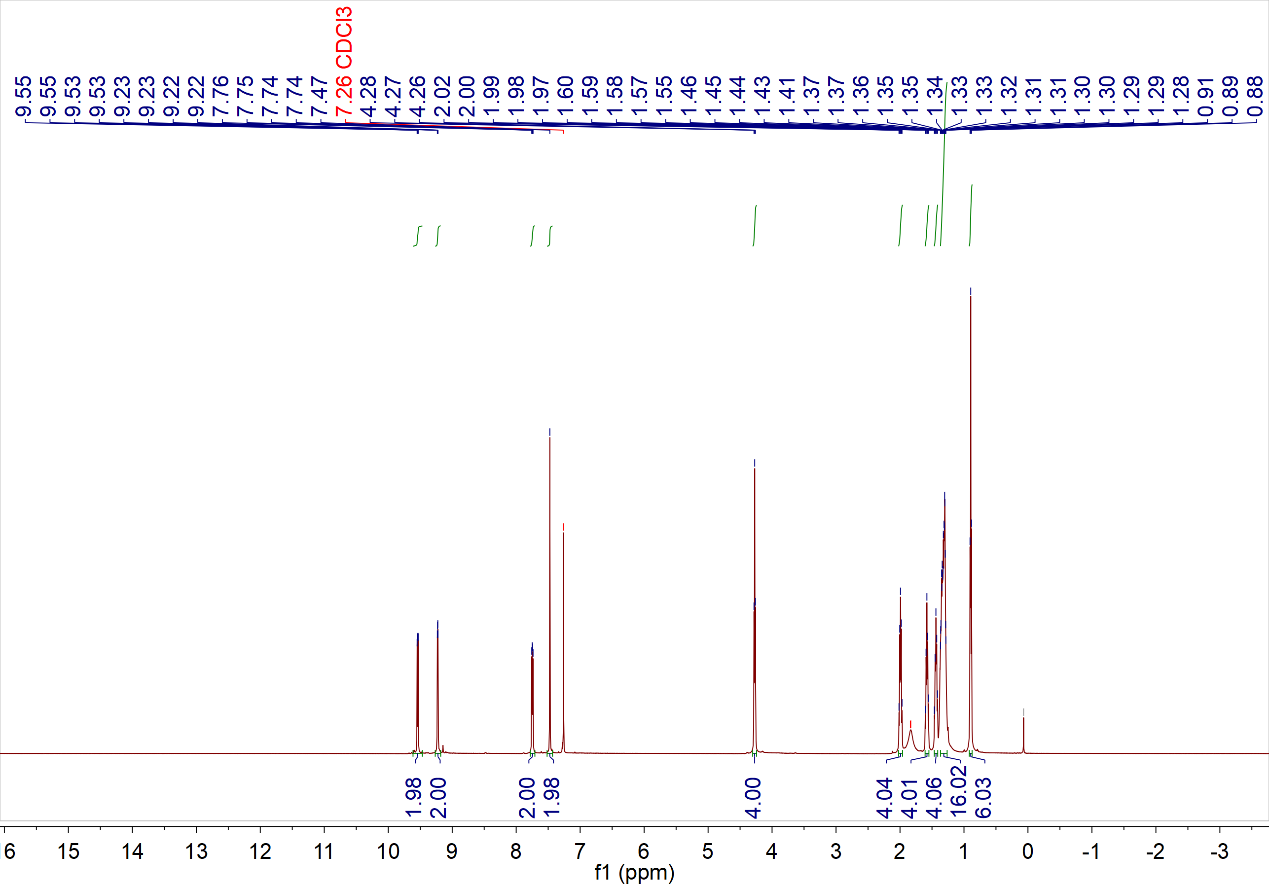


**Figure S9** ^1^H NMR spectrum of bndppz.


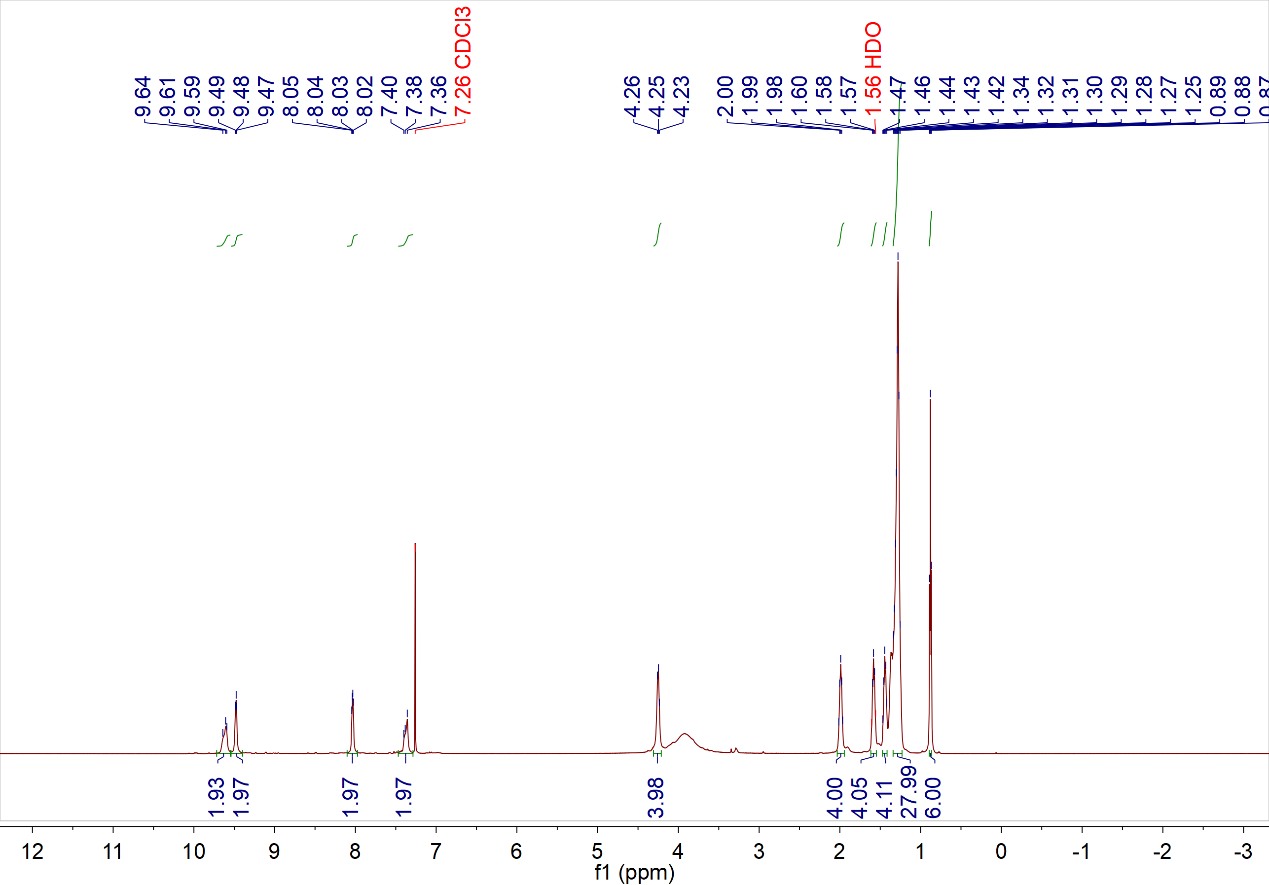


**Figure S10** ^1^H NMR spectrum of bdodppz.


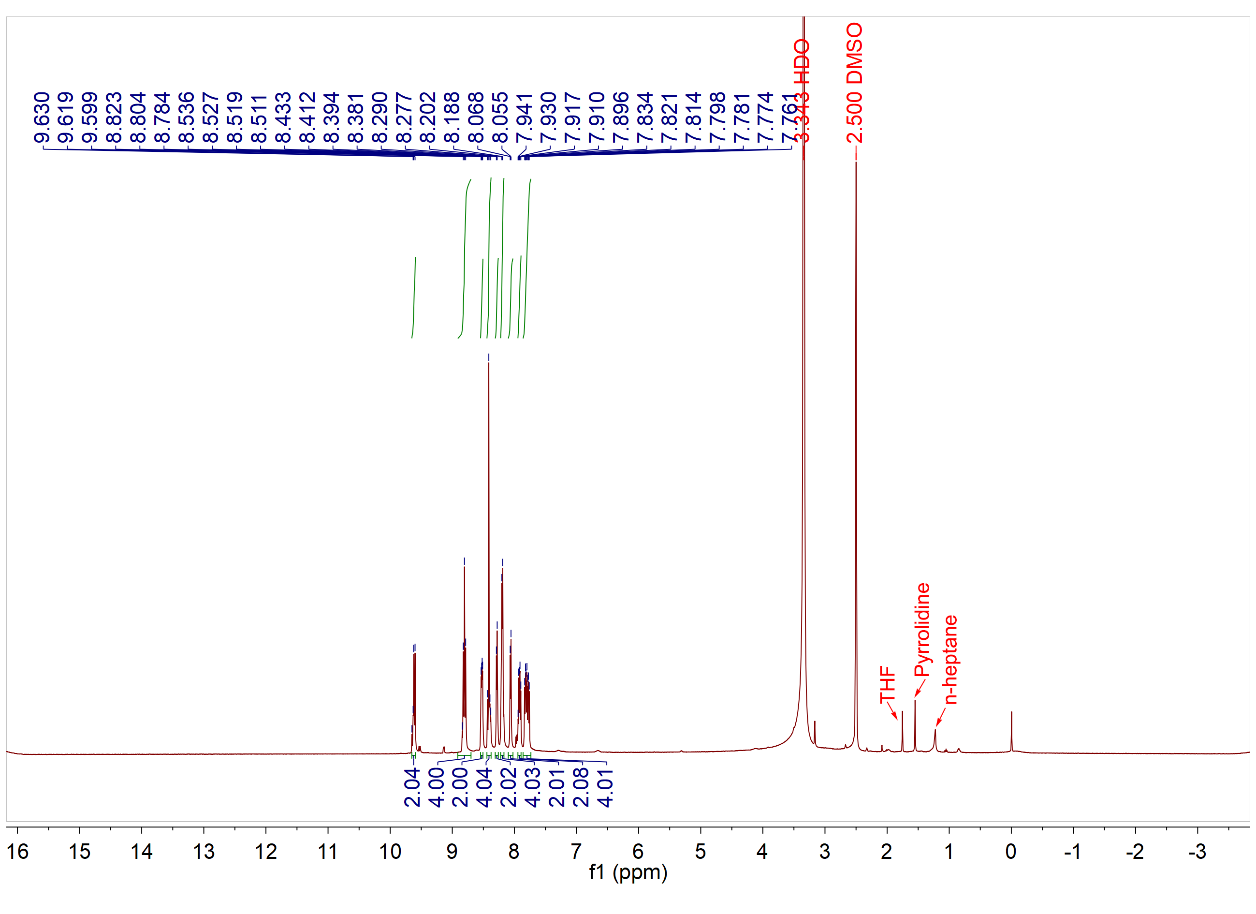


**Figure S11** ^1^H NMR spectrum of **Ru1**.


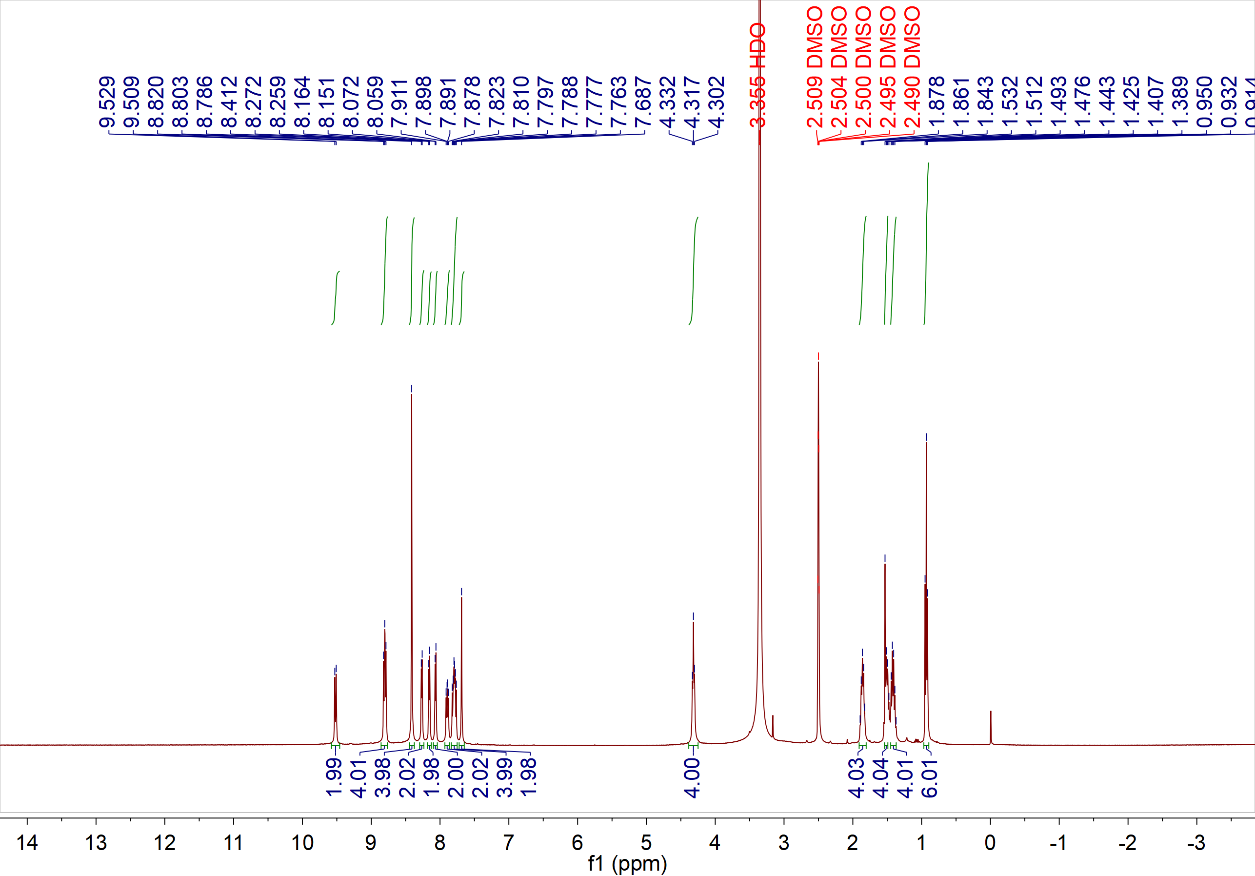


**Figure S12** ^1^H NMR spectrum of **Ru2**.


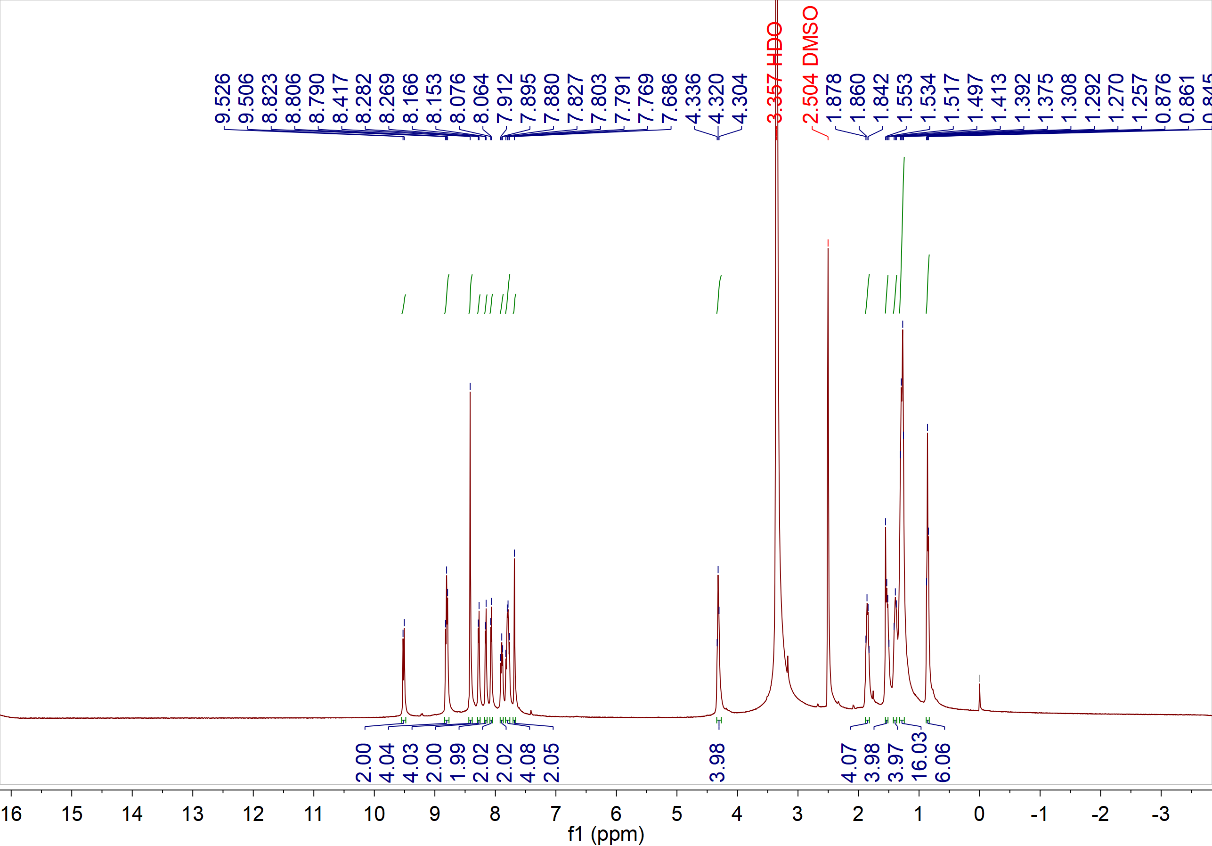


**Figure S13** ^1^H NMR spectrum of **Ru3**.


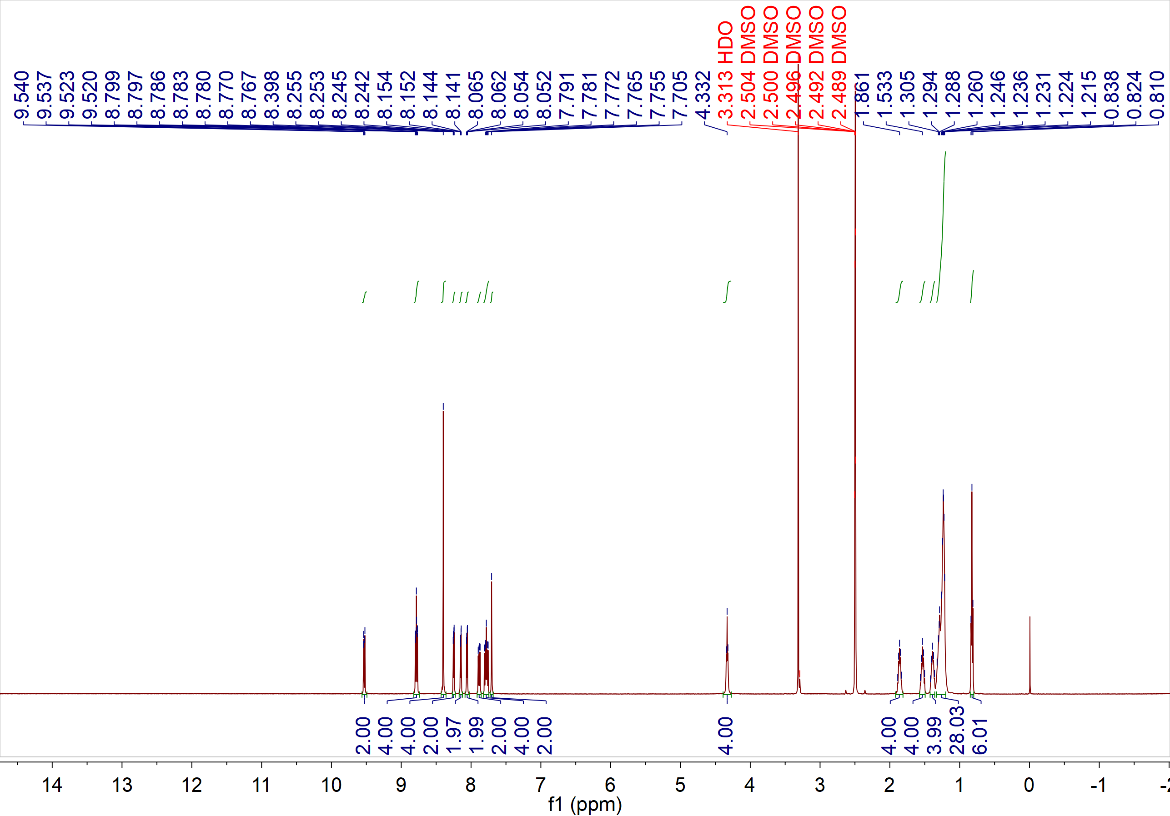


**Figure S14** ^1^H NMR spectrum of **Ru-LipM**.


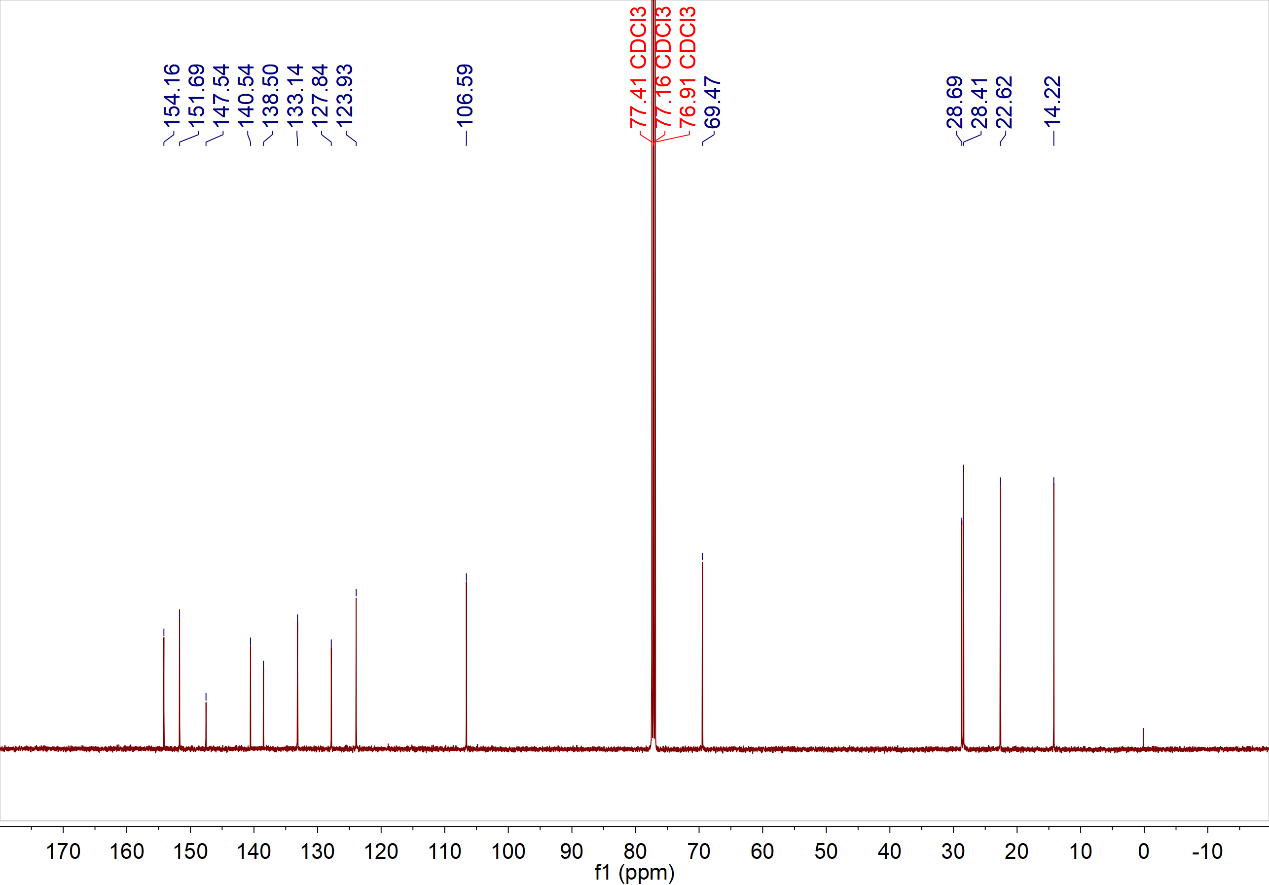


**Figure S15** ^13^C NMR spectrum of bpdppz.


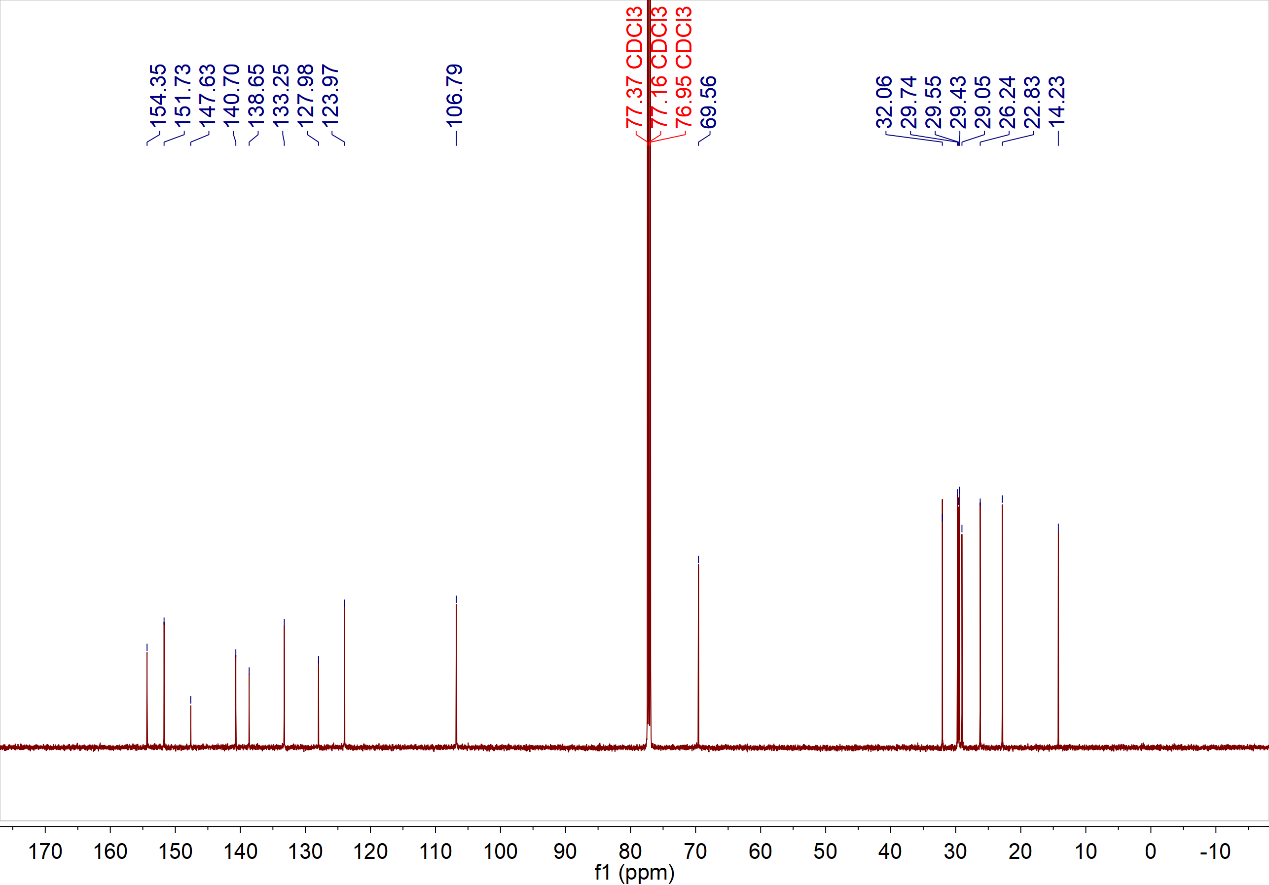


**Figure S16** ^13^C NMR spectrum of bndppz.


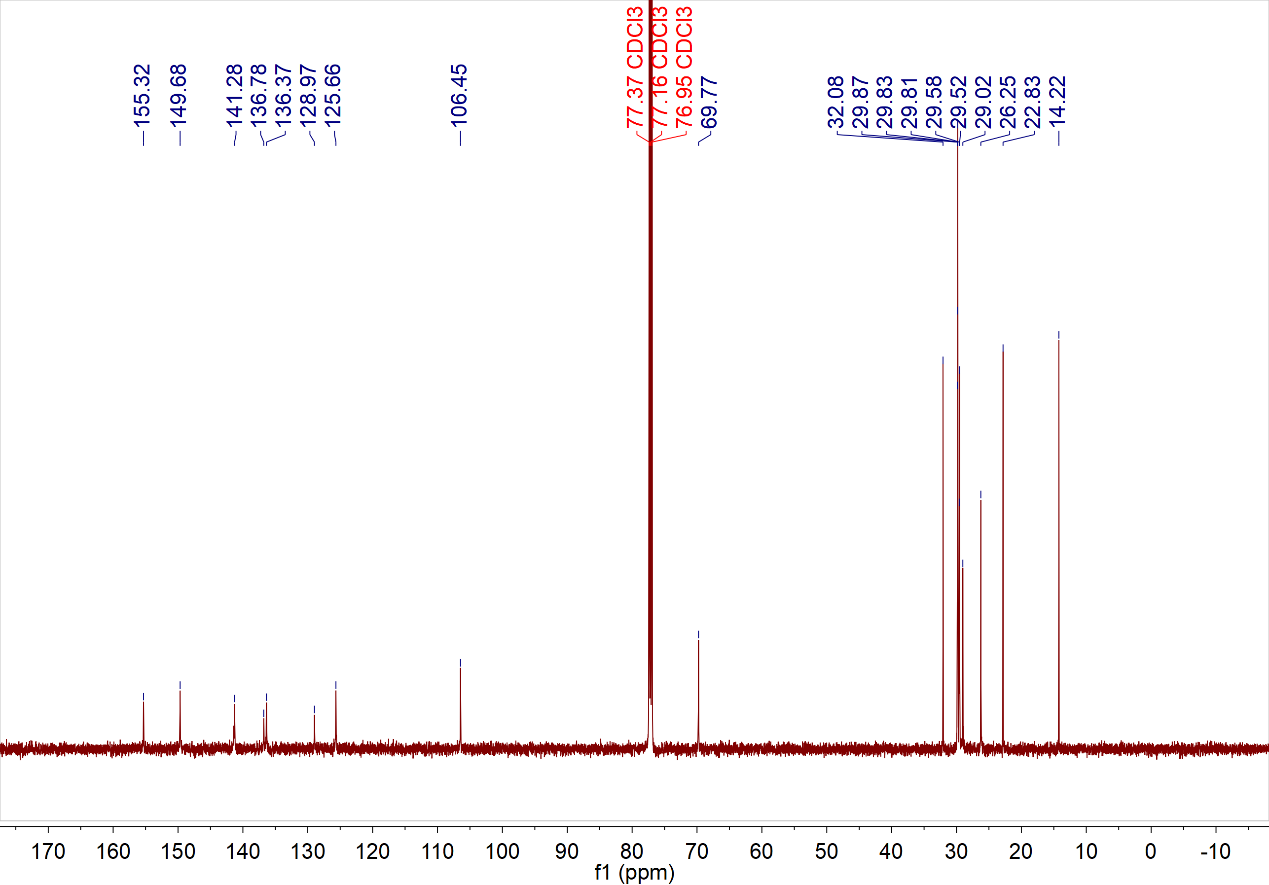


**Figure S17** ^13^C NMR spectrum of bdodppz.


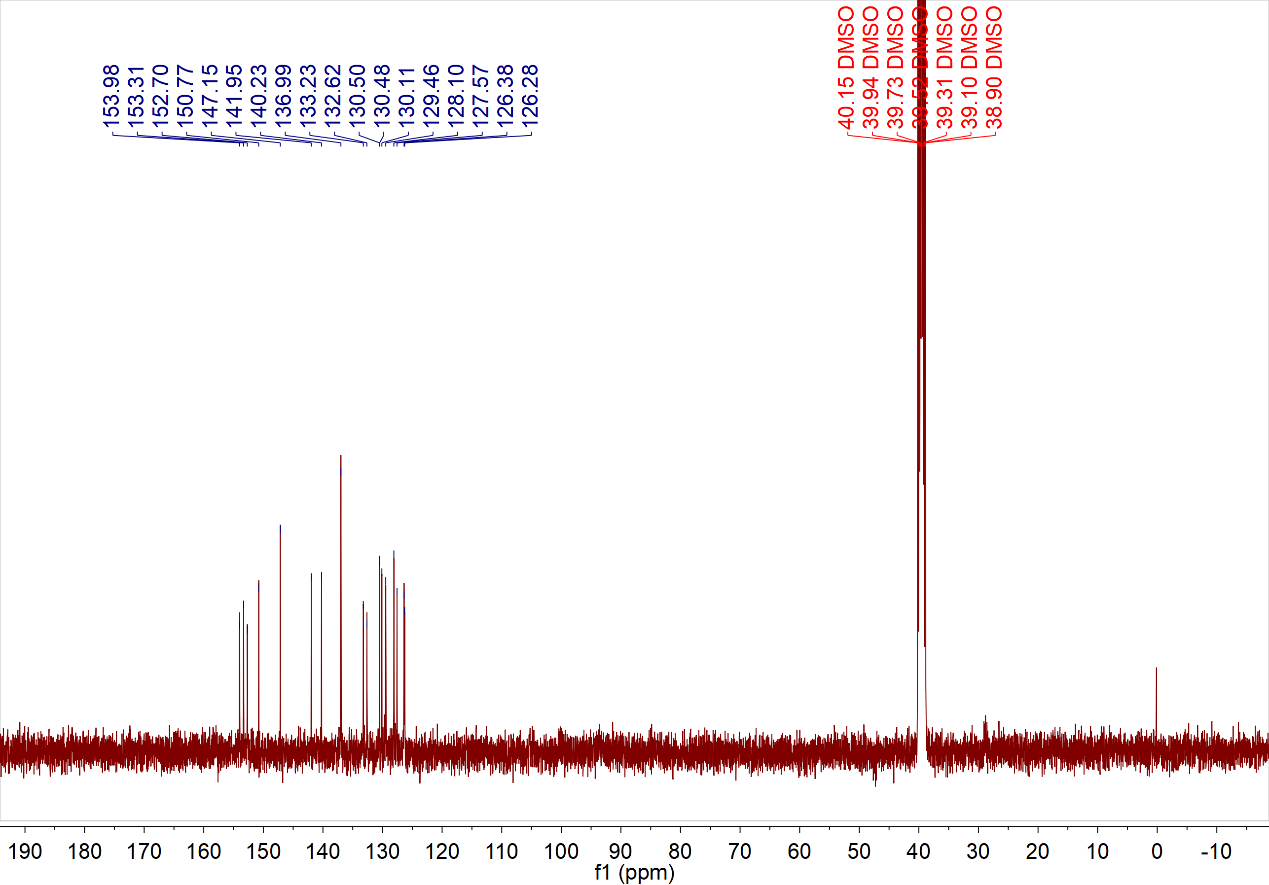


**Figure S18** ^13^C NMR spectrum of **Ru1**.


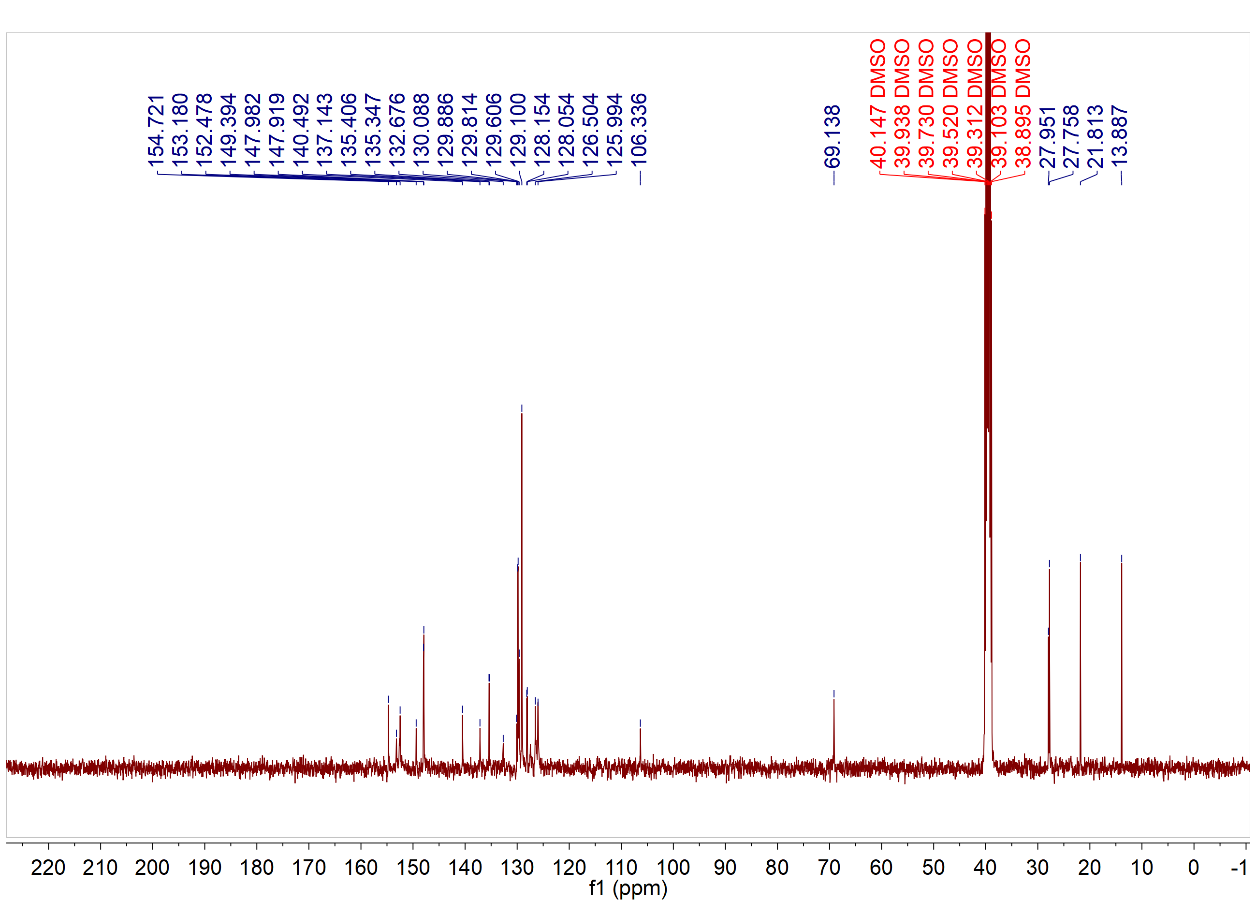


**Figure S19** ^13^C NMR spectrum of **Ru2**.


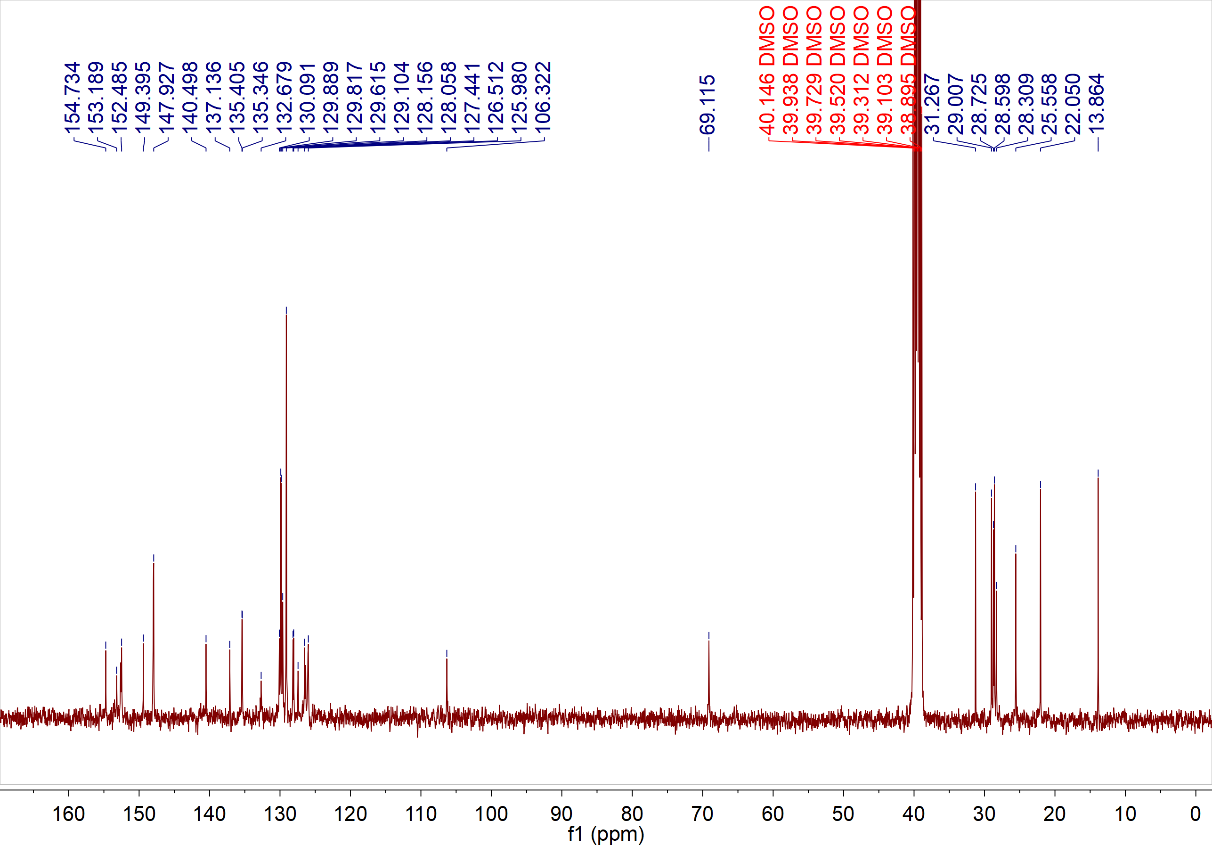


**Figure S20** ^13^C NMR spectrum of **Ru3**.


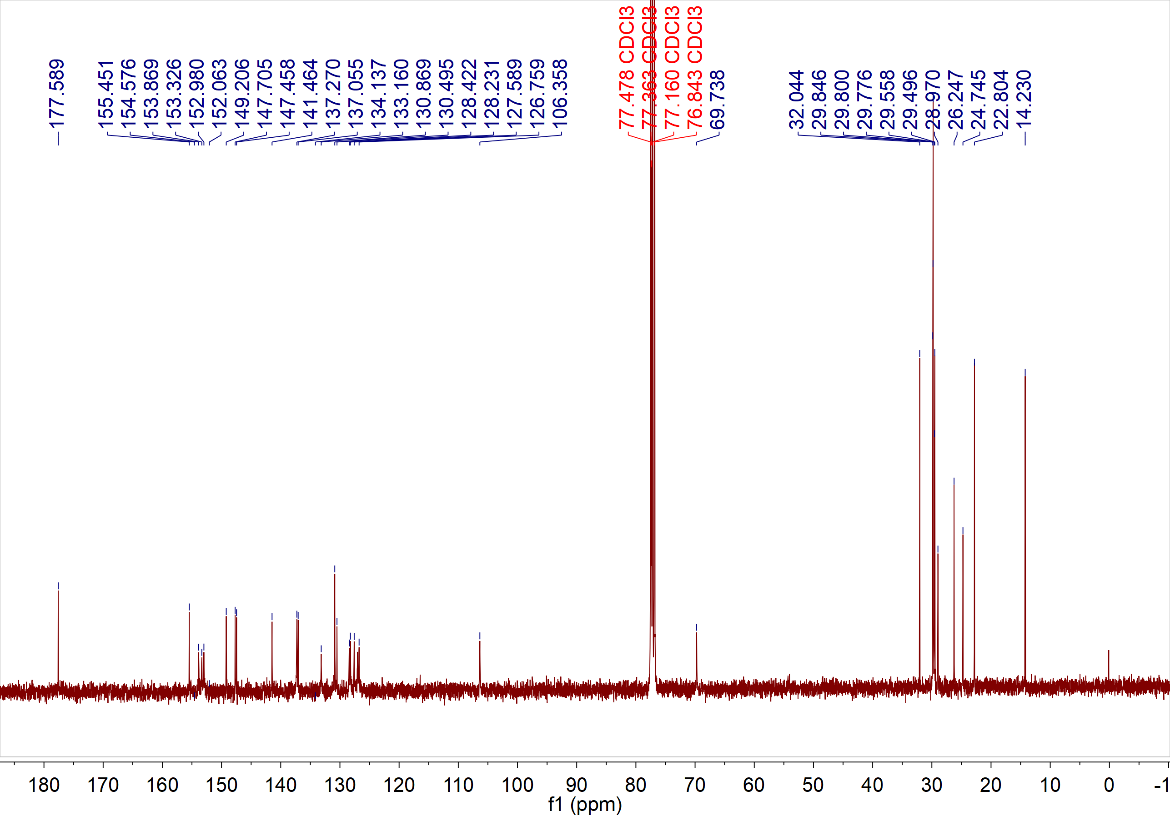


**Figure S21** ^13^C NMR spectrum of **Ru-LipM**.


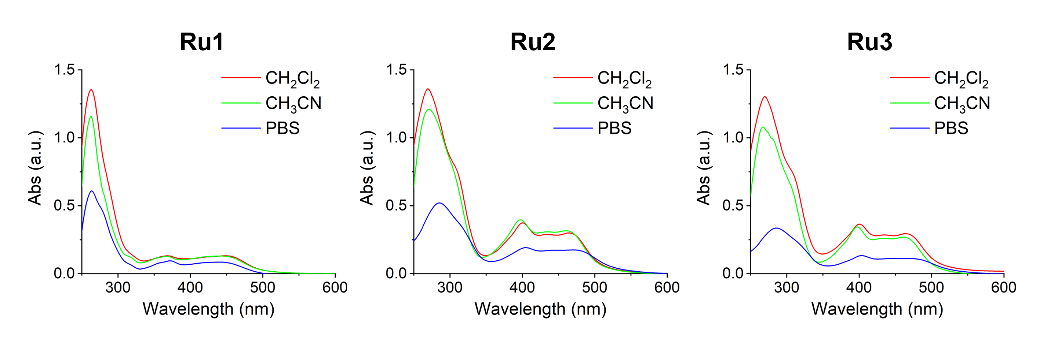


**Figure S22** UV-Vis absorption spectra of **Ru1**–**Ru3** (10 μM) in degassed CH_2_Cl_2_, CH_3_CN and PBS at 298 K.


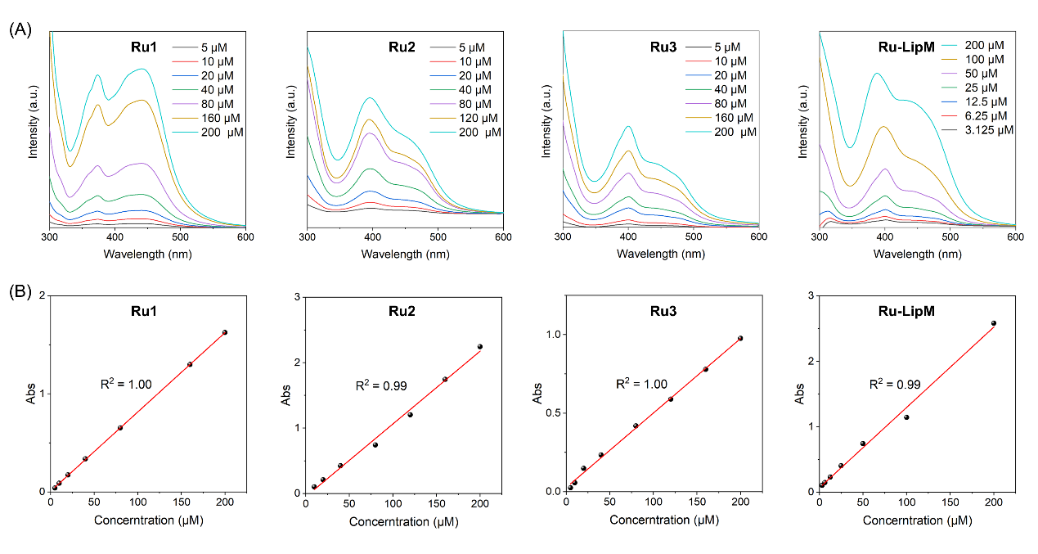


**Figure S23** (A) UV-Vis absorption spectra of **Ru1**−**Ru3** and **Ru-LipM** at the indicated concentrations. (B) Fitting curves of the absorbance of **Ru1**–**Ru3** and **Ru-LipM** at 442 nm, 470 nm, 464 nm and 451 nm *vs* concentration, respectively. Ru(II) complexes were dissolved in PBS and 1% DMSO (v/v) was used for solubilization.


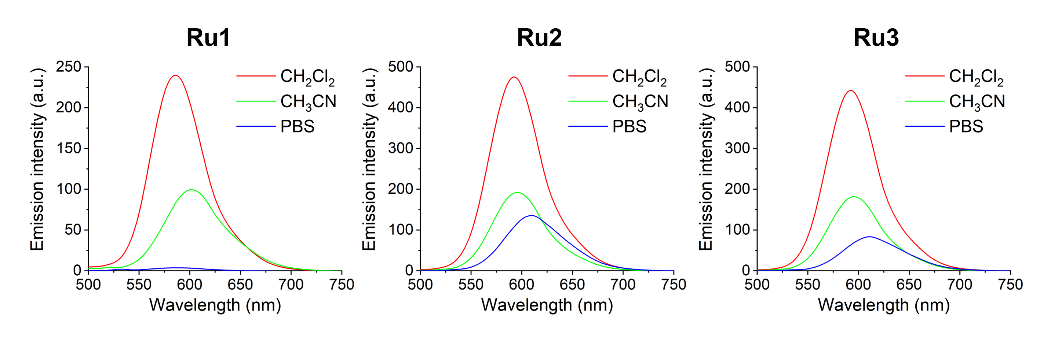


**Figure S24** Emission spectra of complexes **Ru1**–**Ru3** (10 μM) in degassed CH_2_Cl_2_, CH_3_CN and PBS at 298 K. The excitation wavelength is 450 nm.





**Figure S25** Emission spectra of complexes **Ru-LipM** (10 μM) in degassed toluene, n-butanol, isopropanol, chloroform, pyridine, acetone and methanol at 298 K. The excitation wavelength is 450 nm.


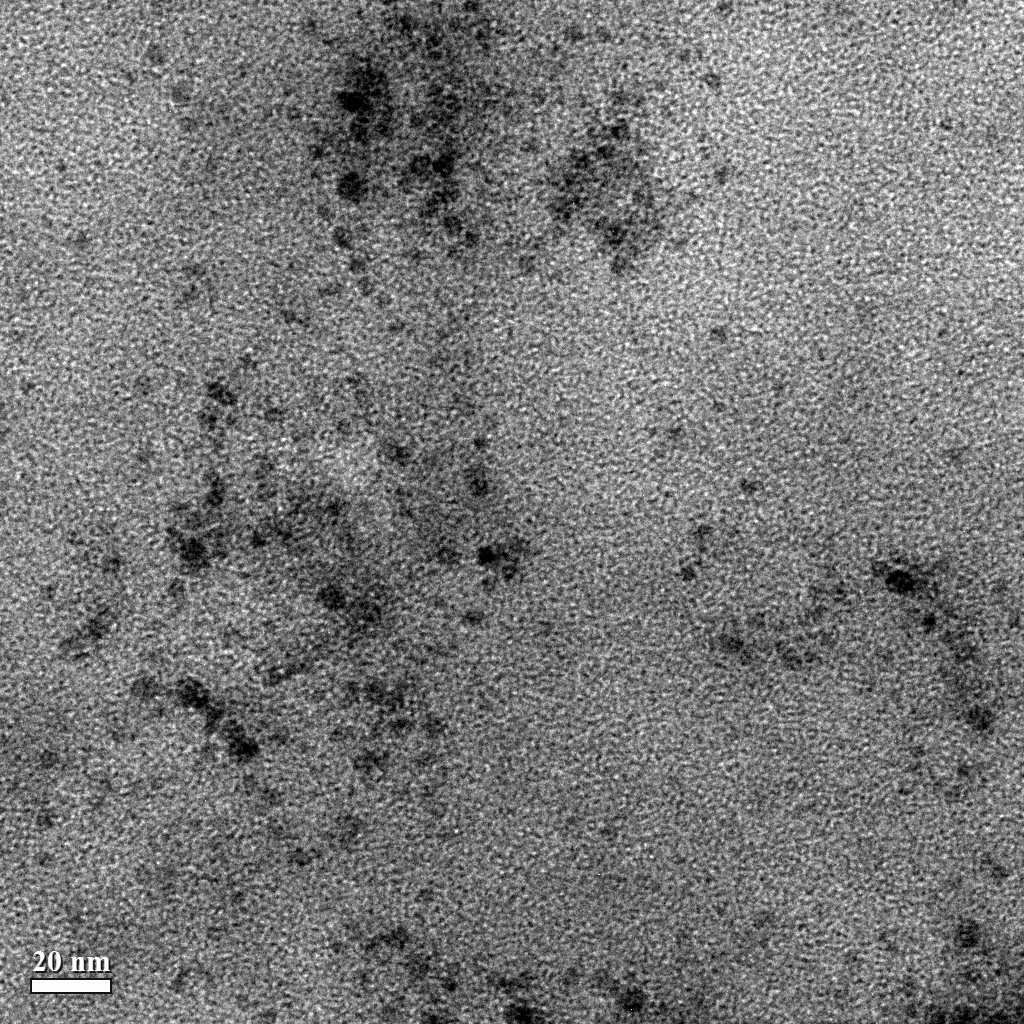


**Figure S26** The TEM picture of **Ru-LipM** (10 μM) assembly in PBS aqueous solution.


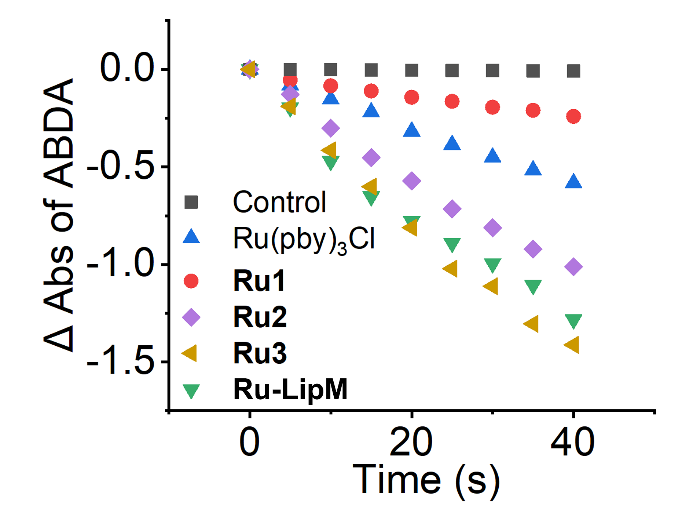


**Figure S27** ABDA photosensitized by **Ru1**–**Ru3** and **Ru-LipM** under irradiation (450 nm, 17 mW cm^−2^), in aerated PBS buffer (pH 7.4) as shown by the decrease in the absorptive maxima of ABDA (380 nm).


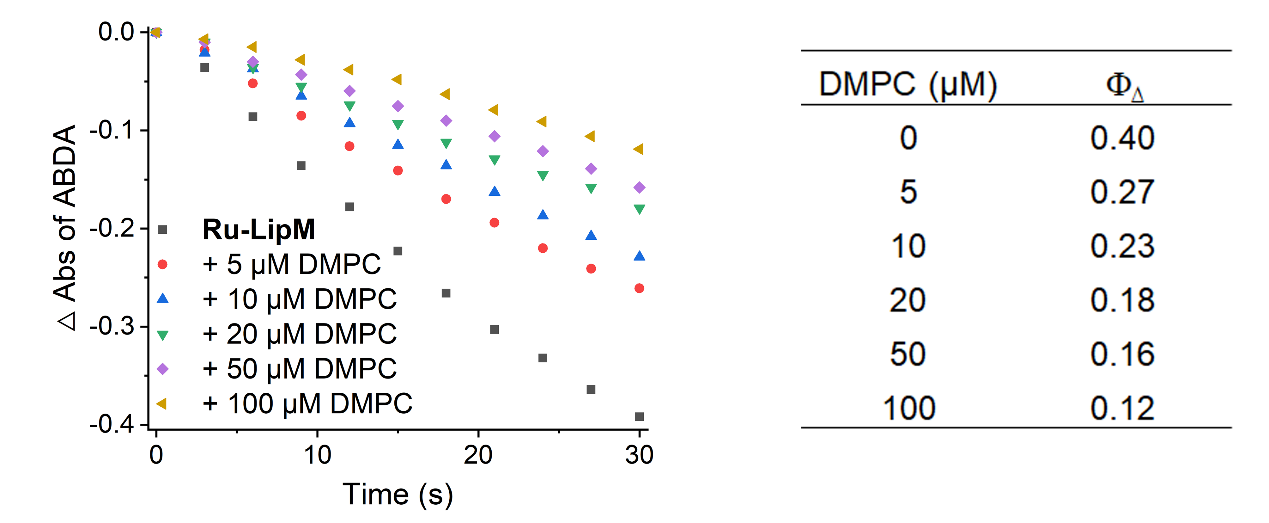


**Figure S28** ABDA photosensitized by **Ru-LipM** mixed DMPC at indicated concentrations under irradiation (450 nm, 17 mW cm^−2^,) in aerated 2% Tween-20 buffer.


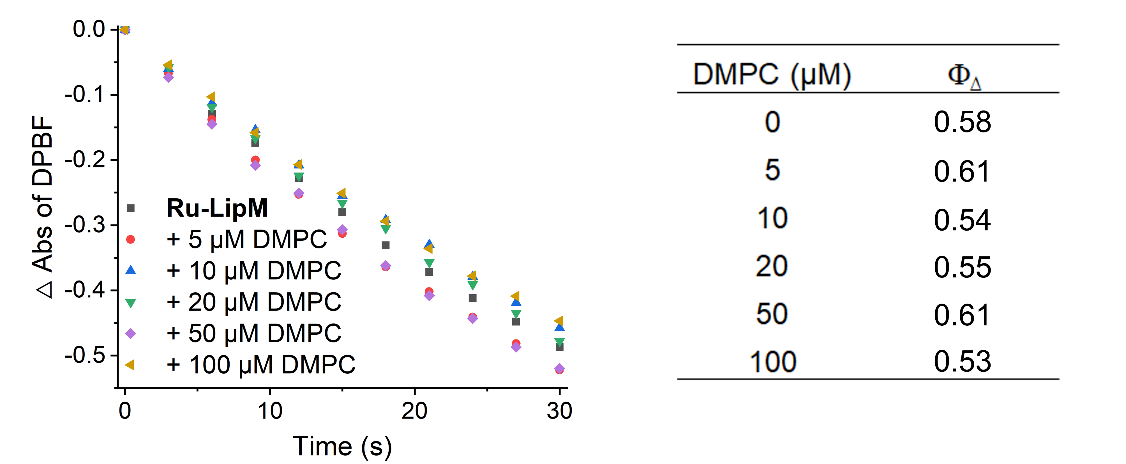


**Figure S29** DPBF photosensitized by **Ru-LipM** mixed DMPC at indicated concentrations under irradiation (450 nm, 17 mW cm^−2^,) in aerated DMSO (pH 7.4).


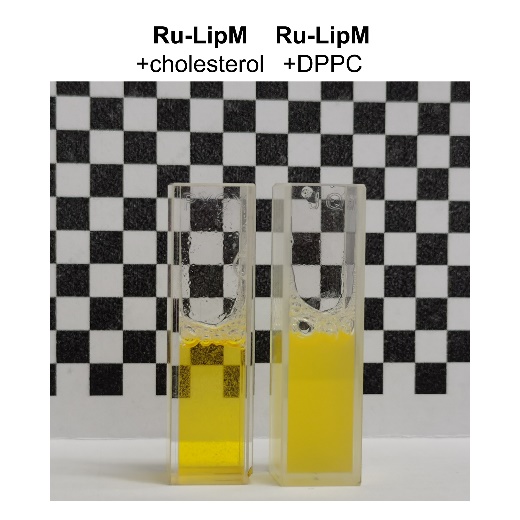


**Figure S30** The turbidity assays of DPPC/cholesterol (100 μM) in the presence of **Ru-LipM** (10 μM) in 2% Tween-20 buffer. The images were recorded by a digital camera.


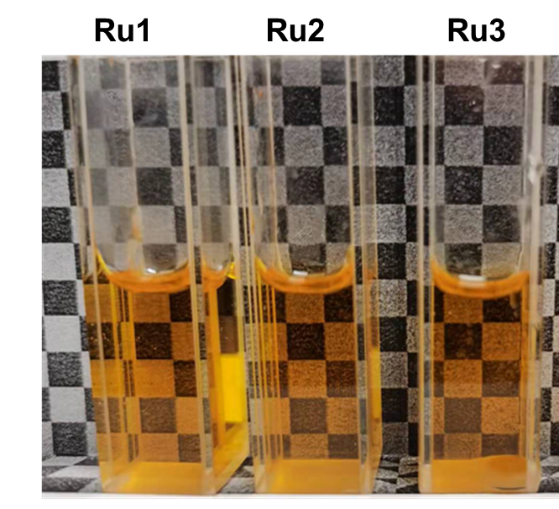


**Figure S31** The turbidity assays of DMPC (100 μM) and **Ru1**–**Ru3** (10 μM) in 2% Tween-20 buffer recorded by digital camera.


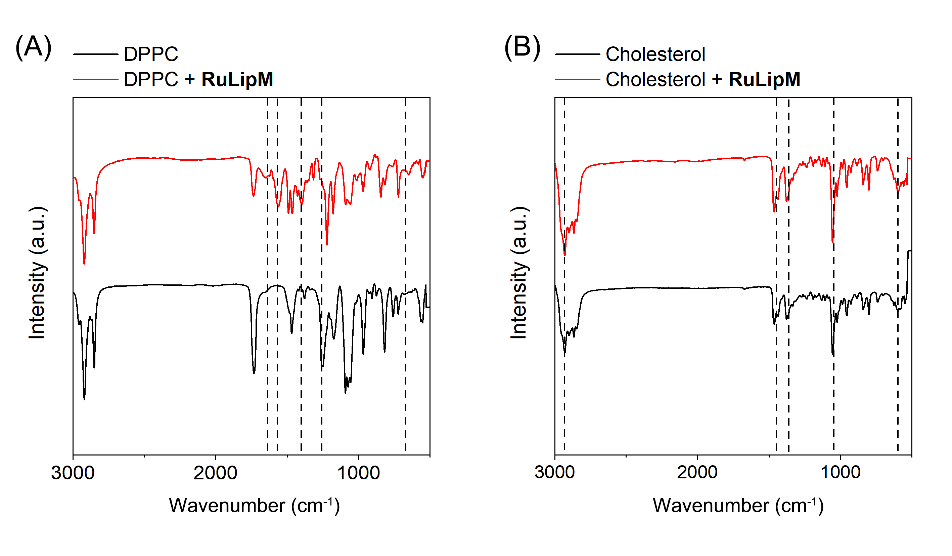


**Figure S32** IR spectrum of (A) DPPC and DPPC/**Ru-LipM** mixture, (B) cholesterol and cholesterol/**Ru-LipM** mixture.

**Figure S33** IR spectrum of DMPC and DMPC/**Ru1–Ru3** mixtures.


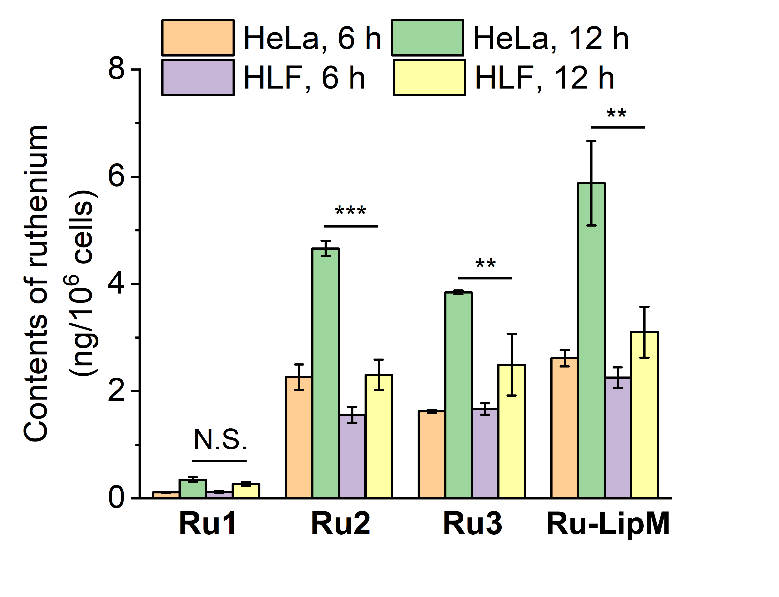


**Figure S34** The uptake of **Ru-LipM** and **Ru1–Ru3** (10 μM) in neoplastic HeLa cells and normal HLF cells monitored by ICP-MS. Error bars: S.D., n=3. **p* < 0.05, ***p* < 0.01, ****p* < 0.001 by the unpaired Student's two-tailed *t* test.


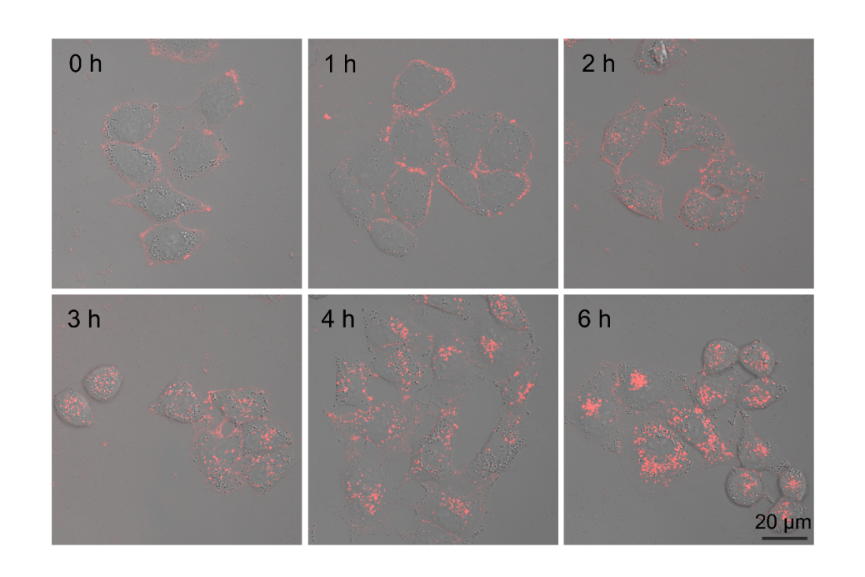


**Figure S35** The time-dependent uptake of **Ru-LipM** (10 μM) monitored by laser scanning confocal microscope. λ_ex_ = 488 nm; λ_em_ = 607 ± 20 nm.


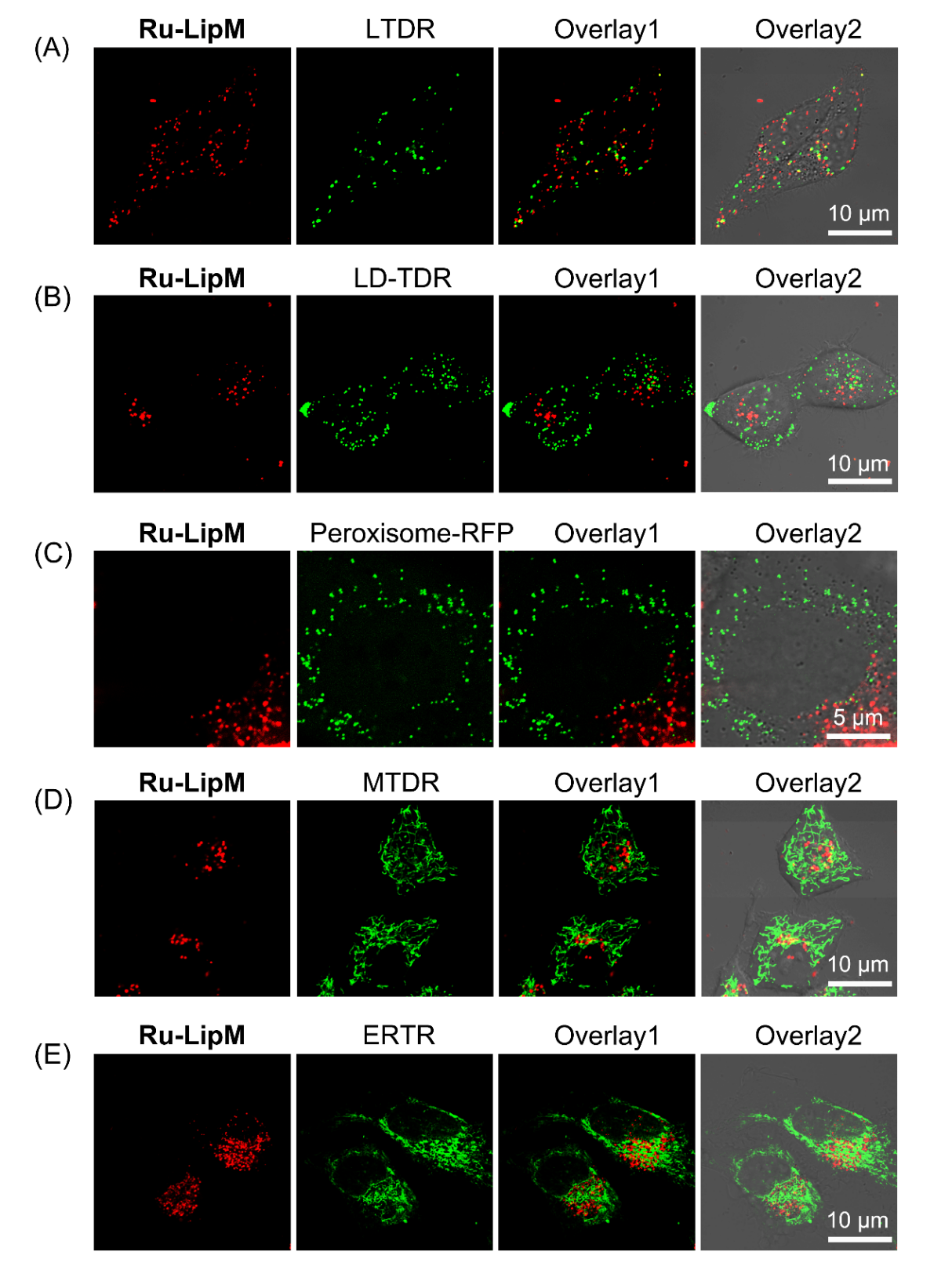


**Figure S36** Representative confocal images of HeLa cells exposed to **Ru-LipM** (10 μM) upon 6 h treatment and colocalized with (A) LTDR (200 nM, 15 min), (B) LD-TDR (1×, 30 min), (C) Peroxiasome-RFP (2 μg, transfection for 12 h), (D) MTDR (150 nM, 15 min) and (E) ERTR (1×, 30 min). **Ru-LipM**: λ_ex_ = 488 nm, λ_em_ = 607 ± 20 nm. LTDR: λ_ex_ = 633 nm, λ_em_ = 668 ± 20 nm. LD-TDR: λ_ex_ = 633 nm, λ_em_ = 660 ± 20 nm. Peroxiasome-RFP: λ_ex_ = 561 nm, λ_em_ = 599 ± 20 nm. MTDR: λ_ex_ = 633 nm, λ_em_ = 665 ± 20 nm. ERTR: λ_ex_ = 561 nm; λ_em_ = 597 ± 20 nm.


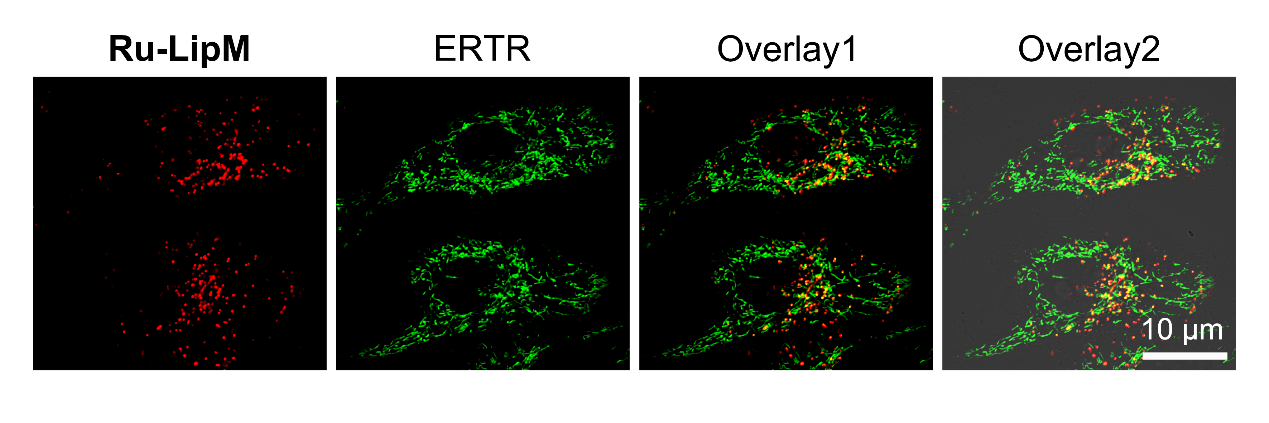


**Figure S37** Representative confocal images of HeLa cells exposed to **Ru-LipM** (10 μM) upon 12 h treatment and colocalized with ERTR (1×, 30 min). **Ru-LipM**: λ_ex_ = 488 nm; λ_em_ = 607 ± 20 nm. ERTR: λ_ex_ = 561 nm; λ_em_ = 597 ± 20 nm.





**Figure S38** Distribution of **Ru-LipM** (10 μM) in HeLa cells after 6 h or 12 h was measured by ICP-MS with error bars representing the standard deviation (n=3).


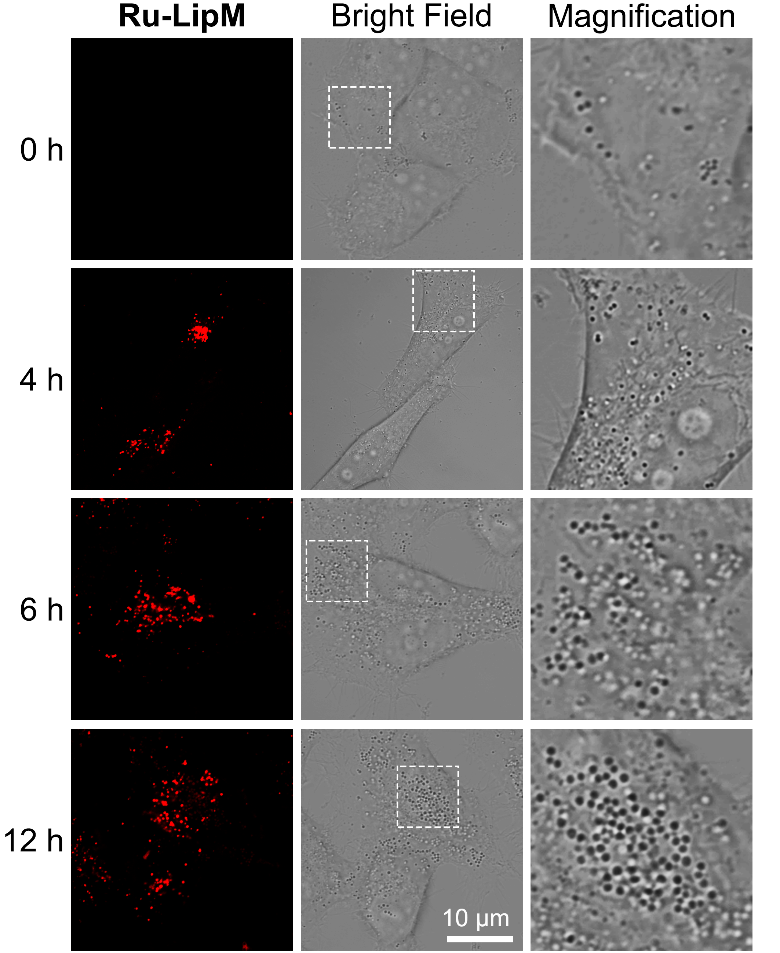


**Figure S39** Representative confocal images of HeLa cells exposed to **Ru-LipM** (10 μM) at different time intervals. **Ru-LipM**: λ_ex_ = 488 nm; λ_em_ = 607 ± 20 nm.


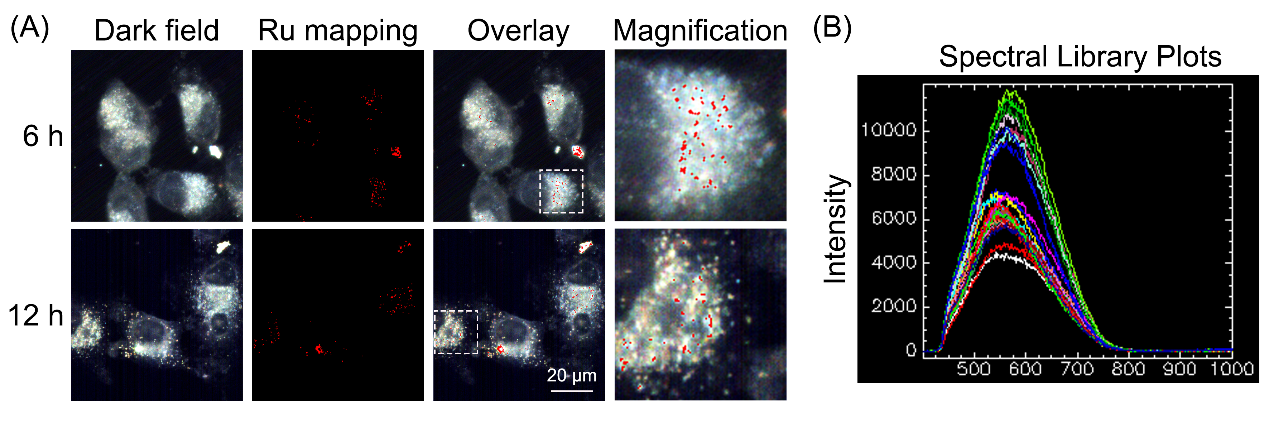


**Figure S40** (A) Hyperspectral images of HeLa cells exposed to **Ru-LipM** (10 μM). (B) The corresponding scattering spectra of **Ru-LipM**.





**Figure S41** Triglyceride (TG) amount of HeLa cells exposed to **Ru-LipM** (10 μM) upon different time intervals measured by ELISA kit. Error bars: S.D., n=3. **p* < 0.05, ***p* < 0.01, ****p* < 0.001 by the unpaired Student's two-tailed *t* test.


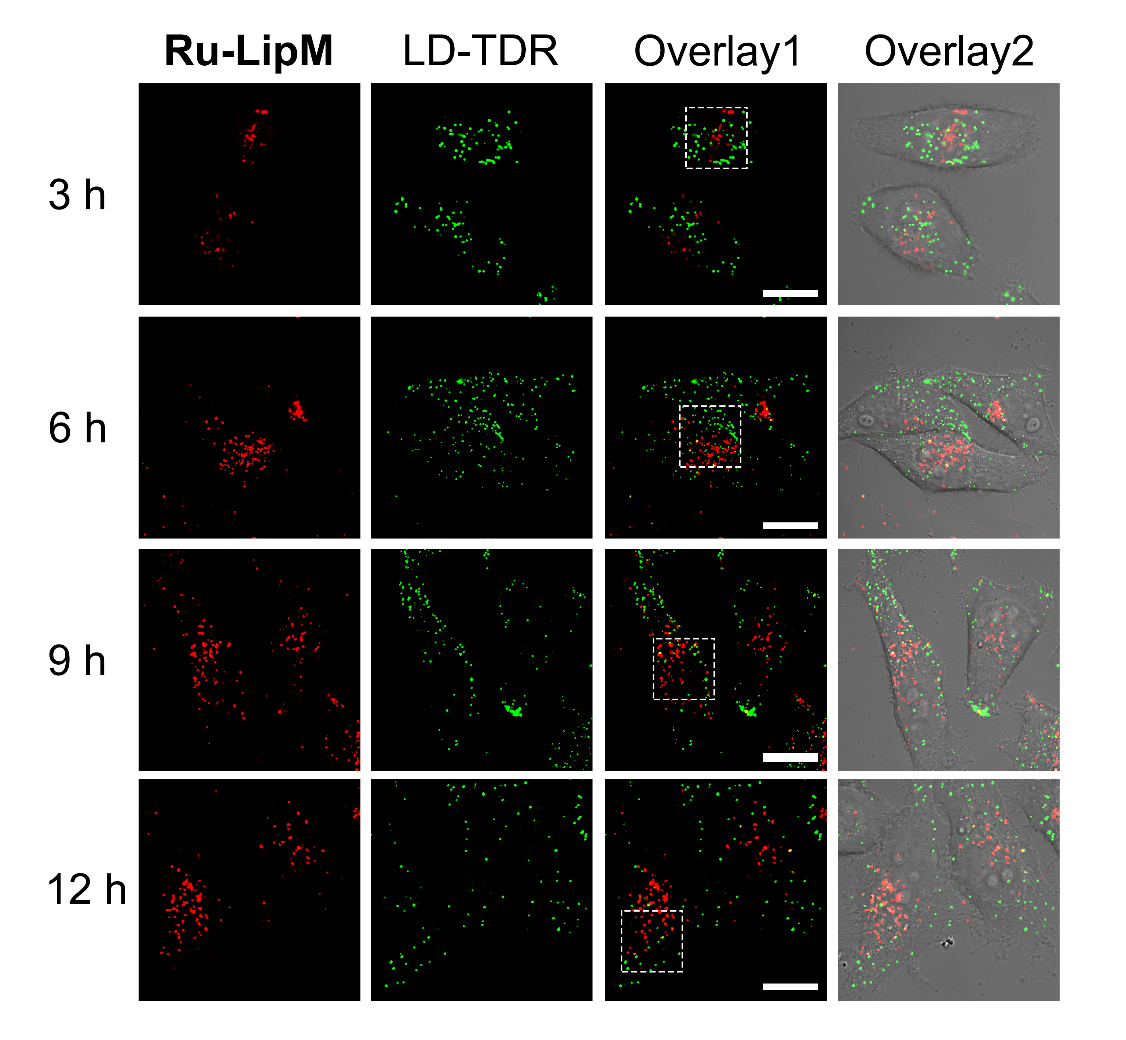


**Figure S42** Representative confocal images of HeLa cells treated with **Ru-LipM** (10 μM) at different time intervals. The samples are costained with LD-TDR (1×, 30 min). **Ru-LipM**: λ_ex_ = 488 nm; λ_em_ = 607 ± 20 nm. LD-TDR: λ_ex_ = 633 nm, λ_em_ = 660 ± 20 nm.


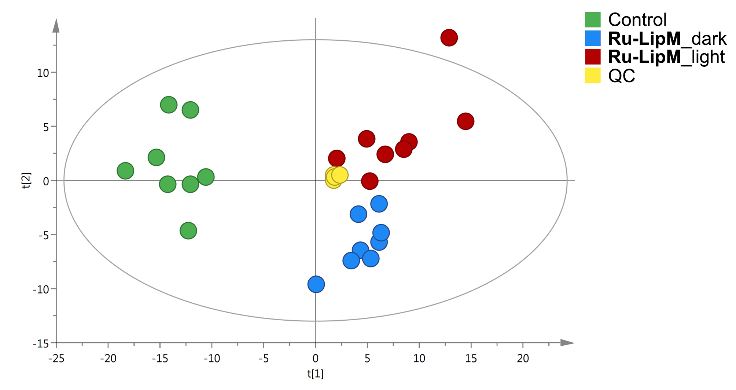


**Figure S43** Score plots of PCA model, obtained from control, **Ru-LipM**_dark (10 μM) and **Ru-LipM**_light (10 μM) groups. Quality control (QC) indicates the good stability and reproducibility. Irradiation condition: 450 nm, 17 mW cm^−2^, 2 J cm^−2^.


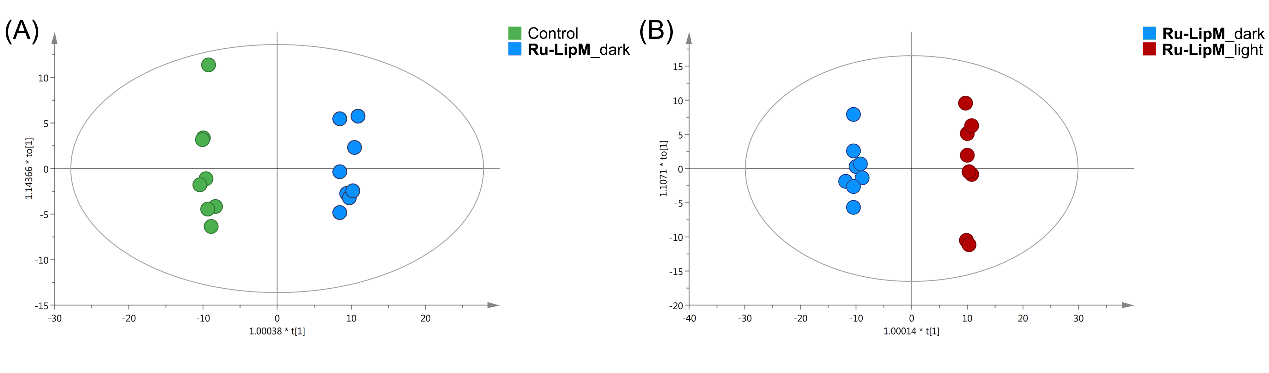


**Figure S44** Score plots of OPLS-DA model, obtained from (A) **Ru-LipM**_dark (10 μM) group *vs* control group and (B) **Ru-LipM**_light (10 μM) group *vs* **Ru-LipM**_dark (10 μM) group. Irradiation condition: 450 nm, 17 mW cm^−2^, 2 J cm^−2^.





**Figure S45** Statistics of lipid classes and lipid species. AcCa: Acyl carnitine, Cer: Ceramide, CerG2NAc1: Simple glucosylceramide series, CerP: Ceramides phosphate, CL: Cardiolipins, DG: Diglyceride, GD2, GM3, GT: Gangliosides, Hex1Cer, Hex2Cer, Hex3Cer: Hexose ceramides, LPC: Lysophosphatidylcholine, LPE: Lysophosphatidylethanolamine, LPG: Lysophosphatidylglycerol, LPI: Lysophosphatidylinositol, LPS: Lysophosphatidylserine, MG: Monoglyceride, PA: Phosphatidic acid, PC: Phosphatidylcholine, PE: Phosphatidylethanolamine, PG: Phosphatidylglycerol, phSM: Phytosphingosine, PI: Phosphatidylinositol, PIP, PIP3: Phosphatidylinositol bisphosphates, PS: Phosphatidylserine, SM: Sphingomyelin, SPH: Sphingosine, SPHP: Sphingomyelin, ST: Steroid, TG: Triglyceride, WE: Wax esters.


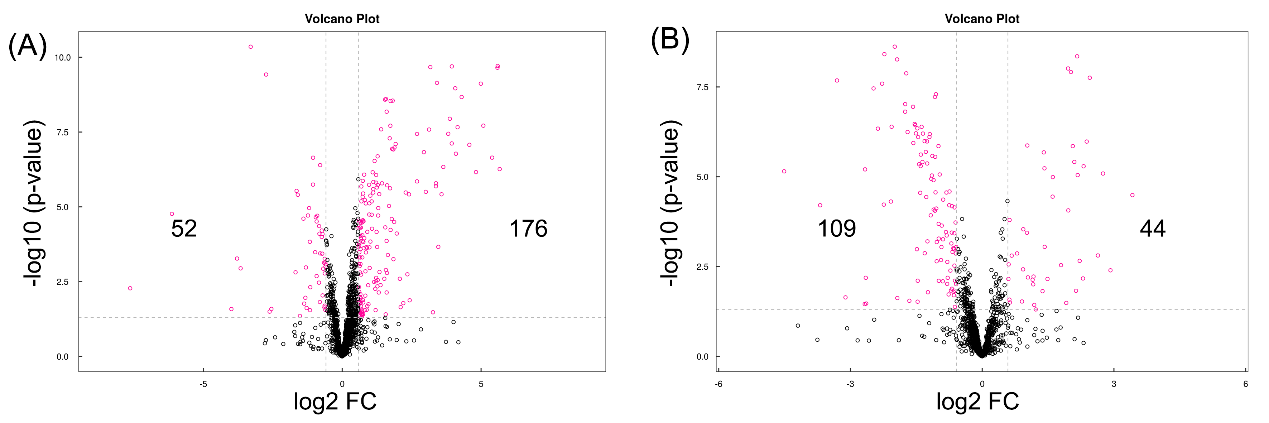


**Figure S46** Volcano plots showing the differential lipid species in HeLa cells treated with **Ru-LipM**. (A) **Ru-LipM**_dark (10 μM) group *vs* control group. (B) **Ru-LipM**_light (10 μM) group *vs* **Ru-LipM**_dark (10 μM) group. Irradiation condition: 450 nm, 17 mW cm^−2^, 2 J cm^−2^. Standard: The variable importance (VIP) > 1, fold change (FC) >1.5 or <0.67, *P* value < 0.05.


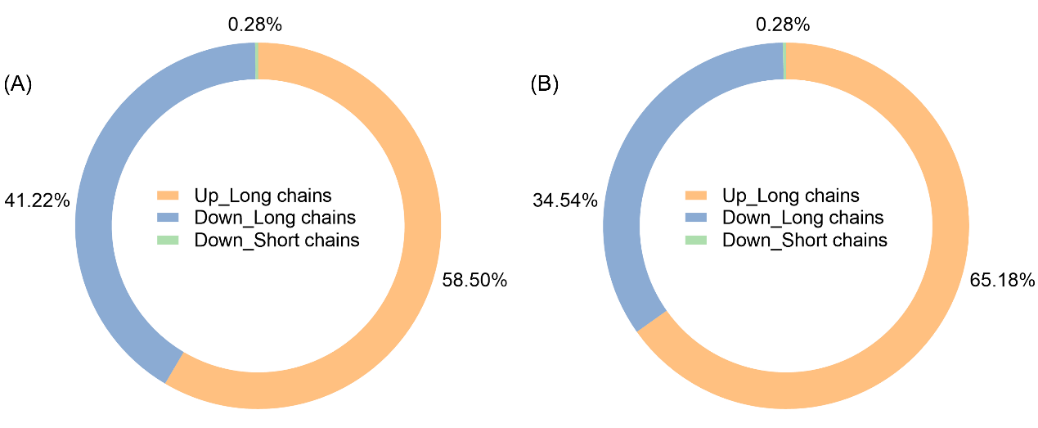


**Figure S47** The characteristic of carbon chain length of altered lipid species after **Ru-LipM** treatments. (A) **Ru-LipM**_dark (10 μM) group *vs* control group. (B) **Ru-LipM**_light (10 μM) group *vs* **Ru-LipM**_dark (10 μM) group. Irradiation condition: 450 nm, 17 mW cm^−2^, 2 J cm^−2^. Long chains: C atoms > 12, short chains: 1 ≤ C atoms <6.


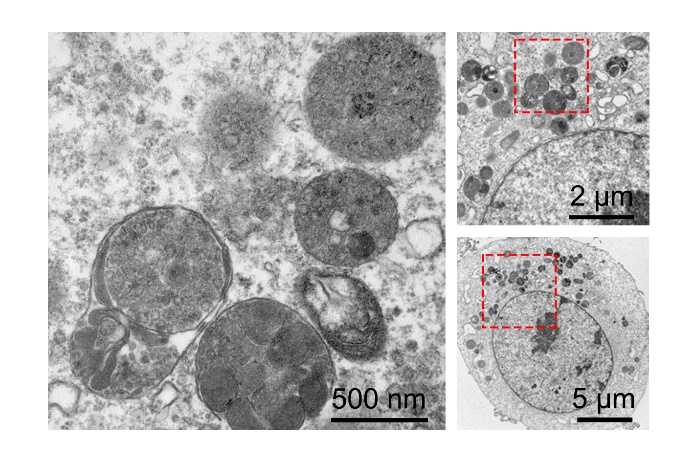


**Figure S48** TEM images of HeLa cells treated with 10 μM **Ru-LipM** for 12 h and then incubated for another 1 h after light irradiation. Irradiation condition: 450 nm, 17 mW cm^−2^, 2 J cm^−2^.





**Figure S49** Quantitative analysis for expressions of LC3, p62, ATG5, mTOR, NCOA4 and ferritin proteins in Figure 4C. Irradiation condition: 450 nm, 17 mW cm^−2^, 2 J cm^−2^. Error bars: S.D., n=3. **p* < 0.05, ***p* < 0.01, ****p* < 0.001 by the unpaired Student's two-tailed *t* test.


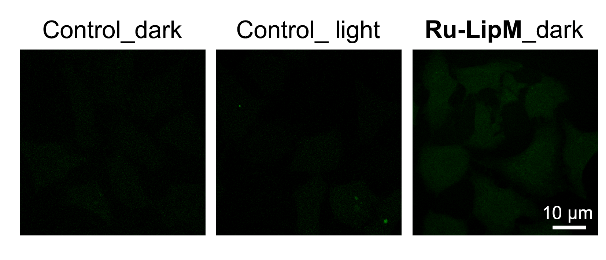


**Figure S50** Impact of **Ru-LipM** (10 μM) on ROS levels by H_2_DCFDA staining and confocal microscopy. DCF: λ_ex_ = 488 nm; λ_em_ = 525 ± 20 nm. Irradiation condition: 450 nm, 17 mW cm^−2^, 2 J cm^−2^.





**Figure S51** Impact of **Ru-LipM** (10 μM) on the ratios of cellular NADP^+^/NADPH. Irradiation condition: 450 nm, 17 mW cm^−2^, 2 J cm^−2^. Error bars: S.D., n=3. **p* < 0.05, ***p* < 0.01, ****p* < 0.001 by the unpaired Student's two-tailed *t* test.


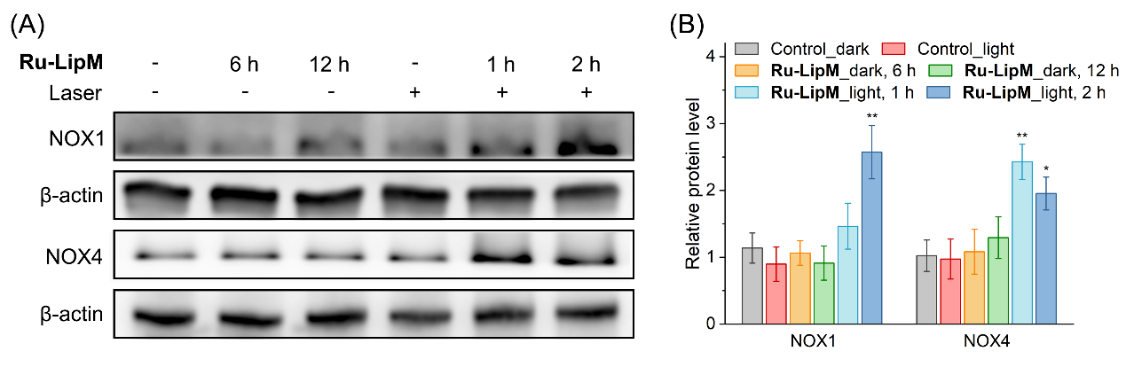


**Figure S52** (A) Western analysis and (B) quantitative analysis for expressions of NOX1 and NOX4 proteins. Irradiation condition: 450 nm, 17 mW cm^−2^, 2 J cm^−2^. Error bars: S.D., n=3. **p* < 0.05, ***p* < 0.01, ****p* < 0.001 by the unpaired Student's two-tailed *t* test.





**Figure S53** Quantitative analysis for expressions of GPX4 and ACSL4 in Figure 4H. Irradiation condition: 450 nm, 17 mW cm^−2^, 2 J cm^−2^. Error bars: S.D., n=3. **p* < 0.05, ***p* < 0.01, ****p* < 0.001 by the unpaired Student's two-tailed *t* test.





**Figure S54** Cell viability of HeLa cells determined upon co-incubation (24 h) with **Ru-LipM** and different inhibitors upon light irradiation. These inhibitors necrosulfonamide (10 μM), ferrostatin-1 (10 μM), disulfiram (4 μM), z-VAD-fmk (40 μM), 3-methyladenine (500 μM) and necrostatin-1 (50 μM) were administrated 1 h before irradiation. Irradiation condition: 450 nm, 17 mW cm^−2^, 2 J cm^−2^. Error bars: S.D., n=3. **p* < 0.05, ***p* < 0.01, ****p* < 0.001 by the unpaired Student's two-tailed *t* test.


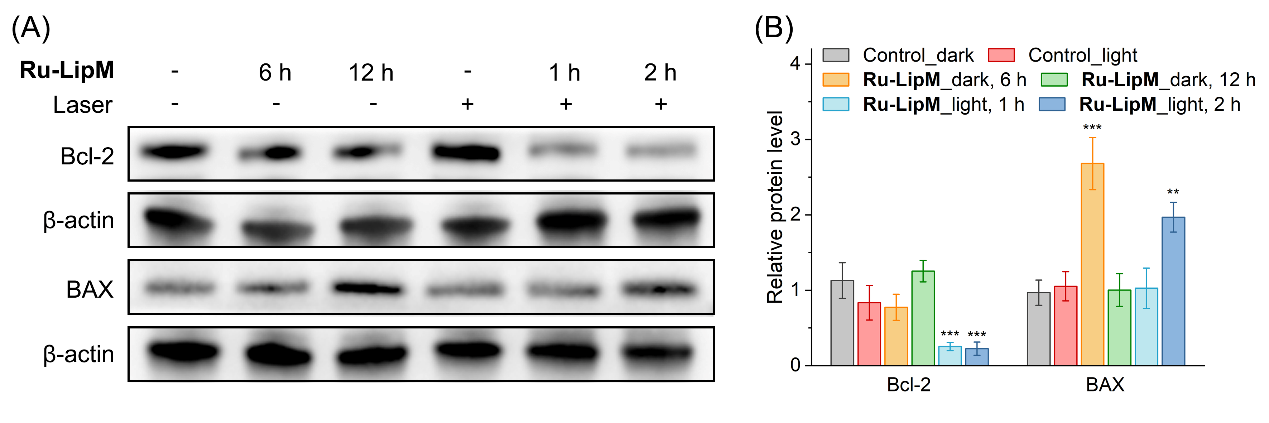


**Figure S55** (A) Western analysis and (B) quantitative analysis for expressions of Bcl-2 and BAX proteins. Irradiation condition: 450 nm, 17 mW cm^−2^, 2 J cm^−2^. Error bars: S.D., n=3. **p* < 0.05, ***p* < 0.01, ****p* < 0.001 by the unpaired Student's two-tailed *t* test.





**Figure S56** Quantitative analysis of mean fluorescence intensity (MFI) for etco-CRT in Figure 4I. Irradiation condition: 450 nm, 17 mW cm^−2^, 2 J cm^−2^. Error bars: S.D., n=3. **p* < 0.05, ***p* < 0.01, ****p* < 0.001 by the unpaired Student's two-tailed *t* test.





**Figure S57** Quantitative analysis of mean fluorescence intensity (MFI) for HMGB1 in Figure 4J. Irradiation condition: 450 nm, 17 mW cm^−2^, 2 J cm^−2^. Error bars: S.D., n=3. **p* < 0.05, ***p* < 0.01, ****p* < 0.001 by the unpaired Student's two-tailed *t* test.


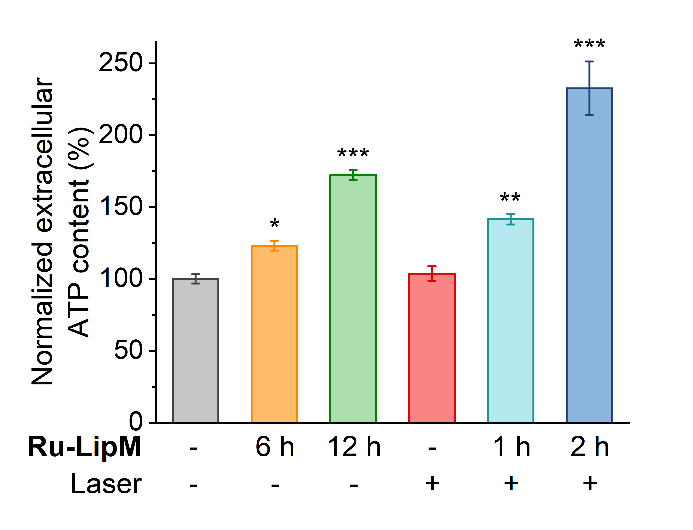


**Figure S58** Impact of **Ru-LipM** (10 μM) on the extracellular secretion of ATP. Irradiation condition: 450 nm, 17 mW cm^−2^, 2 J cm^−2^. Error bars: S.D., n=3. **p* < 0.05, ***p* < 0.01, ****p* < 0.001 by the unpaired Student's two-tailed *t* test.


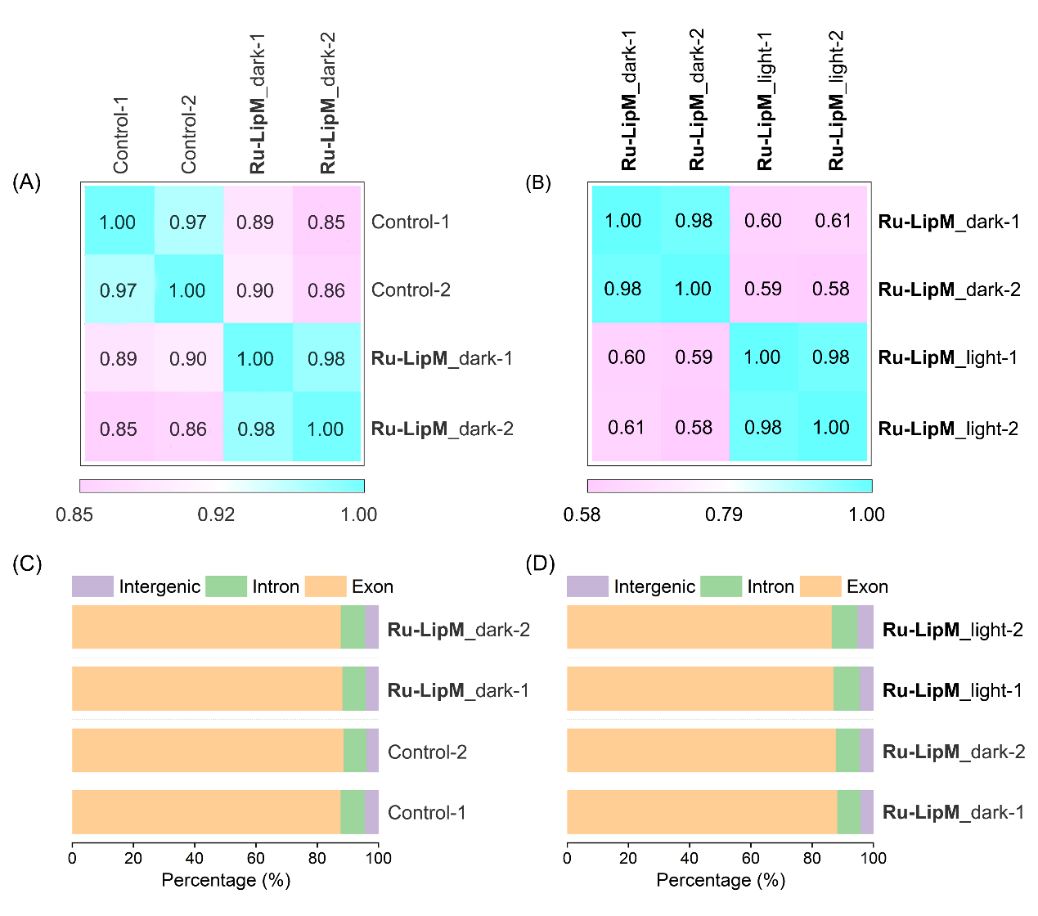


**Figure S59** (A−B) Heat map diagram of Pearson correlation coefficient and (C−D) genomic location of mapped transcripts between the RNA-seq samples. (A and C) **Ru-LipM**_dark (10 μM) group *vs* control group. (B and D) **Ru-LipM**_light (10 μM) group *vs* **Ru-LipM**_dark (10 μM) group. Irradiation condition: 450 nm, 17 mW cm^−2^, 2 J cm^−2^.


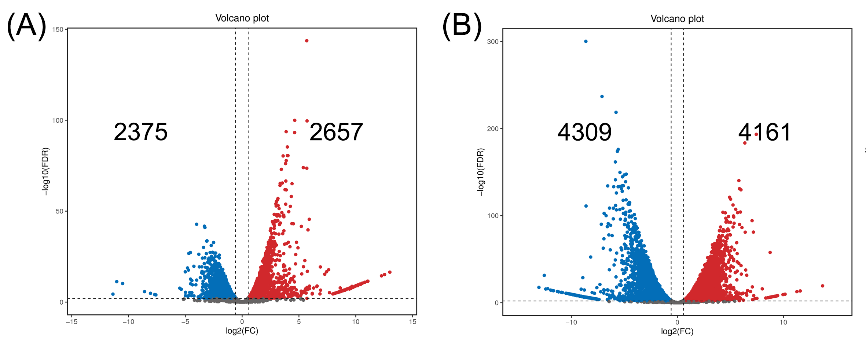


**Figure S60** Volcano plots showing the differential expression genes in HeLa cells treated with **Ru-LipM**_light (10 μM). (A) **Ru-LipM**_dark group *vs* control group. (B) **Ru-LipM**_light group *vs* **Ru-LipM**_dark group. Irradiation condition: 450 nm, 17 mW cm^−2^, 2 J cm^−2^. Standard: False Discovery Rate (FDR) < 0.01. FC > 1.5 or < 0.67.


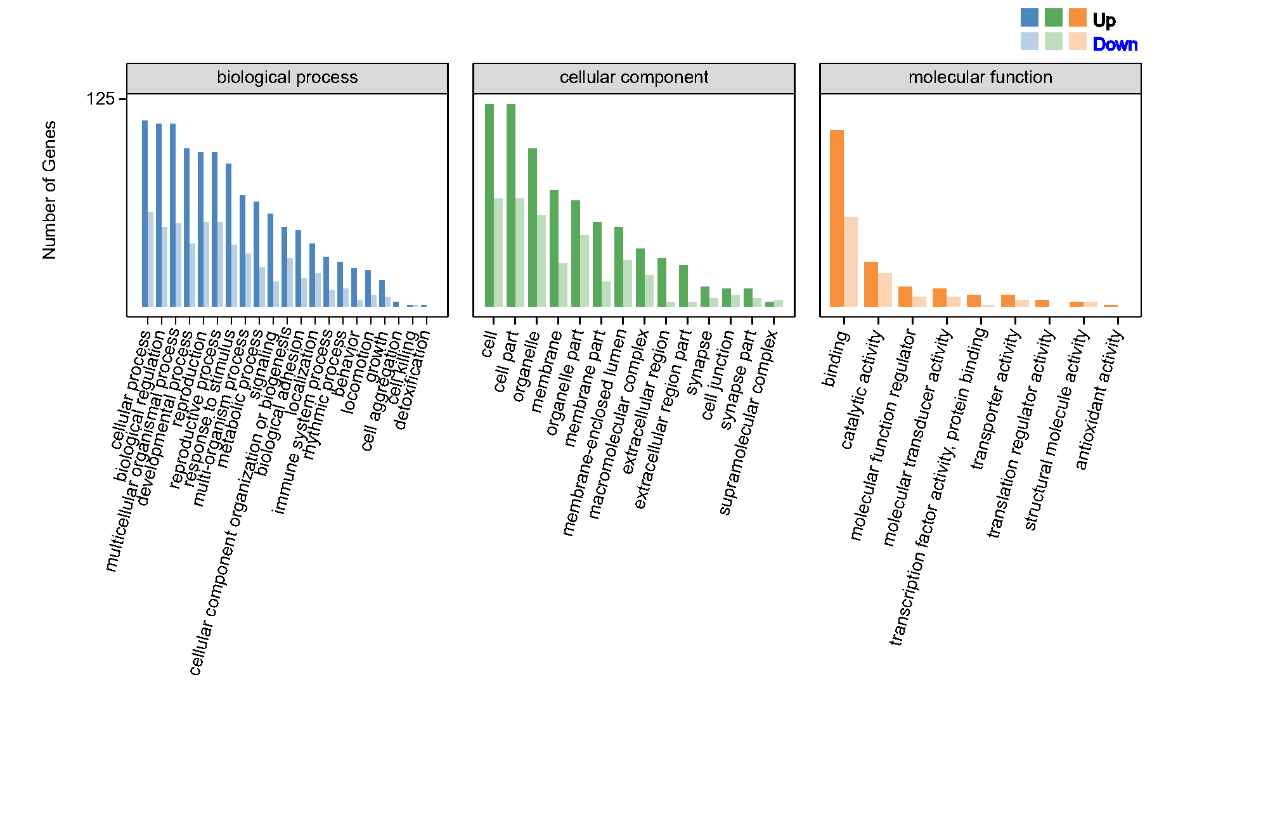


**Figure S61** Gene Ontology (GO) categorization of molecular function, cellular component and biological process induced by **Ru-LipM** treatment (10 μM). Standard: Observed genes > 2, FDR < 0.01 and FC > 1.5 or < 0.67.


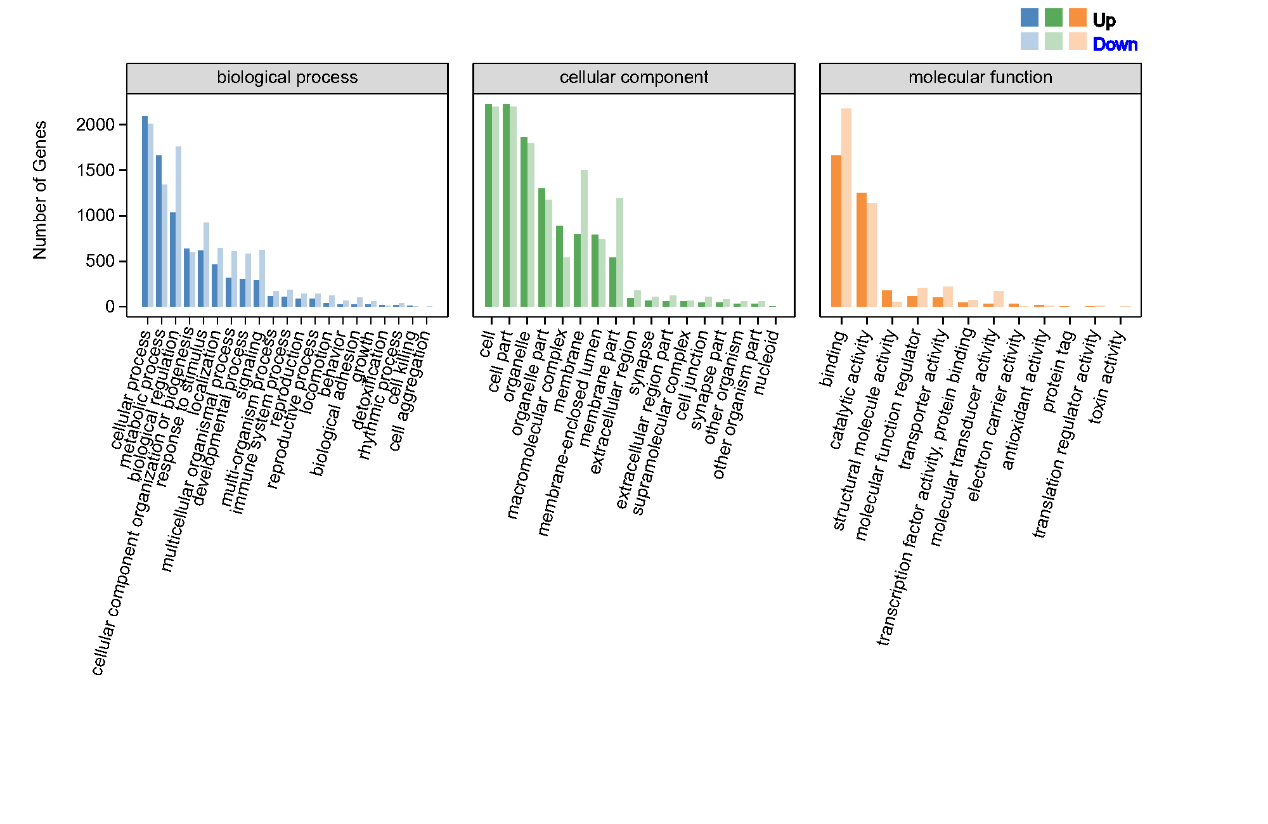


**Figure S62** GO categorization of molecular function, cellular component and biological process induced by **Ru-LipM** treatment (10 μM) upon irradiation. Irradiation condition: 450 nm, 17 mW cm^−2^, 2 J cm^−2^. Standard: Observed genes > 2, FDR < 0.01 and FC > 1.5 or < 0.67.





**Figure S63** Gene expressions of human leukocyte antigen (HLA)-A and transporter associated with antigen processing 1 (TAP1) induced by **Ru-LipM** treatment (10 μM). Irradiation condition: 450 nm, 17 mW cm^−2^, 2 J cm^−2^. Error bars: S.D., n=3. **p* < 0.05, ***p* < 0.01, ****p* < 0.001 by the unpaired Student's two-tailed *t* test.





**Figure S64** Quantitative analysis of corresponding M2- and M1-macrophages proportion in Figure 5F. Irradiation condition: 450 nm, 17 mW cm^−2^, 2 J cm^−2^. Error bars: S.D., n=3. **p* < 0.05, ***p* < 0.01, ****p* < 0.001 by the unpaired Student's two-tailed *t* test.


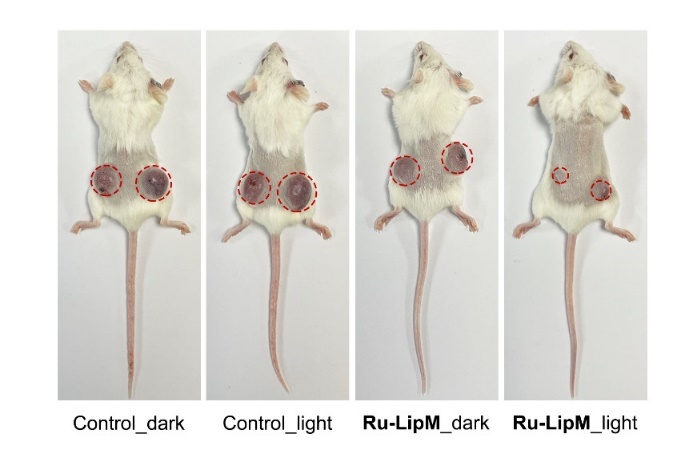


**Figure** **S65** The representative mice of different groups.





**Figure** **S66** The body weight curves of mice. Error bars: S.D., n=5.


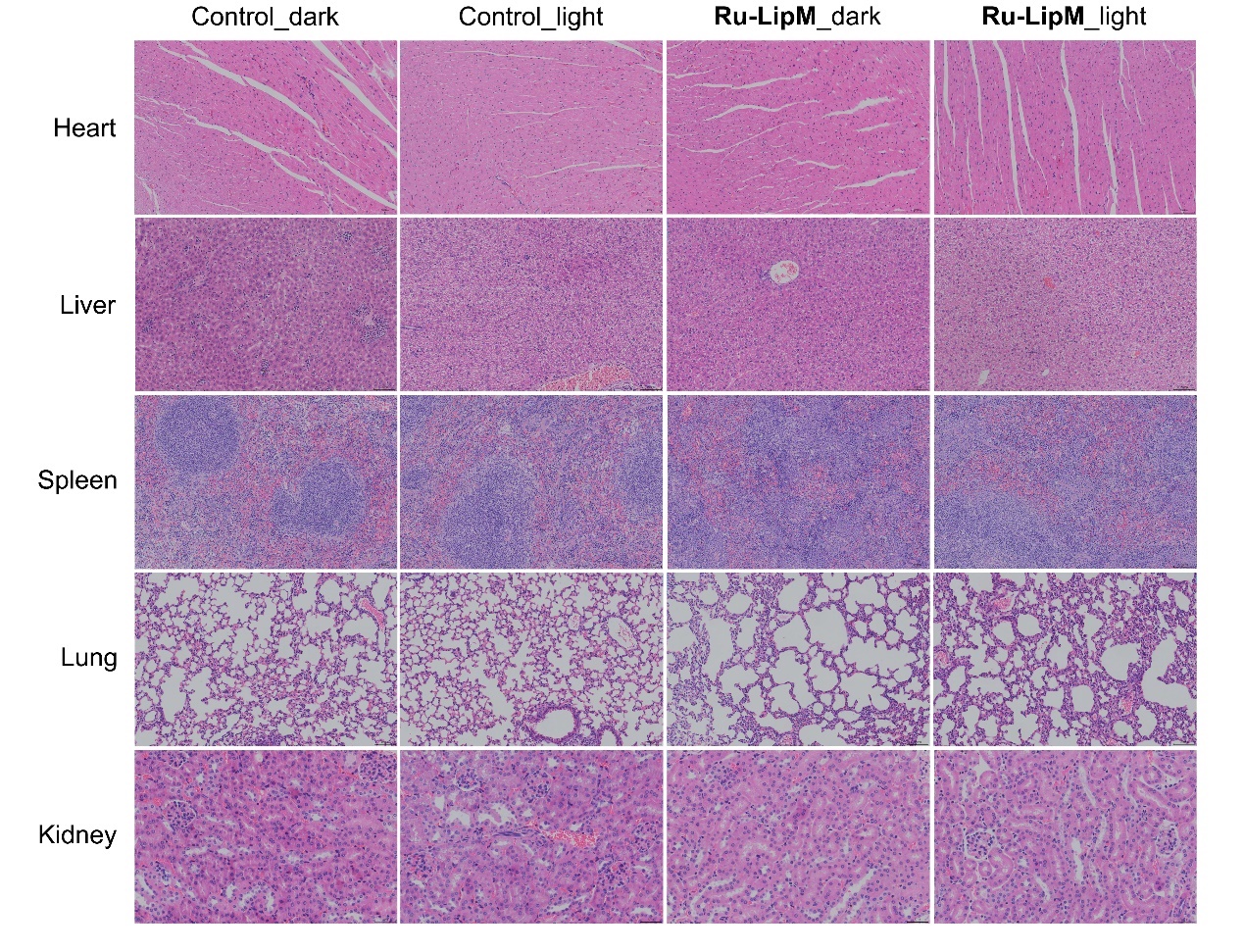


**Figure S67** H&E staining images of major organs collected from mice in different groups. The scale bar is 100 μm.





**Figure S68** Quantitative analysis of corresponding M2- and M1-macrophages proportion in Figure 6E. Irradiation condition: 450 nm, 17 mW cm^−2^, 2 J cm^−2^. Error bars: S.D., n=5. **p* < 0.05, ***p* < 0.01, ****p* < 0.001 by the unpaired Student's two-tailed *t* test.


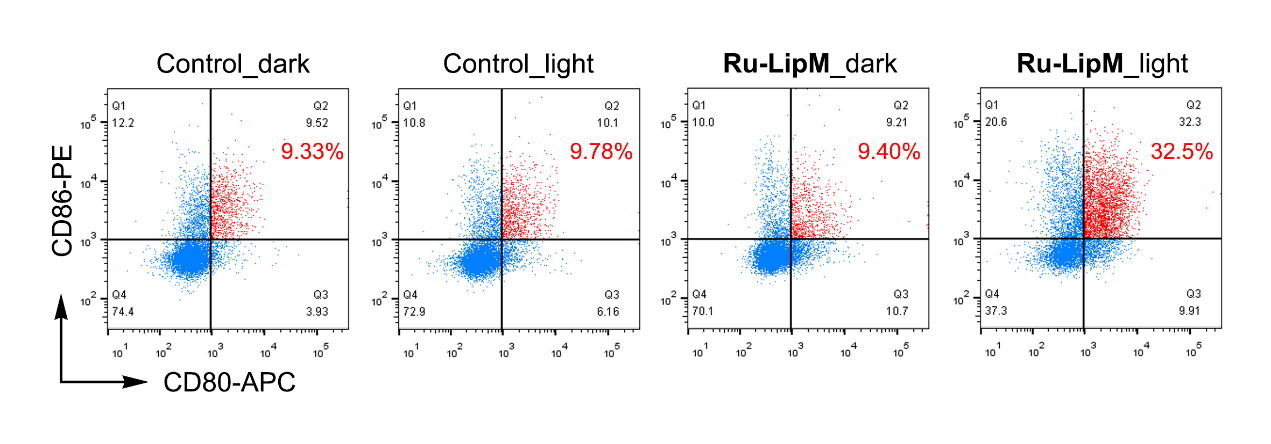


**Figure S69** Quantitatively detection of populations of CD86^+^CD80^+^ DCs (gating on CD11c^+^).


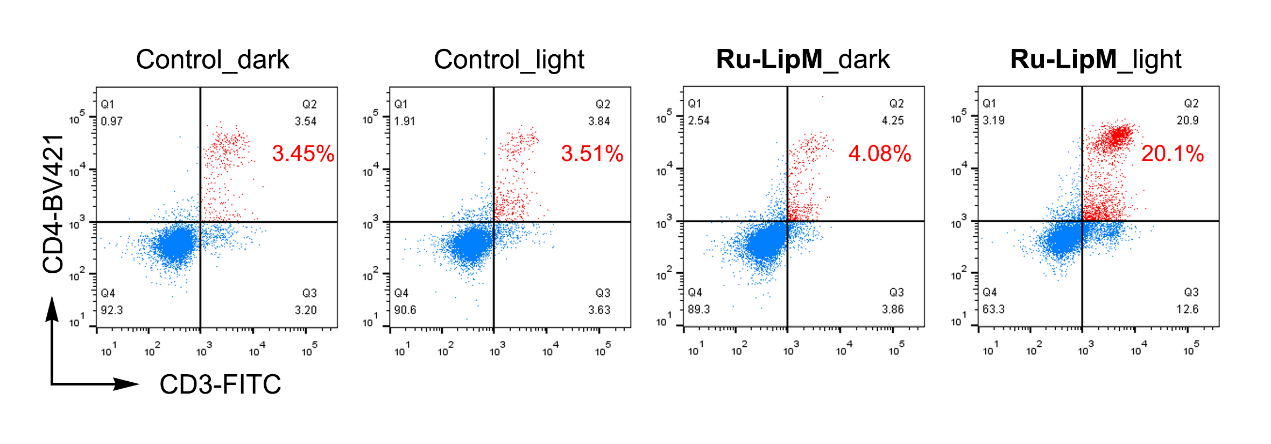


**Figure S70** Quantitatively detection of populations of CD3^+^CD4^+^ T cells.





**Figure S71** Quantitative analysis of populations of mature dendritic cells (DCs) (Figure S55), CD8^+^ T cells (Figure 6G), Foxp3^+^ T cells (Figure 6H) and CD4^+^ T cells (Figure S56). Irradiation condition: 450 nm, 17 mW cm^−2^, 2 J cm^−2^. Error bars: S.D., n=5. **p* < 0.05, ***p* < 0.01, ****p* < 0.001 by the unpaired Student's two-tailed *t* test.

Supplementary tables

**Table S1** Photophysical and photochemical properties of Ru(II) complexes

| Complexes | Solvent | λ_ex, max_ (nm) | λ_em, max_ (nm) | Lifetime (ns) | *Ф*_△_*^a^* |
| --- | --- | --- | --- | --- | --- |
| **Ru1** | CH_2_Cl_2_ | 263, 368 | 586 | 509.35 | - |
|  | CH_3_CN | 263, 367 | 602 | 397.93 | - |
|  | PBS | 264, 372 | - | - | 0.05 |
| **Ru2** | CH_2_Cl_2_ | 269, 400 | 592 | 1213.98 | - |
|  | CH_3_CN | 271, 397 | 596 | 425.20 | - |
|  | PBS | 285, 405 | 610 | 361.03 | 0.35 |
| **Ru3** | CH_2_Cl_2_ | 270, 401 | 590 | 1400.81 | - |
|  | CH_3_CN | 267, 397 | 598 | 427.47 | - |
|  | PBS | 286, 404 | 611 | 319.79 | 0.44 |
| **Ru-LipM** | CH_2_Cl_2_ | 264, 397 | 574 | 301.75 | - |
|  | CH_3_CN | 262, 395 | 589 | 301.59 | - |
|  | PBS | 266, 405 | 607 | 1203.35 | 0.40 |

*^a^* The *Φ*_∆_ were determined using [Ru(bpy)_3_]Cl_2_ in aerated H_2_O (*Φ*_∆_ = 0.18) as the reference.^[1]^

**Table S2** IC_50_ (μM) values of **Ru1**−**Ru3** in dark or light conditions*^a^*

| Cell lines | IC_50_ (μM) | | | | | | | | |
| --- | --- | --- | --- | --- | --- | --- | --- | --- | --- |
|  | **Ru1** | | | **Ru2** | | | **Ru3** | | |
|  | dark | light*^b^* | PI | dark | light*^b^* | PI | dark | light*^b^* | PI |
| HeLa | >50.0 | >50.0 | - | 4.17±0.29 | 0.54±0.02 | 7.7 | 37.2±4.88 | 3.16±0.42 | >11.8 |
| U14 | >50.0 | >50.0 | - | 4.38±0.45 | 0.17±0.01 | 25.8 | 30.9±5.42 | 2.97±0.28 | 10.4 |
| A549 | >50.0 | >50.0 | - | 13.5±0.33 | 0.73±0.06 | 18.5 | 26.9±5.61 | 5.07±0.26 | 5.3 |
| LLC | >50.0 | >50.0 | - | 6.92±0.69 | 0.95±0.17 | 7.3 | >50.0 | 3.89±0.44 | >12.9 |
| MDA-231 | >50.0 | >50.0 | - | 4.86±0.21 | 0.33±0.03 | 14.7 | 19.4±0.89 | 2.96±0.32 | 6.6 |
| 4T1 | >50.0 | >50.0 | - | 3.55±0.24 | 0.24±0.03 | 14.8 | >50.0 | 5.82±0.63 | >8.6 |
| MCF-10A | >50.0 | >50.0 | - | 2.69±0.28 | 0.35±0.05 | 7.7 | 46.4±3.75 | 5.33±0.29 | 8.7 |
| HLF | >50.0 | >50.0 | - | 6.68±0.23 | 0.31±0.04 | 21.5 | >50.0 | 6.57±0.33 | >7.6 |

*^a^* Data are presented as the means ± standard deviations (SD), and antiproliferative activity was assessed after 72 h of incubation. *^b^* Irradiated condition: 450 nm, 17 mW cm^−2^, 2 J cm^−2^.

**Table S3** Lifetimes of DMPC/**Ru-LipM** (x) hybrids.

| x | **Ru-LipM** lifetimes (ns) |
| --- | --- |
| 0% | 531.25 |
| 5% | 544.34 |
| 10% | 624.50 |
| 20% | 775.36 |

**Table S4** Lipid compositions upon **Ru-LipM**-treatments with/without irradiation.

| Lipid class abbreviation | Lipid composition (%) | | | Lipid class full name | LIPID MAPS category^[2]^ |
| --- | --- | --- | --- | --- | --- |
|  | Control | **Ru-LipM**_dark | **Ru-LipM**_light |  |  |
| PIP | 0.003 | 0.004 | 0.005 | Phosphatidylinositol phosphates | Glycerophospholipids (GP) |
| LPG | 0.004 | 0.005 | 0.004 | Lysophosphatidylglycerol | Glycerophospholipids (GP) |
| LPI | 0.005 | 0.005 | 0.005 | Lysophosphatidylinositol | Glycerophospholipids (GP) |
| LPS | 0.01 | 0.014 | 0.012 | Lysophosphatidylserine | Glycerophospholipids (GP) |
| PIP3 | 0.016 | 0.014 | 0.029 | Phosphatidylinositol triphosphates | Glycerophospholipids (GP) |
| LPE | 0.174 | 0.265 | 0.168 | Lysophosphatidylethanolamine | Glycerophospholipids (GP) |
| LPC | 0.425 | 0.474 | 0.434 | Lysophosphatidylcholine | Glycerophospholipids (GP) |
| PA | 0.564 | 0.688 | 0.739 | Phosphatidic acid | Glycerophospholipids (GP) |
| PG | 1.549 | 1.722 | 1.608 | Phosphatidylglycerol | Glycerophospholipids (GP) |
| PI | 2.048 | 2.532 | 2.452 | Phosphatidylinositol | Glycerophospholipids (GP) |
| PS | 2.339 | 3.149 | 3.236 | Phosphatidylserine | Glycerophospholipids (GP) |
| CL | 2.796 | 4.08 | 3.291 | Cardiolipins | Glycerophospholipids (GP) |
| PC | 34.251 | 25.644 | 26.479 | Phosphatidylcholine | Glycerophospholipids (GP) |
| PE | 37.848 | 40.688 | 39.691 | Phosphatidylethanolamine | Glycerophospholipids (GP) |
| SPHP | 0.001 | 0.001 | 0 | Sphingomyelin | Sphingolipids (SP) |
| GD2 | 0.009 | 0.011 | 0.01 | Gangliosides | Sphingolipids (SP) |
| GT3 | 0.009 | 0.012 | 0.012 | Gangliosides | Sphingolipids (SP) |
| CerG2GNAc1 | 0.011 | 0.014 | 0.017 | Simple Glc series | Sphingolipids (SP) |
| GM3 | 0.016 | 0.022 | 0.03 | Gangliosides | Sphingolipids (SP) |
| CerP | 0.125 | 0.145 | 0.14 | Ceramides phosphate | Sphingolipids (SP) |
| Hex3Cer | 0.231 | 0.255 | 0.27 | Trihexosylceramide | Sphingolipids (SP) |
| Hex2Cer | 0.501 | 0.598 | 0.61 | Dihexosylceramide | Sphingolipids (SP) |
| Hex1Cer | 0.856 | 0.973 | 0.965 | Monohexosylceramide | Sphingolipids (SP) |
| SPH | 0.947 | 1.442 | 1.494 | Sphingosine | Sphingolipids (SP) |
| phSM | 1.293 | 1.354 | 1.319 | Phytosphingosine | Sphingolipids (SP) |
| Cer | 1.918 | 2.712 | 2.905 | Ceramide | Sphingolipids (SP) |
| SM | 7.357 | 7.701 | 8.014 | Sphingomyelin | Sphingolipids (SP) |
| MG | 0.011 | 0.019 | 0.007 | Monoglyceride | Glycerolipids (GL) |
| TG | 1.427 | 1.737 | 1.832 | Triglyceride | Glycerolipids (GL) |
| DG | 1.612 | 1.981 | 2.246 | Diglyceride | Glycerolipids (GL) |
| WE | 0.038 | 0.059 | 0.061 | Wax esters | Fatty Acyls (FA) |
| AcCa | 0.299 | 0.141 | 0.121 | Acyl carnitine | Fatty Acyls (FA) |
| ST | 1.307 | 1.539 | 1.793 | Steroid | Sterol lipids (ST) |

**Table S5** Functions of differential lipid classes

| Lipid Class | Content analysis | | function |
| --- | --- | --- | --- |
|  | **Ru-LipM**_dark *vs* control | **Ru-LipM**_light *vs* **Ru-LipM**_dark |  |
| LPE | Up | Down | Plasma membrane component and fatty acid biosynthesis^[3]^ |
| LPG | Up | Down | Membrane homeostasis^[4]^ and adipose storage^[5]^ |
| LPS | Up | - | Immune-modulatory functions^[6]^ |
| PIP3 | Down | Up | Insulin signaling modulation^[7]^ |
| PC | Down | - | Major component of plasma lipoprotein, lipid signal transmission^[8]^ |
| SPH | Up | - | Calcium mobilization and cytoskeleton organization^[9]^ |
| Cer | Up | - | Stress signal transduction^[10]^ and programmed cell death activation^[11]^ |
| GM3 | Up | Up | Components of lipid rafts and signal transduction^[12]^ |
| GT3 | Up | - |  |
| CerG2GNAc1 | Up | - | Inflammatory disease^[13]^ |
| SPHP | Down | Down | Critical components of biological membranes and lipid rafts^[14]^ |
| Hex2Cer | Up | - | Induction of type 2 diabetes^[15]^ |
| MG | Up | Down | Major forms of storage and transport of fatty acids^[16]^ |
| TG | Up | - |  |
| AcCa | Down | - | Fatty acid β-oxidation^[17]^ |
| WE | Up | - | Lipid transformation and waterproofing in marine organisms^[18]^ |

**Table S6** Statistics of RNA-Seq for the control and the **Ru-LipM**-treated groups

| Sample | Mapped Reads |
| --- | --- |
| Control-1 | 36,184,940 (96.88%) |
| Control-2 | 39,993,884 (97.11%) |
| **Ru-LipM**_dark-1 | 42,524,883 (97.23%) |
| **Ru-LipM**_dark-2 | 39,386,470 (95.87%) |
| **Ru-LipM**_light-1 | 42,574,799 (96.46%) |
| **Ru-LipM**_light-2 | 38,457,801 (96.78%) |

**Table S7** The primer sequences

| m-NF-κB-F | CCTGCTTCTGGAGGGTGATG |
| --- | --- |
| m-NF-κB-R | GCCGCTATATGCAGAGGTGT |
| m-iNOS-F | ATCTTTGCCACCAAGATGGCCTGG |
| m-iNOS-R | TTCCTGTGCTGTGCTACAGTTCCG |
| m-STAT3-F | TTCCTGTGCTGTGCTACAGTTCCG |
| m-STAT3-R | TCCTCACATGGGGGAGGTAG |
| m-Msr-F | TGCCTCTGTGCTTGCTGC |
| m-Msr-R | CCACTGTCACCGCGTCTT |
| h-HLA-A-F | ACCCTCGTCCTGCTACTCTC |
| h-HLA-A-R | CTGTCTCCTCGTCCCAATACT |
| h-TAP1-F | TGCCCCGCATATTCTCCCT |
| h-TAP1-R | CACCTGCGTTTTCGCTCTTG |
| m-β-actin-F | GGTCCACACCCGCCACCAG |
| m-β-actin-R | CACATGCCGGAGCCGTTGTC |
| h-GAPDH-F | GGGAAACTGTGGCGTGAT |
| h-GAPDH-R | GAGTGGGTGTCGCTGTTGA |

References

[1] J. M. Wessels, C. S. Foote, W. E. Ford, M. A. J. Rodgers, *Photochem. Photobiol.* **1997**, *65*, 96-102.

[2] E. Fahy, S. Subramaniam, H. A. Brown, C. K. Glass, A. H. J. Merrill, R. C. Murphy, C. R. H. Raetz, D. W. Russell, Y. Seyama, W. Shaw, T. Shimizu, F. Spener, G. van Meer, M. S. VanNieuwenhze, S. H. White, J. L. Witztum, E. A. Dennis, *J. Lipid Res.* **2005**, *46*, 839-861.

[3] Y. Yamamoto, T. Sakurai, Z. Chen, N. Inoue, H. Chiba, S.-P. Hui, *Nutrients* **2022**, *14*, 579.

[4] C. Subramanian, M.-K. Yun, M. M. Frank, C. O. Rock, *J. Biol. Chem.* **2023**, *299*, 104863.

[5] T. Tsutsumi, Y. Okamoto, S. Yamakawa, C. Bingjun, A. Ishihara, T. Tanaka, A. Tokumura, *Life Sci.* **2016**, *157*, 208-216.

[6] J. Omi, K. Kano, J. Aoki, *Cell Biochem. Biophys.* **2021**, *79*, 497-508.

[7] S. R. Ande, S. Mishra, *Biochem. Biophys. Res. Commun.* **2009**, *390*, 1023-1028.

[8] L. K. Cole, J. E. Vance, D. E. Vance, *BBA-Mol. Cell Biol. L.* **2012**, *1821*, 754-761.

[9] S. Spiegel, *J. Leukocyte Biol.* **1999**, *65*, 341-344.

[10] Y. A. Hannun, *Science* **1996**, *274*, 1855-1859.

[11] L. M. Obeid, C. M. Linardic, L. A. Karolak, Y. A. Hannun, *Science* **1993**, *259*, 1769-1771.

[12] C.-L. Schengrund, *Trends Biochem. Sci.* **2015**, *40*, 397-406.

[13] Q. Yang, Z. Huang, N. Diao, J. Tang, X. Zhu, Q. Guo, K. Chao, X. Gao, *Ann. Transl. Med.* **2022**, *10*, 1062.

[14] Z. Li, T. K. Hailemariam, H. Zhou, Y. Li, D. C. Duckworth, D. A. Peake, Y. Zhang, M.-S. Kuo, G. Cao, X.-C. Jiang, *BBA-Mol. Cell Biol. L.* **2007**, *1771*, 1186-1194.

[15] Y. K. Tham, K. S. Jayawardana, Z. H. Alshehry, C. Giles, K. Huynh, A. A. T. Smith, J. Y. Y. Ooi, S. Zoungas, G. S. Hillis, J. Chalmers, P. J. Meikle, J. R. McMullen, *Diabetes* **2020**, *70*, 255-261.

[16] M. Alves-Bezerra, D. E. Cohen, *Compr. Physiol.* **2017**, *8*, 1-8.

[17] M. M. Adeva-Andany, N. Carneiro-Freire, M. Seco-Filgueira, C. Fernández-Fernández, D. Mouriño-Bayolo, *Mitochondrion* **2019**, *46*, 73-90.

[18] J. R. Sargent, R. R. Gatten, R. McIntosh, *Mar. Chem.* **1977**, *5*, 573-584.
